# Appraising associations between signature lipidomic biomarkers and digestive system cancer risk: novel evidences from a prospective cohort study of UK Biobank and Mendelian randomization analyses

**DOI:** 10.1186/s12944-024-02053-9

**Published:** 2024-02-28

**Authors:** Yuanlin Sun, Donghui Cao, Yang Zhang, Yanhua Wu, Zhifang Jia, Yingnan Cui, Dongming Li, Xueyuan Cao, Jing Jiang

**Affiliations:** 1 Department of Gastrocolorectal Surgery, General Surgery Center, The First Hospital of Jilin University, Changchun, 130021 Jilin Province China; 2 Department of Clinical Epidemiology, The First Hospital of Jilin University, Changchun, 130021 Jilin Province China

**Keywords:** Signature lipidomic biomarkers, Digestive system cancer risk, Polygenic risk score, Linear and nonlinear Mendelian randomization analysis, Phenotypic associations, Causal associations

## Abstract

**Background:**

The roles of serum lipids on digestive system cancer (DSC) risk were still inconclusive. In this study, we systematically assessed indicative effects of signature lipidomic biomarkers (high-density lipoprotein cholesterol (HDL-C), low-density lipoprotein cholesterol (LDL-C), and triglycerides (TG)) on DSC (oesophagus, stomach, colorectal, liver, gallbladder, and pancreas cancers) risk.

**Methods:**

HDL-C, LDL-C, and TG concentration measurements were respectively analyzed with enzyme immunoinhibition, enzymatic selective protection, and GPO-POD methods in AU5800 supplied from Beckman Coulter. The diagnoses of DSCs were coded using International Classification of Diseases, Tenth Revision (ICD-10) codes updated until October 2022 in the UK Biobank (UKB). In this study, we assessed phenotypic association patterns between signature lipidomic biomarkers and DSC risk using restricted cubic splines (RCSs) in multivariable-adjusted Cox proportional hazards regression models. Moreover, linear and nonlinear causal association patterns of signature lipidomic biomarkers with DSC risk were determined by linear and nonlinear Mendelian randomization (MR) analyses.

**Results:**

A median follow-up time of 11.8 years was recorded for 319,568 participants including 6916 DSC cases. A suggestive independent nonlinear phenotypic association was observed between LDL-C concentration and stomach cancer risk (*P*_nonlinearity_ < 0.05, *P*_overall_ < 0.05). Meanwhile, a remarkable independent linear negative phenotypic association was demonstrated between HDL-C concentration and stomach cancer risk (*P*_nonlinearity_ > 0.05, *P*_overall_ < 0.008 (0.05/6 outcomes, Bonferroni-adjusted *P*)), and suggestive independent linear positive associations were observed between HDL-C concentration and colorectal cancer risk, and between TG concentration and gallbladder cancer risk (*P*_nonlinearity_ > 0.05, *P*_overall_ < 0.05). Furthermore, based on nonlinear and linear MR-based evidences, we observed an suggestive independent negative causal association (hazard ratio (HR) per 1 mmol/L increase: 0.340 (0.137-0.843), *P* = 0.020) between LDL-C and stomach cancer risk without a nonlinear pattern (Quadratic* P* = 0.901, Cochran Q *P* = 0.434). Meanwhile, subgroup and stratified MR analyses both supported the category of LDL-C ≥ 4.1 mmol/L was suggestively protective against stomach cancer risk, especially among female participants (HR: 0.789 (0.637-0.977), *P* = 0.030) and participants aged 60 years or older (HR: 0.786 (0.638-0.969),* P* = 0.024), and the category of TG ≥ 2.2 mmol/L concluded to be a suggestive risk factor for gallbladder cancer risk in male participants (HR: 1.447 (1.020-2.052),* P* = 0.038) and participants aged 60 years or older (HR: 1.264 (1.003-1.593),* P* = 0.047).

**Conclusions:**

Our findings confirmed indicative roles of signature lipidomic biomarkers on DSC risk, notably detecting suggestive evidences for a protective effect of high LDL-C concentration on stomach cancer risk, and a detrimental effect of high TG concentration on gallbladder cancer risk among given participants.

**Supplementary Information:**

The online version contains supplementary material available at 10.1186/s12944-024-02053-9.

## Introduction

The prevalence of digestive system cancers (DSCs) including oesophagus, stomach, colorectal, liver, gallbladder and pancreas cancers, is estimated to be above 5 million new cases (26.4% of new cancer cases) and over 3.6 million deaths (36.3% of cancer deaths) in 2020, imposing heavy healthcare burdens on both individuals and society [1]. Since there are differences among the various types of DSCs in terms of their pathogenesis, the characteristic DSC prevention is more challenging. Such being the cases, implementing targeted risk assessments of common risk markers is crucial for DSC prevention.

Dysfunctional lipid metabolism is related to the pathogenesis and development of DSCs through various mechanisms. On the one hand, during the malignant transformation of normal cells and cancer cell proliferation, more energy is required. This results in unusual activation of the cholesterol de novo synthesis pathway, causing abnormal expression of signal transduction factors, and an alteration in the way cancer cells acquire cholesterol to adapt to survival conditions [2, 3]. However, the pathogenesis and development of DSC share functional signaling pathways with lipid metabolism [4–7]. As the cardinal measurable metrics of lipid metabolism, serum lipid variations directly shed light on the basic functional state of body lipid metabolism, which makes it possible for serum lipid concentration detection to provide predictive values for DSCs. A prospective cohort study based on Danish cohorts reported that a low high-density lipoprotein cholesterol (HDL-C) level was associated with higher prevalence of cancer risk [8]. Besides, low-density lipoprotein cholesterol (LDL-C), and triglycerides (TG) concentrations have been reported to be positively associated with the incidence of colorectal cancer, and HDL-C concentration is negatively correlated with the incidence of colorectal cancer [9]. However, there are only observational studies available n the associations between serum lipids and the risk of DSCs, which tends to be influenced by the failure to recognize the independent effects of serum lipid concentration on the risk of DSCs, as well as a lack of adequate corrections for confounding factors, thus obtaining unstable phenotypic association findings. In addition, owing to the potential for selective bias and reverse causalities in observational studies [10], the authentic causal effects of serum lipids on the risk of DSCs needs to be further investigated.

In Mendelian randomization (MR) analyses, genetic variations are typically used as instrumental variables to explore causal relationships between exposures and outcomes, mimicking the principle of random subject assignment in traditional randomized control trials (RCT), where risk alleles are randomly assigned to individuals during gamete formation [11]. As such, MR studies are inclined to estimate causal associations genetically without selective bias and confounding bias. Nonlinear MR method, parallel with traditional linear MR method demonstrating the linear average causal effects of exposures on the outcomes, was used to identify the shape of the causal associations between exposures and outcomes [12]. Although some studies preliminarily extrapolated serum lipids on genetically predicting the risk of colorectal cancer [13] as well as pancreas cancer [14] from the perspective of linear function with conventional MR methods, we further expanded estimates of linear and nonlinear causal patterns of signature lipidomic biomarkers on the risk of six DSCs with linear and nonlinear MR analyses.

Based on a large-scale prospective study involving over 300,000 participants of UK Biobank (UKB), our study evaluated the linear and nonlinear phenotypic association patterns between signature lipidomic biomarkers and the risk of DSCs. Moreover, linear and nonlinear MR analyses were further employed to reveal putative causal patterns of signature lipidomic biomarkers on the risk of DSCs. Age- and sex-specific subgroup and stratified MR analyses further suggested phenotypic and genetic associations of stratified signature lipidomic biomarkers with the risk of DSCs.

## Methods

### Study design and participants

**Fig. 1:**
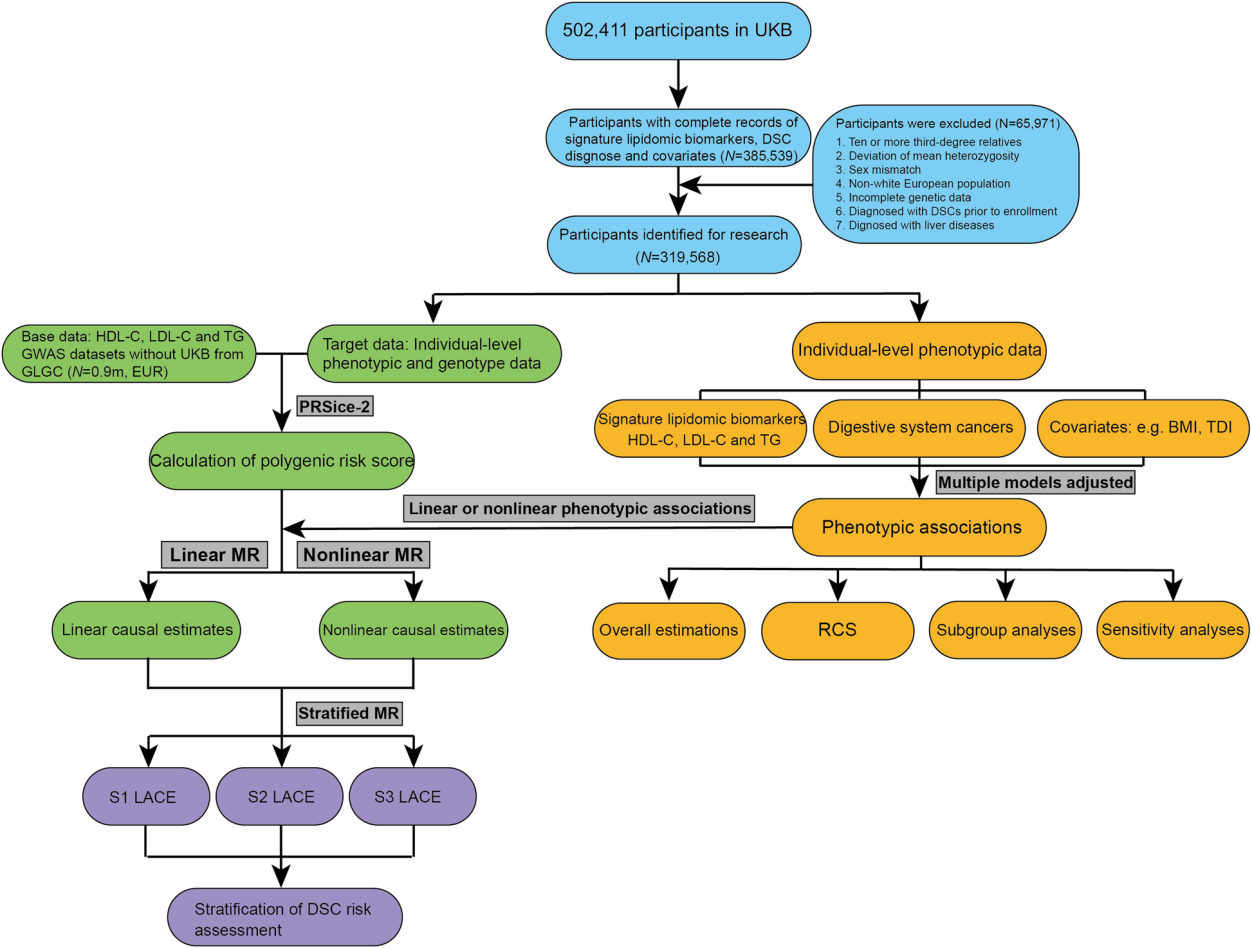
Flow charts of the study. RCS, restricted cubic splines; LACE, local average causal effects

The process of this study was illustrated in Fig. 1. UKB is a large-scale health-based interethnic prospective study aimed at assessing all genomic data and phenotypic data (health outcomes, baseline data, quantitative analyses of biological samples, online questionnaires, electrocardiogram activity monitoring, cognitive tests, and multimodality imaging data) of more than 500,000 volunteers (about 0.8% of the total British population) aged 40 to 69 years, and tracks records their health and medical records for decades. North West Multisite Research Ethics Committee (11/NW/0382) granted approval to the UK Biobank and informed consent was obtained prior to participation by all participants [15]. In this study, we excluded 116,872 participants without complete records of signature lipidomic biomarkers, DSC diagnosis and covariates, and 65,971 participants with ten or more third-degree relatives, deviations of mean heterozygosity, sex mismatches, non-white European ancestry, incomplete genetic data, diagnosis of DSCs prior to enrollment (to minimize the spurious causal effects), and diagnosed with liver diseases (to minimize the effects of liver disease on the variation of signature lipidomic biomarkers). Finally, 319,568 participants were identified for this study.

### Assessment of phenotypic data

In this study, measurements of HDL-C, LDL-C, and TG concentrations were implemented with Enzyme immune-inhibition, Enzymatic selective protection, and GPO-POD in AU5800 supplied from Beckman Coulter (Supplementary methods: Assessment of phenotypic data), ranging in serum concentrations from 0.228 to 4.401 mmol/L, from 0.546 to 9.797 mmol/L, and from 0.231 to 11.278 mmol/L. The diagnoses of DSCs, including oesophagus, stomach, colorectal, liver, gallbladder, and pancreas cancers, were coded in accordance with the International Classification of Disease version 10 (ICD-10) (Supplementary methods: Assessment of phenotypic data). All phenotypic data of covariates were obtained from local NHS Primary Care Trust registries, touchscreen questionnaire at Assessment Centre and hospital inpatient records, covering baseline characteristics (age, sex, and Townsend deprivation index (TDI)), sociodemographics (education qualification and employment status), body size measures (BMI), blood biochemistry biomarkers (alanine aminotransferase (ALT), aspartate aminotransferase (AST), and HbA1c (glycated haemoglobin)), physical measures (systolic blood pressure (SBP) and diastolic blood pressure (DBP)), lifestyles (smoking status, alcohol drinking status, and physical activity level), family history (family cancer history), blood sample collection (fasting time), medications (female: medication for cholesterol, blood pressure, diabetes, or take exogenous hormones; male: medication for cholesterol, blood pressure or diabetes), and health-related outcomes (diabetes, cerebral infarction, ischaemic heart disease, and primary hypertension) (Supplementary methods: Assessment of phenotypic data).

### Calculation of polygenic risk scores of signature lipidomic biomarkers

Individual-level phenotypic and genotype data of signature lipidomic biomarkers based on European-ancestry UKB samples were used as target data (Supplementary methods: Quality control for genotype data). In order to avoid substantial inflation of polygenic risk score (PRS)-phenotype relationship due to sample overlap between target data and base data [16], summary-level European-ancestry meta-analysis GWAS datasets of HDL-C, LDL-C, and TG (*N* = 0.9m, EUR) without UKB samples were retrieved from The Global Lipids Genetics Consortium (GLGC) (Table S1) [17]. We firstly applied rigorous quality control procedures to extract independent genetic instrumental variables (GIVs) to be used to calculate HDL-C polygenic risk score (HDL-C-PRS), LDL-C polygenic risk score (LDL-C-PRS), and TG polygenic risk score (TG-PRS): (1) SNPs of GWAS summary datasets in autosomes (MAF > 0.01) for allele mismatches, duplicates, and ambiguous SNPs, without genome-wide significance threshold (*P* ≥ 5E-08) were excluded; (2) SNPs within agreement with linkage disequilibrium (LD) *r*^2^<0.001, Kb<10000 were extracted; (3) potential correlated and horizontal pleiotropies of SNPs with confounders and outcomes were genome-wide significantly (*P*<5E-08) associated within PhenoScanncer (http://www.phenoscanner.medschl.cam.ac.uk/) were excluded [18]; (4) conjoint pleiotropic SNPs that genome-wide significantly (*P* < 5E-08) associated with HDL-C, LDL-C, or TG were excluded. HDL-C-, LDL-C-, and TG-PRS were calculated by multiplying the number of effect alleles by the beta of the corresponding GWAS association value for each GIV [19].

### Phenotypic association analyses

In this study, multivariable-adjusted covariates (age, sex, BMI, smoking status, alcohol drinking status, education qualification, employment status, TDI, physical activity level, family cancer history, ALT, AST, SBP, DBP, cerebral infarction, ischaemic heart disease, primary hypertension, HbA1c, and diabetes) were fitted in the primary analyses. Firstly, we derived the hazard ratios (HRs) and 95% confidence intervals (CIs) of signature lipidomic biomarkers on the risk of DSCs using the Cox proportional hazards regression model, for which the Schoenfeld residuals method was used to evaluate the proportional hazards assumption [20]. We further represented restricted cubic splines (RCSs) to estimate nonlinear associations between signature lipidomic biomarkers and the risk of DSCs with three knots at 10^th^, 50^th^, and 90^th^ percentile of signature lipidomic biomarkers. Moreover, whether estimates of signature lipidomic biomarkers on the risk of DSCs were intervened by age- and sex-specified characteristics was examined with subgroup analyses by subdivisions of signature lipidomic biomarkers (HDL-C: <1.0 mmol/L, 1.0-1.6 mmol/L, ≥1.6 mmol/L; LDL-C: <3.4 mmol/L, 3.4-4.1 mmol/L, ≥4.1 mmol/L; TG: <1.7 mmol/L, 1.7-2.2 mmol/L, ≥2.2 mmol/L) with reference of MOH Clinical Practice Guidelines 12/2016 [21] and the results of RCSs. During sensitivity analyses, we respectively excluded participants within the first two-year follow-up time, additionally adjusting for additional medication use, adjusting for other signature lipidomic biomarkers, as well as adjusting for fasting time to verify the reliability of results of the primary analyses.

### Linear and nonlinear Mendelian randomization analyses

With the evidences of linear or nonlinear phenotypic associations between signature lipidomic biomarkers and the risk of DSCs, we supposed that nonlinear MR analyses causally assessed the nonlinear phenotypic associations, otherwise, linear MR analyses were primarily used to assess the potential linear causal associations for linear or nonlinear phenotypic associations. For nonlinear MR analyses, to avoid overadjustment and collider bias [22], participants were stratified into three subgroups by residual of signature lipidomic biomarkers (the differences between signature lipidomic biomarkers and fitted values of genetically-predicted signature lipidomic biomarkers obtained by regressing signature lipidomic biomarkers on the signature lipidomic biomarker-PRS with multiple linear regression model). Then, piecewise linear method was utilized to estimate causal associations between signature lipidomic biomarkers and the risk of DSCs in each stratum (local average causal effects, LACE), whose nonlinearity was assessed with Quadratic test and the Cochran Q test [12]. Linear one-sample MR analyses using ratio of coefficients method were conducted by regressing signature lipidomic biomarker concentrations on the signature lipidomic biomarker-PRS with multiple linear regression models to calculate the *beta*_*exposure*_ and *se*_*exposure*_, and then by regressing DSC outcomes on the signature lipidomic biomarker-PRS with Cox proportional hazards regression models to calculate *beta*_*outcome*_ and *se*_*outcome*_. *beta*_*MR*_ was obtained as the ratio *beta*_*outcome*_ / *beta*_*exposure*_, and *se*_*MR*_ as *se*_*outcome*_ / *beta*_*exposure*_. Both exposure and outcome regression stages were adjusted for age, sex, assessment centers, genotyping array, and the first 10 principal components (PCs). Several sensitivity analyses were used to validate the robustness of causal associations between signature lipidomic biomarkers and the risk of DSCs. (1) We re-conducted linear MR analyses adjusted for potential confounders in both exposure and outcome regression stages with ratio of coefficients method to prevent the breaching of three key assumptions of MR analyses (Figure S1). (2) Outcome stages in linear MR analyses were re-conducted with logistic regression analyses. From summary-level European-ancestry meta-analysis GWAS datasets without containing UKB samples in GLGC, we identified GIVs after rigorous quality controls to perform linear MR analyses using SNP-based two-sample Mendelian randomization (TSMR) analyses, where inverse-variance weighted (IVW) analyses [23] were used as the primary SNP-based TSMR methods, and weighted median [23], MR-Egger [24], and MR-PRESSO [25] were also conducted as the sensitivity analyses of SNP-based TSMR analyses (Supplementary method: SNP-based MR analyses). In addition, age- and sex-specific stratified MR analyses were conducted to quantify LACEs within the residuals of above three categories of signature lipidomic biomarkers.

### Statistical methods

All phenotypic association analyses were conducted with R (version 4.2.0). Calculation of signature lipidomic biomarker-PRS was completed with PRSice-2 software of Linux version [26]. Multiple linear regression analyses, binary logistic regression analysis, ordinal logistic regression analysis, and multinominal logistic regression were respectively conducted with “stats package”, “rms package”, “MASS package” and “nnet package” of R software to evaluate the associations between signature lipidomic biomarker-PRS and covariates. SNP-based MR analyses were performed with “TwoSampleMR package” and “MRPRESSO package” of R software, and nonlinear MR analyses was employed with “nlmr package” of R software. *F* statistic (*F* statistic = ((*n*-*k*-1)/*k*)(*R*^*2*^/(1-*R*^*2*^), in which *n* means the sample size of the study, *k* represented number of SNPs, and *R*^*2*^ refers to the variability explanation of SNPs) was used to evaluate statistical power of GIVs for signature lipidomic biomarkers. Statistical power of signature lipidomic biomarkers in relation to different DSC outcomes in Mendelian randomization analyses were calculated with online tool (https://sb452.shinyapps.io/power/) [27] (Table S2). On the purpose of avoiding the inflation of type I error, we used the Bonferroni-adjusted *P* values for multiple-test comparisons across our primary analyses, defining *P* value of 0.008 (0.05/6 DSC outcomes) as the threshold for remarkable statistical significance, and *P* value between 0.008 to 0.05 as the suggestive statistical significance.

## Results

### Demographic characteristics

Table 1 shows statistical comparisons of signature lipidomic biomarkers and demographic characteristics between participants with and without DSCs. Median HDL-C and LDL-C concentrations were significantly lower in participants with DSCs than in those without DSCs (1.358 vs. 1.410 mmol/L, 3.508 vs. 3.536 mmol/L, *P*_trend_ < 0.001), whereas a significant opposite tendency was observed for median TG concentration (1.621 vs. 1.478 mmol/L, *P*_trend_ < 0.001), age (62.000 vs. 58.000 years, *P*_trend_ = 0.000), BMI (27.367 vs. 26.638 kg/m^2^, *P*_trend_ < 0.001), TDI (-2.283 vs. -2.305,* P*_trend_ = 0.023), ALT (24.900 vs. 24.300 U/L, *P*_trend_ < 0.001), AST (20.740 vs. 19.990 U/L, *P*_trend_ < 0.001), SBP (143.000 vs. 138.000 mmHg, *P*_trend_ < 0.001), DBP (83.000 vs. 82.000 mmHg, *P*_trend_ < 0.001), and HbA1c (36.050 vs. 35.100 mmol/mol, *P*_trend_ < 0.001). Meanwhile, DSCs were more common in male participants, previous or current smokers, never or previous alcohol drinkers, none-employed participants, and participants taking given medications, lacking physical activity, as well as with cerebral infarction, ischaemic heart disease, primary hypertension, diabetes, and family cancer history.

**Table 1:** Demographic characteristics of all participants with and without DSCs

**Characteristics**	**All participants** **(*N*=319,568)**	**Without digestive system cancers** **(*N*=312,652)**	**Digestive system cancers**^**a**^** (*N*=6916)**	***P***_**trend**_
**HDL-C (mmol/L)**	1.409 [1.181;1.685]	1.410 [1.182;1.686]	1.358 [1.135;1.635]	<0.001
**LDL-C (mmol/L)**	3.536 [2.965;4.135]	3.536 [2.967;4.136]	3.508 [2.888;4.111]	<0.001
**TG (mmol/L)**	1.481 [1.046;2.138]	1.478 [1.044;2.134]	1.621 [1.140;2.321]	<0.001
**Age (years)**	58 [50;63]	58 [50;63]	62 [58;66]	0.000
**Sex, n (%)**				<0.001
Female	173,073 (54.158%)	170,089 (54.402%)	2984 (43.146%)	
Male	146495 (45.842%)	142563 (45.598%)	3932 (56.854%)	
**BMI (kg/m**^**2**^**)**	26.655 [24.089;29.750]	26.638 [24.076;29.728]	27.367 [24.794;30.504]	<0.001
**Smoking status, n (%)**				<0.001
Never	173,465 (54.281%)	170,425 (54.509%)	3040 (43.956%)	
Previous	113,615 (35.553%)	110,571 (35.366%)	3044 (44.014%)	
Current	32,488 (10.166%)	31,656 (10.125%)	832 (12.030%)	
**Alcohol drinking status, n (%)**				0.002
Never	10,036 (3.140%)	9801 (3.135%)	235 (3.398%)	
Previous	10,494 (3.284%)	10,219 (3.268%)	275 (3.976%)	
Current	299,038 (93.576%)	292,632 (93.597%)	6406 (92.626%)	
**Education qualification, n (%)**				0.262
None	55,338 (17.317%)	54,142 (17.317%)	1196 (17.293%)	
NVQ/CSE/O/A levels	143,063 (44.768%)	140,027 (44.787%)	3036 (43.898%)	
Professional/college or university degree	121,167 (37.916%)	118,483 (37.896%)	2684 (38.809%)	
**Employment status, n (%)**				<0.001
None employed	134,924 (42.221%)	130,912 (41.871%)	4012 (58.010%)	
Current employed	184,644 (57.779%)	181,740 (58.129%)	2904 (41.990%)	
**Medication use**^**b**^**, n (%)**				<0.001
None	219,136 (68.573%)	214,976 (68.759%)	4160 (60.150%)	
Yes	100,432 (31.427%)	97,676 (31.241%)	2756 (39.850%)	
**TDI**	-2.305 [-3.710;0.134]	-2.305 [-3.711;0.131]	-2.283 [-3.669;0.348]	0.023
**Physical activity level, n (%)**				0.066
None	18,014 (5.637%)	17,580 (5.623%)	434 (6.275%)	
Light/Moderate activity	252,040 (78.869%)	246,619 (78.880%)	5421 (78.383%)	
Heavy/Strenuous activity	49,514 (15.494%)	48,453 (15.497%)	1061 (15.341%)	
**Family cancer history, n (%)**				<0.001
None	219,398 (68.655%)	214,870 (68.725%)	4528 (65.471%)	
Yes	100,170 (31.345%)	97,782 (31.275%)	2388 (34.529%)	
**Fasting time (hours)**	4 [4;5]	4 [4;5]	4 [4;5]	1.000
**ALT (U/L)**	24.300 [21.000;28.600]	24.300 [21.000;28.600]	24.900 [21.400;29.200]	<0.001
**AST (U/L)**	20.000 [15.340;27.090]	19.990 [15.330;27.070]	20.740 [15.818;27.490]	<0.001
**SBP (mmHg)**	138 [126;152]	138 [126;152]	143 [130;156]	<0.001
**DBP (mmHg)**	82 [75;89]	82 [75;89]	83 [76;90]	<0.001
**Cerebral infarction, n (%)**				<0.001
None	312,384 (97.752%)	305,740 (97.789%)	6644 (96.067%)	
Yes	7184 (2.248%)	6912 (2.211%)	272 (3.933%)	
**Ischaemic heart disease, n (%)**				<0.001
None	289,703 (90.655%)	283,787 (90.768%)	5916 (85.541%)	
Yes	29,865 (9.345%)	28,865 (9.232%)	1000 (14.459%)	
**Primary hypertension, n (%)**				<0.001
None	226,576 (70.901%)	222,979 (71.319%)	3597 (52.010%)	
Yes	92,992 (29.099%)	89,673 (28.681%)	3319 (47.990%)	
**HbA1c (mmol/mol)**	35.100 [32.700;37.700]	35.100 [32.700;37.600]	36.050 [33.500;38.800]	<0.001
**Diabetes, n (%)**				<0.001
None	296,342 (92.732%)	29,0557 (92.933%)	5785 (83.647%)	
Yes	23,226 (7.268%)	22,095 (7.067%)	1131 (16.353%)	

The values of continuous variables (HDL-C, LDL-C, TG, age, BMI, TDI, fasting time, ALT, AST, SBP, and DBP) were presented as medians [upper quartiles; lower quartiles], and the categorial variables (sex, smoking status, alcohol drinking status, education qualification, employment status, medication use, physical activity level, family cancer history, cerebral infarction, ischaemic heart disease, primary hypertension, and diabetes) were displayed as n (%)

*BMI* Body mass index, *TDI* Townsend deprivation index, *ALT* Alanine aminotransferase, *AST* Aspartate aminotransferase, *SBP* Systolic blood pressure, *DBP* Diastolic blood pressure, *HbA1c* Glycated haemoglobin, *HDL-C* High-density lipoprotein cholesterol, *LDL-C* Low-density lipoprotein cholesterol, *TG* Triglyceride

^a^Participants with DSCs including oesophagus, stomach, colorectal, liver, gallbladder and pancreas cancers were included in this study

^b^Medication use including cholesterol lowering medication (female and male), blood pressure medication (female and male), insulin (female and male), hormone replacement therapy (female only) or oral contraceptive pill or minipill (female only)

### Phenotypic results between signature lipidomic biomarkers and the risk of digestive system cancers

**Fig. 2:**
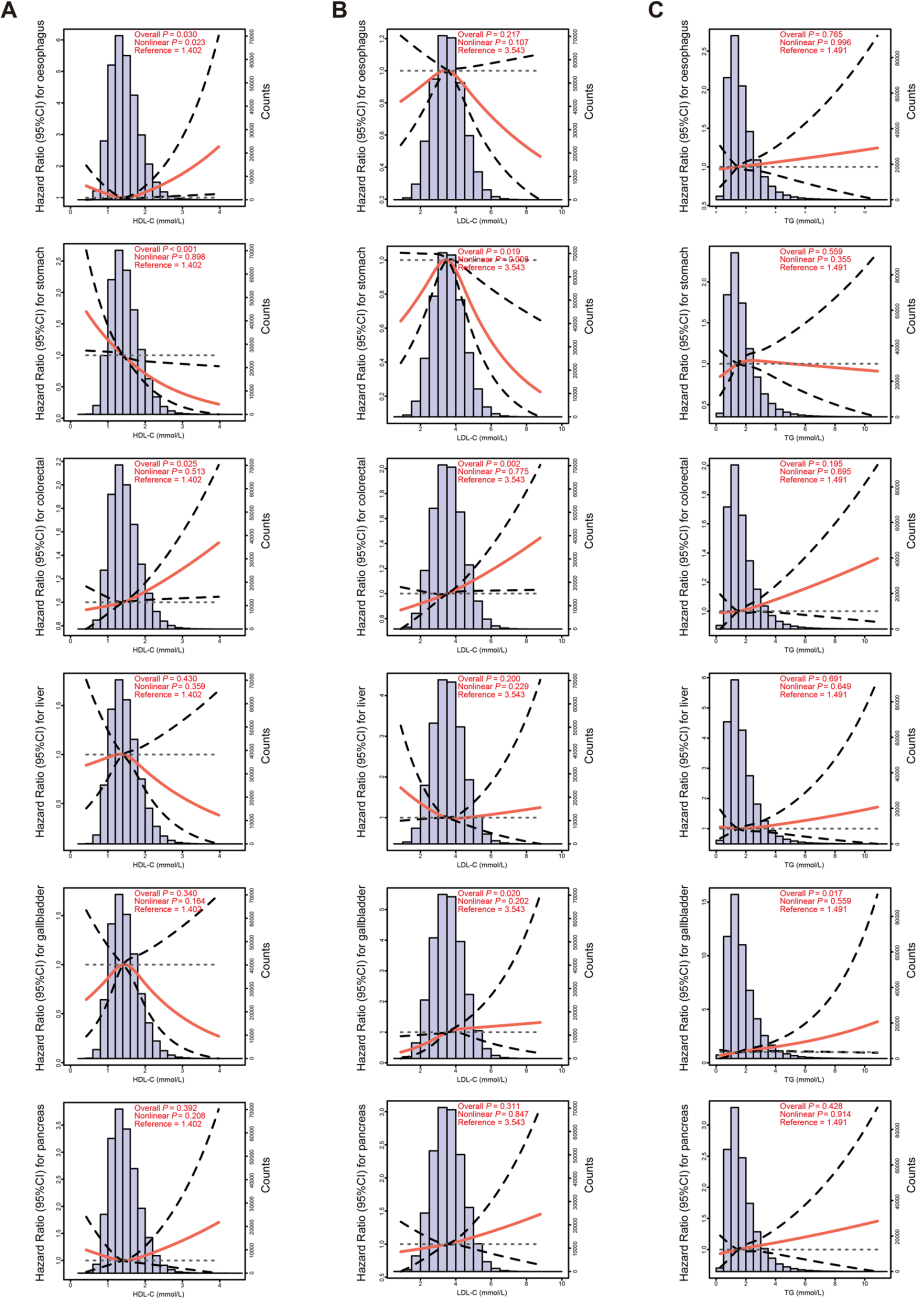
Phenotypic association patterns between signature lipidomic biomarkers (HDL-C (**A**), LDL-C (**B**), and TG (**C**)) and the risk of DSC. Distributions of signature lipidomic biomarker concentrations and RCSs (red lines) representing shapes of phenotypic associations with adjustment of age, sex, BMI, smoking status, alcohol drinking status, education qualification, employment status, TDI, physical activity level, medication use, family cancer history, ALT, AST, SBP, DBP, primary hypertension, cerebral infarction, ischaemic heart disease, HbA1c, and diabetes. *P*_overall_ and *P*_nonlinear_ values of 0.008 (0.05/6 outcomes, Bonferroni-adjusted *P*) were defined as the threshold of remarkable statistical significance.* P*_overall_ and *P*_nonlinear_ values between 0.008 and 0.05 were defined as suggestive statistical significance

**Fig. 3:**
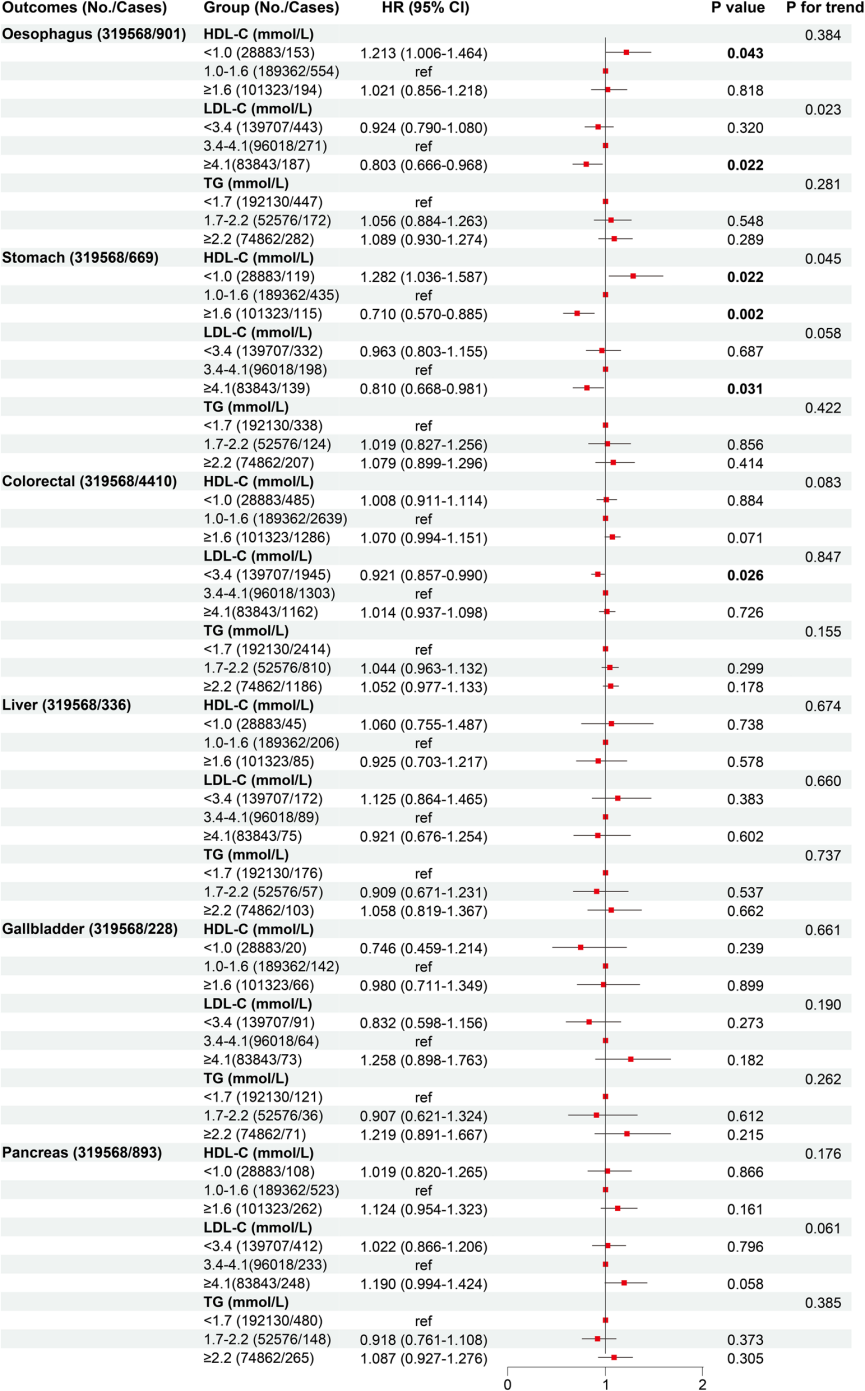
Phenotypic associations between three categories of signature lipidomic biomarkers and the risk of DSCs. Hazard ratios (HRs) (red squares) with 95% CIs (black solid lines) of three categories of HDL-C, LDL-C, and TG concentrations on the risk of oesophagus, stomach, colorectal, liver, gallbladder, and pancreas cancers with adjustment of age, sex, BMI, smoking status, alcohol drinking status, education qualification, employment status, TDI, physical activity level, medication use and family cancer history, ALT, AST, SBP, DBP, primary hypertension, cerebral infarction, ischaemic heart disease, and diabetes. *P* and *P*_trend_ value of 0.008 (0.05/6 ourcomes, Bonferroni-adjusted *P*) were defined as the threshold for remarkable statistical significance. *P* and *P*_trend_ values of 0.05 were defined as suggestive statistical significance

In general, as shown in Fig. 2, a remarkable independent negative association was observed between HDL-C concentration and the risk of stomach cancer (*P*_nonlinear_ = 0.898, *P*_overall_ < 0.001), and suggestive linear positive associations were observed between HDL-C concentration and the risk of colorectal cancer (*P*_nonlinear_ = 0.513, *P*_overall_ = 0.025), and between TG concentration and the risk of gallbladder cancer (*P*_nonlinear_ = 0.559, *P*_overall_ = 0.017). A suggestive independent nonlinear association between LDL-C concentration and the risk of stomach cancer (upside down J-shaped: *P*_nonlinear_ = 0.008, *P*_overall_ = 0.019) was observed. Sensitivity analyses still demonstrated robust results after excluding participants within the first two-year follow-up time (Figure S2), additionally adjusting for additional medication use (Figure S3), adjusting for fasting time (Figure S4), and adjusting for other signature lipidomic biomarkers concentrations (Figure S5). Though we observed a suggestive nonlinear association between HDL-C concentration and the risk of oesophagus cancer (*P*_nonlinear_ = 0.023, *P*_overall_ = 0.030), and a remarkable linear positive association between LDL-C concentration and the risk of colorectal cancer (*P*_nonlinear_ = 0.775, *P*_overall_ = 0.002), above phenotypic evidences became less pronounced in sensitivity analyses (HDL-C- oesophagus cancer: Figures S4 and S5; LDL-C- colorectal cancer: Figure S3). When divided with reference to MOH Clinical Practice Guidelines 12/2016 [21] and the results of RCSs, we re-evaluated the risk of DSCs among participants in different categories of signature lipidomic biomarkers (Fig. 3). As compared to the category of HDL-C 1.0-1.6 mmol/L, the category of HDL-C<1.0 mmol/L was respectively suggestively associated with approximately 20% and 30% higher risk of oesophagus (HR: 1.213 (1.006-1.464), *P*=0.019) and stomach (HR: 1.282 (1.036-1.587), *P*=0.022) cancers, whereas the category of HDL-C ≥ 1.6 mmol/L was remarkably associated with approximately 30% lower risk of stomach cancer (HR: 0.710 (0.570-0.885), *P*=0.002). Moreover, compared those with the category of LDL-C 3.4-4.1 mmol/L, suggestive evidence indicated that about 20% lower risk of oesophagus (HR: 0.803 (0.666-0.968), *P*=0.022) and stomach (HR: 0.810 (0.668-0.981), *P*=0.031) cancers were observed among participants with the category of LDL-C ≥ 4.1 mmol/L.

### Genetic estimates of signature lipidomic biomarkers

Totally, 122-GIV (Table S3) HDL-C-PRS (*F*-statistic=55.917), 106-GIV (Table S4) LDL-C-PRS (*F*-statistic = 64.192), and 81-GIV (Table S5) TG-PRS (*F*-statistic = 50.284) respectively explained 3.0%, 2.8%, and 2.2% of variance in HDL-C, LDL-C, and TG concentrations from individual-level European-ancestry data in the UKB. Suggestive genetic evidences indicated that the prevalence of stomach cancer was negative associated with LDL-C-PRS quartiles (*P*_trend_ = 0.014) (Table S7). However, there were no statistically significant associations between the prevalence of other DSC outcomes and PRS of signature lipidomic biomarkers (*P*_trend_ ≥ 0.05) (Tables S6-S8). Moreover, we also observed that HDL-C-PRSs were remarkably negatively associated with the risk of ischaemic heart disease and primary hypertension (*P* < 2.50E-03), and LDL-C-PRSs were remarkably positively associated with the risk of ischaemic heart disease and HbA1c (*P* < 2.50E-03), and TG-PRSs were remarkably positively associated with the SBP, DBP, HbA1c, the risk of ischaemic heart disease, primary hypertension, and diabetes (*P* < 2.50E-03) (Table S9). In addition, there were suggestive evidences that HDL-C-PRSs negatively associated with SBP and diabetes (Table S9).

### Comparisons between phenotypic and Mendelian randomization analyses

**Fig. 4:**
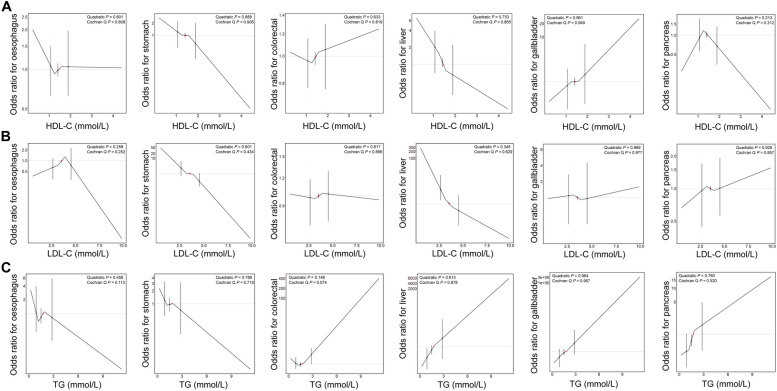
Causal association patterns between signature lipidomic biomarkers and the risk of DSCs. Nonlinear MR analyses with piecewise linear method for genetically predicting the associations between (**A**) HDL-C, (**B**) LDL-C, and (**C**) TG concentrations and the risk of oesophagus, stomach, colorectal, liver, gallbladder, and pancreas cancers. Exposure and outcome regression stages were both adjusted with age, sex, assessment centers, genotyping array and the first 10 PCs. Each black dot and black vertical line represented the LACE with its 95% confidence interval in each stratum and red dots represented reference points

**Fig. 5:**
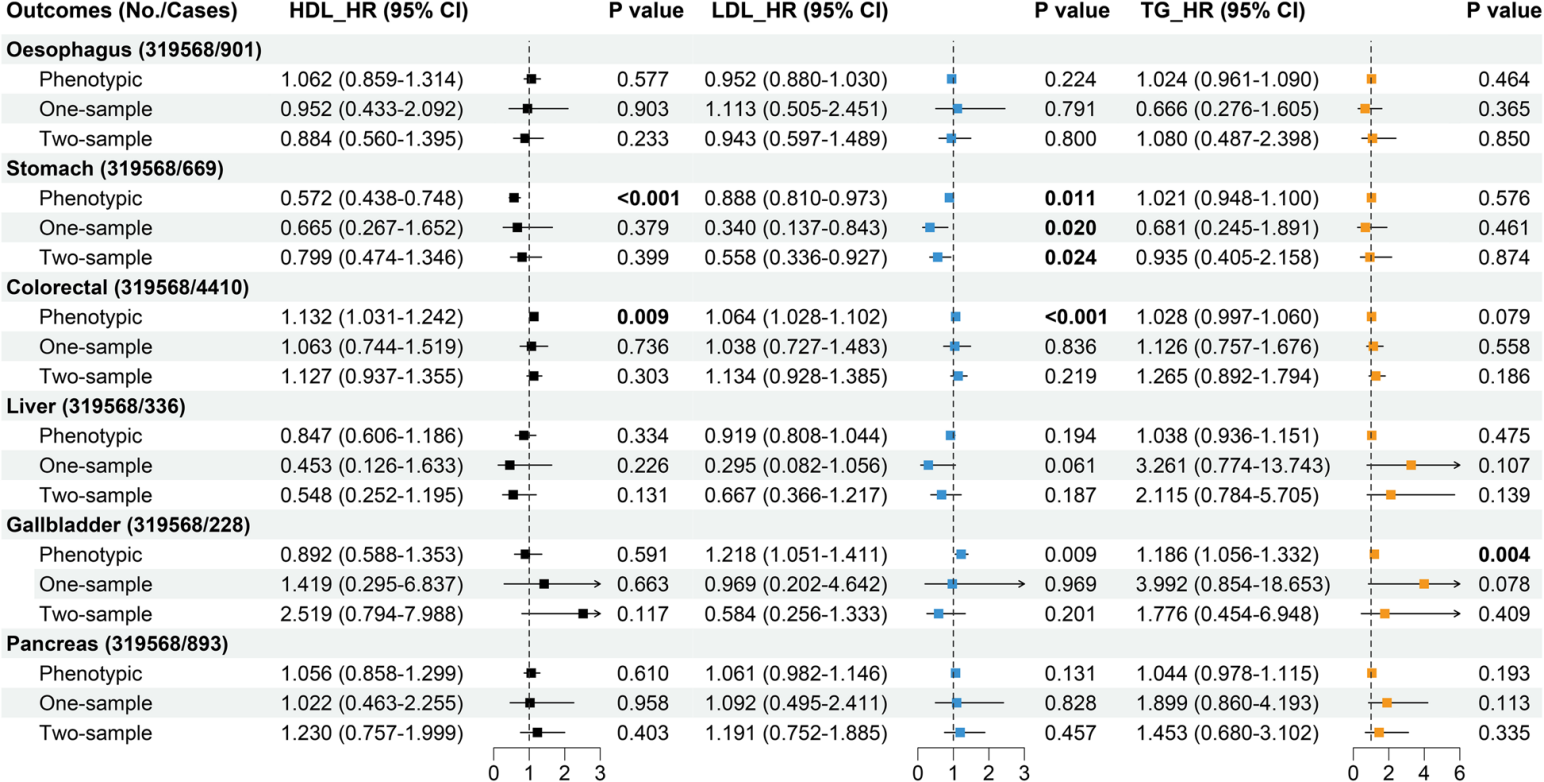
Phenotypic and genetic association analyses of signature lipidomic biomarkers with the risk of DSCs. Phenotypic association analyses were adjusted with age, sex, BMI, smoking status, alcohol drinking status, education qualification, employment status, TDI, physical activity level, medication use, family cancer history, ALT, AST, SBP, DBP, primary hypertension, cerebral infarction, ischaemic heart disease, HbA1c, and diabetes. Linear one- and two-sample MR analyses were both adjusted with age, sex, assessment centers, genotyping array and the first 10 PCs. HRs (HDL-C: black squares, LDL-C: blue squares; TG: orange squares) with 95% Cis for oesophagus, stomach, colorectal, liver, gallbladder, and pancreas cancers per 1 mmol/L increase of HDL-C, LDL-C, and TG concentrations. *P* value of 0.008 (0.05/6 outcomes, Bonferroni-adjusted *P*) was defined as the threshold for remarkable statistical significance. *P* value between 0.008 and 0.05 was defined as suggestive statistical significance

Though phenotypic association patterns characterized with RCSs represented suggestive nonlinear associations between HDL-C concentration and the risk of oesophagus cancer, and between LDL-C concentration and the risk of stomach cancer, no remarkable or suggestive evidences suggested nonlinear causal associations between signature lipidomic biomarkers and the risk of DSCs in nonlinear MR analyses by calculating LACEs in three-quantile strata of participants based on residual signature lipidomic biomarkers (Fig. 4), even in sensitivity analyses (Fig. S6). Meanwhile, suggestive phenotypic evidence exhibited that LDL-C concentration showed a negative association with the risk of stomach cancer (HR: 0.888 (0.810-0.973), *P* = 0.011) (Fig. 5). A similar trend was also found in the corresponding causal association result in linear one- and two-sample MR analyses respectively using ratio of coefficient method (HR: 0.340 (0.137-0.843), *P* = 0.020)) and IVW method (OR: 0.558 (0.336-0.927), *P* = 0.024)) (Fig. 5). As was shown in Table S10, sensitivity analyses of linear one-sample MR analyses with additional adjustments of potential confounders (HR: 0.343 (0.142-2.830), *P* = 0.018)) and outcome stage using logistic regression model (HR: 0.387 (0.176-0.852), *P*=0.018)), and other linear two-sample MR methods (MR-Egger (OR: 0.373 (0.162-0.854), *P* = 0.022), and MR-PRESSO (OR: 0.558 (0.343-0.910), *P* = 0.021) methods) remained consistent with the findings of primary linear one- and two-sample MR analyses of LDL-C concentration with the risk of stomach cancer. Moreover, though remarkable phenotypic evidences were identified between HDL-C concentration and the risk of stomach cancer (HR: 0.572 (0.438-0.748), *P* < 0.001), and between LDL-C concentration and the risk of colorectal cancer (HR: 1.064 (1.028-1.102), *P* < 0.001), and between TG concentration and the risk of gallbladder cancer (HR: 1.186 (1.056-1.332), *P* = 0.004) (Fig. 5), linear one- and two-sample MR analyses did not support genetic causal associations among them, whose corresponding causal HRs using ratio of coefficient methods were 0.665 (0.267-1.652), 1.038 (0.727-1.483), and 3.992 (0.854-18.653), and corresponding causal ORs using IVW methods were 0.799 (0.474-1.346), 1.134 (0.928-1.385), and 1.776 (0.454-6.948) (Fig. 5).

### Age- and sex-subgroup analyses and stratified MR analyses

In age-specific subgroup analyses (Tables S11-S13), HDL-C concentration had remarkable interactions with age on the risk of oesophagus cancer (*P*_interaction_ = 0.006), and LDL-C concentration suggestively and remarkably interacted with age on the risk of colorectal (*P*_interaction_ = 0.012) and liver cancers (*P*_interaction_ < 0.001), respectively. For participants aged less than 60 years, the category of HDL-C ≥ 1.6 mmol/L was suggestively positively associated with the risk of colorectal cancer (HR: 1.138 (1.004-1.290), *P* = 0.043) as compared to the category of HDL-C 1.0-1.6 mmol/L, and the category of LDL-C ≥ 4.1 and LDL-C < 3.4 mmol/L were respectively remarkably (HR: 0.623 (0.452-0.858), *P* = 0.004) and suggestively (HR: 0.762 (0.585-0.992), *P* = 0.044) negatively associated with risk of oesophagus cancer as compared to the category of LDL-C 3.4-4.1 mmol/L. Meanwhile, for participants aged 60 years or older, the categories of HDL-C < 1.0 and ≥ 1.6 mmol/L were remarkably associated with higher (HR: 1.424 (1.107-1.833), *P* = 0.006) and lower (HR: 0.683 (0.523-0.892), *P* = 0.005) risks of stomach cancer as compared to the category of HDL-C 1.0-1.6 mmol/L. Moreover, participants aged 60 years or older with the category of LDL-C < 3.4 mmol/L had suggestive negative associations with the risk of colorectal cancer (HR: 0.910 (0.832-0.996), *P* = 0.041), and the category of LDL-C ≥ 4.1 mmol/L had suggestively higher risk of gallbladder (HR: 1.541 (1.020-2.328), *P* = 0.040) and pancreas (HR: 1.341 (1.079-1.665)), *P* = 0.008) cancers as compared to those with the category of LDL-C 3.4-4.1 mmol/L, and those with the category of TG ≥ 2.2 mmol/L had suggestively higher risk of gallbladder cancer (HR: 1.331 (1.009-1.757), *P* = 0.043) as compared to those with the category of TG < 1.7 mmol/L. Furthermore, integrating linear (Table S14) and nonlinear MR analyses (Figures S7-S8) indicated that there was no nonlinear causal pattern, but an overall estimation that per increase of 1 mmol/L in LDL-C concentration remarkably causally decreased the risk of stomach cancer by almost 74% (HR: 0.264 (0.103-0.677), *P* = 0.006) in participants aged 60 years or older. LACEs calculated from stratified MR analyses (Tables S15-S17) suggested that the category of LDL-C ≥ 4.1 mmol/L showed a more suggestive inverse causal association with the risk of stomach cancer (HR: 0.786 (0.638-0.969), *P* = 0.024) (Table S16) as compared to the categories of LDL-C < 4.1 or 3.4-4.1 mmol/L. Meanwhile, though we did not detect an overall linear or nonlinear causal pattern between TG concentration and the risk of gallbladder cancer, the category of TG ≥ 2.2 mmol/L suggestively causally increased the risk of gallbladder cancer among participants aged 60 years or older (HR: 1.264 (1.003-1.593), *P* = 0.047).

In sex-specific subgroup analyses (Tables S11-S13), a suggestive interaction was found between TG concentration and sex on the risk of liver cancer (*P*_interaction_ = 0.011). For female participants, the category of HDL-C < 1.6 mmol/L had a suggestively positive association with the risk of stomach cancer (HR: 2.062 (1.181-3.063), *P* = 0.011) as compared to the category of HDL-C 1.0-1.6 mmol/L, and the category of LDL-C ≥ 4.1 mmol/L was suggestively associated with a lower risk of stomach cancer (HR: 0.726 (0.548-0.960), *P* = 0.025) as compared to the category of LDL-C 3.4-4.1 mmol/L. For male participants, the category of HDL-C ≥ 1.6 mmol/L was remarkably negatively associated with the risk of stomach cancer (HR: 0.576 (0.409-0.810), *P* = 0.002) as compared to the category of HDL-C 1.0-1.6 mmol/L, and the category of LDL-C ≥ 4.1 mmol/L showed a remarkably negative association with the risk of oesophagus cancer (HR: 0.688 (0.544-0.869), *P* = 0.002), and a suggestively negative association with the risk of gallbladder cancer (HR: 1.761 (1.027-3.019), *P* = 0.040) as compared to the category of LDL-C 3.4-4.1 mmol/L. Moreover, male participants with the category of TG ≥ 2.2 mmol/L had suggestively higher risk of gallbladder cancer (HR: 1.589 (1.017-2.482), *P* = 0.042) as compared to those with the category of TG < 1.7 mmol/L. Linear (Table S14) and nonlinear (Figure S9-S10) MR analyses revealed a suggestive negative causal association (HR: 0.601 (0.396-0.913), *P* = 0.017) without a nonlinear pattern between LDL-C concentration and the risk of stomach cancer among female participants, especially those within the category of LDL-C ≥ 4.1 mmol/L (HR: 0.789 (0.637-0.977), *P* = 0.030) (Table S16). Though unclear linear or nonlinear MR evidences for overall causal effects of TG concentration on the risk of gallbladder cancer, the suggestively injurious causal effect of the category of TG ≥ 2.2 mmol/L on the higher risk of gallbladder cancer was unequivocal in male participants (HR: 1.447 (1.020-2.052), *P* = 0.038).

## Discussion

Increasing evidences suggested that serum lipid dysfunctions might contribute to carcinogenesis of DSCs, yet this association remained ambiguous. In this study, based on the UKB prospective cohort, we determined that phenotypic and genetic evidences confirmed the overall inverse association between LDL-C concentration and the risk of stomach cancer. More significant age- and sex-specific effects were observed between the category of LDL-C ≥ 4.1 mmol/L and the risk of stomach cancer. The negative causal effects of the category of LDL-C ≥ 4.1 mmol/L on the risk of stomach cancer were stronger in female participants and participants aged 60 years or older as compared to the category of LDL-C < 3.4 or 3.4-4.1 mmol/L. Moreover, the category of TG ≥ 2.2 mmol/L increased the risk of gallbladder cancer among male participants and participants aged 60 years or older though no significant overall causal association between TG concentration and the risk of gallbladder. In addition, phenotypic analyses had shown that HDL-C concentration was associated with the risk of oesophagus cancer in a nonlinear pattern, and was negatively associated with the risk of stomach cancer in a linear pattern, but there were no MR-based evidences to support their causal associations.

Though there are evidences that reduced serum lipids are related to the courses of specific disease and might exacerbate carcinogenesis. For instance, HDL-C and LDL-C concentrations will decrease with the increased severity of liver disease as the results of the reduction of liver synthesis ability [28, 29]. In this regard, patients with asymptomatic liver disease accompanied by low serum lipids due to the reduction in liver synthesis ability, are more susceptible to develop liver sclerosis and even liver cancer [30]. Therefore, in this study, to exclude potential interferences of confounders or possible reverse causalities, we pre-excluded participants diagnosed with liver diseases, and fully evaluated and eliminated interferences of confounders including cardiovascular disease status and diabetes to conclude robust phenotypic findings. Moreover, our study combined phenotypic association evidences with genetic association evidences to improve frailty with only phenotypic association evidence and explore the genuine associations between signature lipidomic biomarkers and the risk of DSCs [31–33]. Previous traditional observational analyses have preliminarily investigated direct phenotypic associations between serum lipids and the risk of DSCs, whose results showed co-ethnic or even inter-racial consistencies with partial results of our study. Oh et al. recently reported based on a nationwide population-based cohort study that HDL-C concentration was found to be independently negatively correlated and with the risk of stomach cancer (HR: 0.98 (0.96-0.99), *P* < 0.0001) [34]. A meta-analysis showed that HDL-C and TG concentrations were independently correlated with the risk of colorectal cancer in linearly positive and negative manners [9]. TG concentration has been reported as an independent risk factor for gallbladder cancer in Chinese populations [35]. Though our study extended primary observational results to phenotypic pattern evidences, and supported remarkable independent linear associations between HDL-C and the risk of stomach cancer, suggestive independent linear associations between HDL-C and the risk of colorectal cancer, and between TG concentration and the risk of gallbladder cancer, nonlinear and linear MR-based evidences with non-confounding interferences determined no genetic associations between them. Meanwhile, recent observational findings reported that higher LDL-C levels were also associated with lower gastric cancer risk (HR = 0.92 (0.91-0.94), *P* < 0.0001), and untargeted metabolomics analyses also revealed that patients with gastric cancer have a lower LDL-C concentration than normal or gastritis patients with statistical differences (*P* < 0.05) [36]. Corresponding to previous observational findings, a suggestive nonlinear association pattern between LDL-C concentration and the risk of stomach cancer, and the category of LDL-C ≥ 4.1 mmol/L demonstrated a suggestive lower risk of stomach cancer, whose results in phenotypic perspectives did not recurred in genetic perspectives, where the suggestive negative causal associations between LDL-C concentration and the risk of stomach cancer without a nonlinear effect.

In our acknowledgement, this is the first study to investigate the age- and sex-specific effects of signature lipidomic biomarkers on the risk of DSCs based on European population. We concluded that the category of LDL-C ≥ 4.1 mmol/L respectively served as a causal protective role for the risk of stomach cancer among female participants and participants aged 60 years or older, and a detrimental role for the risk of gallbladder cancer among male participants and participants aged 60 years or older with phenotypic and genetic evidences. The association of LDL-C concentration with the risk of stomach cancer seemed to reach inter-racial consistencies. Based on Asia-wide population prospective cohort, Lim et al. reported that a higher LDL-C concentration was inversely associated with the risk of gastric cancer, especially among postmenopausal women (≥ 54 years) [37]. Although molecular interactions between LDL-C and stomach cancer among participants aged 60 years or older have not been ascertained, we could speculate that estrogen benefits female participants by anti-inflammatory properties [38], as well as suppressing proliferation and promoting apoptosis of gastric cancer cells [39]. What’s more, with regard to gallbladder cancer, a previous observational analysis preliminarily revealed that elevated TG concentration was independently positively correlated with the high risk of gallbladder cancer [35], which was consistent with our study. Despite the fact of insufficient MR evidences to support the linear causal effect of TG concentration on the risk of gallbladder cancer, the category of TG ≥ 2.2 mmol/L could be perceived as the robust risk biomarker for male participants and participants aged 60 years or older with sufficient phenotypic and stratified MR evidences.

Concerns regarding the safety of serum lipid concentration variations were also worth contemplating. High HDL-good cholesterol and high LDL-bad cholesterol concentrations have been universally acknowledged in traditional viewpoints. For example, an inter-racial meta-analysis reported that low level of HDL-C concentration and high levels of LDL-C and TC concentrations overall increased the risk of cardiovascular diseases (CVDs) and all-cause mortality [40]. However, paradoxical findings pose challenges to traditional interpretations of serum lipids on the adverse outcomes. Recent cohort study involving approximately 20,000 CAD individuals in the UKB and EmCAB suggested that individuals with CAD who had HDL-C levels above 80 mg/dL had higher mortality rates [41]. A similar range of HDL-C level > 80 mg/dL was also observed to be associated with the increase of cardiovascular risk of male hypertensive individuals [42]. In this study, nonlinear or linear MR-based evidences did not suggest HDL-C concentration causally associated the risk of DSCs, inferring that it was unnecessary to take the risk of DSCs into account when assessing safety concerns related to HDL-C concentration variation. In general, there has been agreement regarding the benefits of a low LDL-C concentration for cardiovascular risk [43–45]. In the FOURIER-OLE cohort, patients with atherosclerotic cardiovascular disease achieving LDL-C levels below 20 mg/dL were monotonically correlated with the lower risk of cardiovascular events [43]. Likewise, similar findings were also observed in the ODYSSEY OUTCOMES Trial, whose findings broadened LDL-C levels below 50 mg/dL with reduction in the risk of major adverse cardiovascular events [44]. A meta-analysis of 170,000 participants in 26 RCTs concluded that reducing LDL-C by 2-3 mmol/L decreased major vascular events by approximately 40-50% [45]. Nevertheless, previous studies have reported that LDL-C concentration variation impacted safety outcomes in more ways than just monotonically increasing the risk of adverse cardiovascular events at high LDL-C concentration [46, 47]. An analysis enrolling 15,281 participants from the IMPROVE-IT Trial, patients with LDL-C concentrations below 30 mg/dL exhibited higher rate of new, worsening, or relapsing malignancies (unadjusted *P*_trend_ = 0.04), even statistical differences slightly waned after accounting for baseline characteristics (adjusted *P*_trend_ = 0.14) [47]. In this study, MR evidences delineated null effects of the categories of LDL-C concentration except for the long-term negative effect of the category of LDL-C ≥ 4.1 mmol/L on the risk of stomach cancer in the given participants, which dispelled the safety concerns of the risk of DSCs caused by extreme low LDL-C concentration.

In our study, there are some strengths that we should highlight. Firstly, it is the first study to systematically examine the independent phenotypic and causal associations between signature lipidomic biomarkers and the risk of DSCs using large-scale prospective cohort of the UKB. Secondly, linear and nonlinear MR analyses for linear and nonlinear phenotypic associations preventing potential damage from RCTs to investigate causal effects of signature lipidomic biomarkers on the risk of DSCs. Thirdly, in light of phenotypic associations between signature lipidomic biomarkers and the risk of DSCs with age- and sex-specific subgroup analyses, we conducted age- and sex-specific stratified MR analyses to further genetically confirmed the causal effect sizes of the categories of signature lipidomic biomarkers among given participants. This study also has some limitations. First of all, although the population stratification effect was minimized as the study was confined to European-ancestry participants and the results of some cross-ethnic phenotypic associations were compatible with our research, further researches on other ethnic groups will provide additional evidence to verify the stable extrapolation effect of our findings. Secondly, the deviation of causal effects caused by horizontal pleiotropy often interferes with the causal effects of exposures on outcomes in MR analysis [48]. We restricted GIVs that were genome-wide significantly associated with signature lipidomic biomarkers, excluded SNPs associated with the risk of DSCs, and replicated the findings using multiple sensitivity analyses and horizontal pleiotropic tests in order to verify their robustness. Thirdly, both linear and nonlinear MR analyses estimated average causal effects. In other words, the causal effects of signature lipidomic biomarkers on the risk of DSCs might differ among individuals. Fourth, the UKB database samples were excluded from the GIV selection and evaluation process to prevent overfitting of association analysis results [16]. Although these GIVs have genome-wide significant associations with signature lipidomic biomarkers, the limited explanation might not fully permit quantification of the associations between signature lipidomic biomarkers and the risk of DSCs.

## Conclusions

In summary, independent complicated association patterns between signature lipidomic biomarkers and the risk of DSCs were confirmed, demonstrating that signature lipidomic biomarkers could serve as biomarkers on the stratification of DSC risk. What’s more, higher LDL-C concentration (≥ 4.1 mmol/L) suggestively benefited female participants and participants aged 60 years or older on the risk of stomach cancer, and male participants and participants aged 60 years or older might be victimized by higher TG concentration (≥ 2.2 mmol/L) on the risk of gallbladder cancer. These findings provided novel references on signature lipidomic biomarkers regulations for public preventions of DSCs.

### Supplementary Information

**Supplementary Material 1.**

## Data Availability

UK Biobank has approved the study and granted access to the data under Application Number 84347. All UK Biobank data can be accessed from the UK Biobank website (www.ukbiobank.ac.uk/) upon request.
