# Supplementary material for: Appraising associations between signature lipidomic biomarkers and digestive system cancer risk: novel evidences from a prospective cohort study of UK Biobank and Mendelian randomization analyses
Source: Lipids Health Dis. 2024 Feb 28;23:61. doi: 10.1186/s12944-024-02053-9 (PMC10900802; doi:10.1186/s12944-024-02053-9)
Supplement: Supplementary file 1 — Supplementary Material 1. [file 12944_2024_2053_MOESM1_ESM.pdf]

Appraising associations between signature lipidomic biomarkers and digestive system cancer risk: novel evidences from a prospective cohort study of UK Biobank and Mendelian randomization analyses

Contents

Supplementary Methods: 3

Assessment of phenotypic data 3

Quality control for genotype data 7

SNP-based MR analyses 8

Reference 8

Supplementary Tables 10

Table S1. Characteristics of European-ancestry cohorts for summary-level GWAS datasets (Excluding UK biobank)* 10

Table S2. Statistical power of signature lipidomic biomarkers in relation to different DSC outcomes in Mendelian randomization analyses (*N* = 319,568). 16

Table S3. SNPs used for calculating HDL-C-PRS 17

Table S4. SNPs used for calculating LDL-C-PRS 20

Table S5. SNPs used for calculating TG-PRS 23

Table S6. Genetic associations between HDL-C-PRS and the risk of digestive system cancer (*N* = 319,568) 25

Table S7. Genetic associations between LDL-C-PRS and the risk of digestive system cancer (*N* = 319,568) 26

Table S8. Genetic associations between TG-PRS and the risk of digestive system cancer (*N* = 319,568) 27

Table S9. Associations between PRSs of signature lipidomic biomarkers and covariates (*N* = 319,568) 28

Table S10. Sensitivity analyses for linear MR analyses of genetically predicted signature lipidomic biomarkers and the risk of DSCs 30

Table S11. Age- and sex-specific subgroup analyses for phenotypic associations between HDL-C concentration and the risk of DSCs 31

Table S12. Age- and sex-specific subgroup analyses for phenotypic associations between LDL-C concentration and the risk of DSCs 32

Table S13. Age- and sex-specific subgroup analyses for phenotypic associations between TG concentration and the risk of DSCs 33

Table S14. Age- and sex-specific linear MR analyses 34

Table S15. Age- and sex-specific stratified MR analyses for the associations between three categories of HDL-C concentration and the risk of DSCs 35

Table S16. Age- and sex-specific stratified MR analyses for the associations between three categories of LDL-C concentration and the risk of DSCs 36

Table S17. Age- and sex-specific stratified MR analyses for the associations between three categories of TG concentration and the risk of DSCs 37

Supplementary Figures 38

Figure S1. Three key assumptions of MR analyses... 38

Figure S2. Sensitivity analyses for phenotypic association patterns between signature lipidomic biomarkers (HDL-C (A), LDL-C (B), and TG (C)) and the risk of DSC after excluding DSC participants diagnosed in the first two-year follow-up time. 39

Figure S3. Sensitivity analyses for phenotypic association patterns between signature lipidomic biomarkers (HDL-C (A), LDL-C (B), and TG (C)) and the risk of DSC additionally adjusting for additional medication use.. 41

Figure S4. Sensitivity analyses for phenotypic association patterns between signature lipidomic biomarkers (HDL-C (A), LDL-C (B), and TG (C)) and the risk of DSC additionally adjusting for fasting time.. 43

Figure S5. Sensitivity analyses for phenotypic association patterns between signature lipidomic biomarkers (HDL-C (A), LDL-C (B), and TG (C)) and the risk of DSC additionally adjusting for signature lipidomic biomarkers.. 45

Figure S6. Causal association patterns between signature lipidomic biomarkers and the risk of DSCs with adjustment of potential confounders... 47

Figure S7. Causal association patterns between signature lipidomic biomarkers and the risk of DSCs in participants aged less than 60 years.. 48

Figure S8. Causal association patterns between signature lipidomic biomarkers and the risk of DSCs in participants aged 60 years or older. 49

Figure S9. Causal association patterns between signature lipidomic biomarkers and the risk of DSCs in female participants.. 50

Figure S10. Causal association patterns between signature lipidomic biomarkers and the risk of DSCs in male participants... 51

Supplementary Methods:

Assessment of phenotypic data

Measurements of HDL-C (Field ID: 30760), LDL-C (Field ID: 30780), and TG (Field ID: 30870) concentrations were respectively analyzed with enzyme immunoinhibition, enzymatic selective protection, and GPO-POD methods in AU5800 supplied from Beckman Coulter. The blood of participants mixed with the anti-coagulant/preservative were collected with clot activator serum separation tube (SST), and then to be clotted for 25–30 min at room temperature before centrifugation at 2500g for 10 min at 4 °C; the time of centrifugation is recorded in the assessment centre IT system. At the central laboratory, all samples were predominantly processed using custom-designed industrial-scale automation systems to generate about 25,000 sample aliquots per day (resulting in 15 million 1.4 ml aliquots for the full cohort). A small proportion of samples were manually aliquoted. The extensive use of automation ensured that all samples were processed quickly, with an average time of 24 ± 2.5 hours between venepuncture and sample storage. This was achieved by ensuring that the samples were processed at the central facility in the same chronological order in which they were collected (https://biobank.ndph.ox.ac.uk/showcase/refer.cgi?id=5636). Moreover, all blood biochemistry assays were conducted in compliance with an internationally recognised standard for testing and calibration laboratories-ISO 17025. A number of quality performance tests were performed to verify that the instrument was suited for routine use during the installation of each analyser, where the following parameters were assessed: (1) within-run and within-laboratory (total) precision; (2) accuracy (or recovery) and bias; (3) linearity and reportable range including the limit of quantification; (4) no carryover from high concentration to low concentration samples; (5) multi-instrument comparison for the sample. Assay-specific Westgard internal quality control multi-rules for sample batch acceptance during analysis were generated from the biological TEA, precision, and bias results (https://biobank.ndph.ox.ac.uk/showcase/refer.cgi?id=1227) .

The diagnoses of six digestive system cancers, including oesophagus cancer (ICD10: C15), stomach cancer (ICD10: C16), colorectal cancer (ICD10: C18-C20), liver cancer (ICD10: C22), gallbladder cancer (ICD10: C23-C24) and pancreas cancer (ICD10: C25), were all coded using International Classification of Diseases, Tenth Revision (ICD-10) codes based on interviews from participants or their proxies through checking hospital inpatient records about the first occurrence of a set of diagnostic codes for those DC outcomes (Field ID: 41270) updated until October 2022.

Baseline characteristics including age (Field ID: 21022), sex (Field ID: 31) and Townsend deprivation index (TDI) (Field ID: 22189) were known before arrival at the Assessment Centre. In general, “age” refers to the age of the participant on the day they attended an Initial Assessment Centre, truncated to whole year. “sex” means the sex of participant. TDI is a census-based index of material deprivation calculated by the combination of four census variables. Positive values of the index indicate areas with high material deprivation, and those with negative values indicate relative affluence. A score of 0 represents an area with overall mean values. Additionally, Body mass index (BMI, Kg/m^2) (Field ID: 21001) here was constructed from height and weight measured during the initial Assessment Centre visit (Weight (Kg)/(Standing height $(m)^2$)). Systolic blood pressure (SBP, mmHg) (Field ID: 4080) and diastolic blood pressure (DBP, mmHg) (Field ID: 4079) were measured in the seated resting position with a minimum of 1 minute between two blood pressure readings. Mean SBP and DBP using both blood pressure measurements were performed using an appropriately sized cuff and an Omron blood pressure monitor. Fasting time (Field ID: 74) was the interval (hours) between consumption of food or drink and blood sample(s) being taken.

Education qualification (Field ID: 6138) and current employment status (Field ID: 6142) were both inquired with touchscreen questionnaires. ACE touchscreen question would be asked "Which of the following qualifications do you have? (You can select more than one)" for education qualification and "Which of the following

describes your current situation? (You can select more than one answer)" for employment status. In this study, for education qualification, participants with "none of educational qualifications" were encoded with "0". Participants with "A/AS levels or equivalent, A/GCSEs levels or equivalent, CSEs levels, and NVQ or HND or HNC levels or equivalent" were encoded with "1". Participants with "College or university degree, and professional qualifications" were encoded with "2". Meanwhile, for employment status, participants answering "Retired, looking after home and/or family, unable to work because of sickness or disability, unemployed, doing unpaid or voluntary work, and full or part-time student" were defined as "None employed"(labelled as "0"). Participants answering "In paid employment or self-employed" were defined as "Current employed"(labelled as "1"). Participants preferring not to answer above two questions were excluded.

Measurements of alanine aminotransferase (ALT, U/L) (Field ID: 30620) and aspartate aminotransferase (AST, U/L) (Field ID: 30650) concentrations were analyzed with an enzymatic rate method in AU5800 supplied from Beckman Coulter, and measurements of glycated haemoglobin (HbA1c, mmol/mol) (Field ID: 30750) were analyzed with a high-performance liquid chromatography (HPLC) method in Bio-Rad Variant II Turbo supplied from Bio-Rad Laboratories.

Smoking status (Field ID: 20116) summarized the participants' current or past smoking status: "Never smoking" was coded as "0", "Previous smoking" is coded as "1", and "Current smoking" was coded as "2". Participants preferring not to answer were excluded in this study. Alcohol drinking status (Field ID: 20117) summarized the participants' current or past alcohol drinking status: "Never drinking" was coded as "0", "Previous drinking" was coded as "1", and "Current drinking" was coded as "2". Participants preferring not to answer were excluded in this study. For physical activity level (Field ID: 6164), ACE touchscreen question "In the last 4 weeks did you spend any time doing the following? (You can select more than one answer)". In this study, participants answering "Walking for pleasure (not as a means of transport)", and "Light DIY (eg: pruning, watering the lawn)" were defined as "doing light/moderate

activity" (labelled as "1"). Participants answering "Heavy DIY (eg: weeding, lawn mowing, carpentry, digging)", "Strenuous sports", or "Other exercises (eg: swimming, cycling, keep fit, bowling)" were defined as "doing Heavy/Strenuous activity" (labelled as "2"). Participants answering "None of the above" were labelled as "0". Participants preferring not to answer were excluded in this study.

For clinical covariates including health-related outcomes (diabetes, cerebral infarction, ischaemic heart disease, and primary hypertension,), medication use (female: medication for cholesterol, blood pressure, diabetes, or take exogenous hormones; male: medication for cholesterol, blood pressure or diabetes) and family history (family cancer history). Diagnoses of health-related outcomes (Field ID: 41270) were all coded according to the International Classification of Disease version 10 (ICD-10). For diabetes, participants with either of following items "E10 Insulin-dependent diabetes mellitus", "E11 Non-insulin-dependent diabetes mellitus", "E12 Malnutrition-related diabetes mellitus", "E13 Other specified diabetes mellitus" and "E14 Unspecified diabetes mellitus" were included in this study, and other participants without cerebral infarction were labelled as "0". For cerebral infarction, participants with either of following items "I63 Cerebral infarction" and "I64 Stroke, not specified as haemorrhage or infarction" were labelled as "1", and other participants without cerebral infarction were labelled as "0". For ischaemic heart disease, participants with either of following items "I20 Angina pectoris", "I21 Acute myocardial infarction", "I22 Subsequent myocardial infarction", "I23 Certain current complications following acute myocardial infarction", "I24 Other acute ischaemic heart diseases" and "I25 Chronic ischaemic heart disease" were included in this study, and other participants without ischaemic heart disease were labelled as "0". For primary hypertension, participants with "I10 Essential (primary) hypertension" were included in this study, and other participants without primary hypertension were labelled as "0". Medication use and family cancer history were both inquired with touchscreen questionnaire. For medication use (Field ID: 6153 and 6177), ACE touchscreen question "Do you regularly take any of the following medications? (you can select more than one

answer)" for females and males. Female participants regularly taking " medication for cholesterol, blood pressure, diabetes, or take exogenous hormones", and male participants regularly taking "medication for cholesterol, blood pressure or diabetes" were labelled as "1". Participants not regularly taking above medications were labelled as "0". Participants who preferred not to answer or don't know the answers about these questions were excluded in this study. For family cancer history (Field ID: 20110 and 20107), ACE touchscreen questions, both asked "Has/did your mother/father ever suffer from? (You can select more than one answer)" for different sets of illnesses. Mother of participants diagnosed with "Breast cancer", "Bowel cancer" and "Lung cancer", and father of participants with "Prostate cancer", "Bowel cancer" and "Lung cancer" were labelled as "1". Mother/father of participants were diagnosed with other diseases were labelled as "0". Participants "Preferring not to answer" or "Do not know" were excluded from this study.

Quality control for genotype data

The UKB genetic data were assayed with UK Biobank Lung Exome Variant Evaluation (UK BiLEVE) study and Applied Biosystems UK BiLEVE Axiom Array by Affymetrix, both of which share 95% of marker content. SHAPEIT3 and IMPUTE4 was mainly used to perform genotype imputation. More detailed information about genotype imputation has been described in else [1]. In this study, rigorous quality control for genotype data and sample data was conducted to guarantee the high standard for calculation of polygenic risk score (PRS). For UKB participants, genetic variants that did not satisfy filtering criteria (significant deviations from Hardy Weinberg equilibrium (Hardy-Weinberg P <1E-04), minor allele frequency (MAF) < 0.01, SNPs with genotype call rate < 0.95 and individuals call rate < 0.95) [2] were excluded. Moreover, sample quality control was applied to exclude participants with excessive genetic relatedness (ten or more third-degree relatives), deviation of mean heterozygosity, sex mismatch, non-white European population and missing QC metrics.

SNP-based MR analyses

SNP-based MR analyses were conducted with IVW (multiplicative random-effect), weighted median, MR-Egger, and MR-PRESSO. IVW method uses the reciprocal of the variance of each IV as a weight to calculate the causal estimate of a single IV, and the causal estimate corresponding to each IV can be summed into a weighted estimate as a whole [3]. In further sensitivity analysis, weighted median estimate is the median of the distribution of all IVs estimates sorted by weight, and the weight of each IV causal estimate depends on the accuracy of the estimation. In cases where at least half of SNPs are valid IVs, weighted median can provide a consistent estimate of final effect [4]. The MR-Egger method does not force the regression line to pass through the origin, allowing the included IVs to have directional pleiotropy. When the intercept of MR-Egger analysis is significantly different from 0, it indicates that there is directional gene pleiotropy; when the intercept of the regression is 0, or the intercept is not statistically significant ($P > 0.05$), the slope of MR-Egger represents the causal estimate of exposure on the outcome [5]. Therefore, the MR-Egger method can be utilized to examine and correct the horizontal pleiotropy. MR-PRESSO (Pleiotropy RESidual Sum and Outlier) analysis was performed with the global test detecting potential horizontal pleiotropy, and the outlier test [6].

Reference

1. Bycroft C, Freeman C, Petkova D, Band G, Elliott LT, Sharp K, et al. The UK Biobank resource with deep phenotyping and genomic data. Nature. 2018; 562: 203-9.

2. Anderson CA, Pettersson FH, Clarke GM, Cardon LR, Morris AP, Zondervan KT. Data quality control in genetic case-control association studies. Nat Protoc. 2010; 5: 1564-73.

3. Burgess S, Butterworth A, Thompson SG. Mendelian randomization analysis with multiple genetic variants using summarized data. Genet Epidemiol. 2013; 37: 658-65.

4. Bowden J, Davey Smith G, Haycock PC, Burgess S. Consistent Estimation in

Mendelian Randomization with Some Invalid Instruments Using a Weighted Median Estimator. Genet Epidemiol. 2016; 40: 304-14.

5. Bowden J, Davey Smith G, Burgess S. Mendelian randomization with invalid instruments: effect estimation and bias detection through Egger regression. Int J Epidemiol. 2015; 44: 512-25.

6. Verbanck M, Chen C-Y, Neale B, Do R. Detection of widespread horizontal pleiotropy in causal relationships inferred from Mendelian randomization between complex traits and diseases. Nat Genet. 2018; 50: 693-8.

Supplementary Tables

Table S1. Characteristics of European-ancestry cohorts for summary-level GWAS datasets (Excluding UK biobank)*

Cohort	Country of recruitment	Ancestry	N (N_{total}=950,886)	Genotyping Array
WHI-MOPMAP	USA	EUR	90	Affymetrix Gene Titan, Axiom Genome-Wide Human CEU I Array Plate
WHI-GECCO	USA	EUR	119	Cytochip 370K
GRMIC	Greece	EUR	173	Illumina Human Core Exome
CARDIOGENICS-CAD Case	UK, France, Germany	EUR	218	Human660W-Quad_v1_A
THISEAS- CAD Case	Greece	EUR	266	HumanOmniExpress-12v1-Multi_B
ROSMAP2	USA	EUR	304	Affy 6.0 and Illimina Omni Express
ASPS-Fam	Austria	EUR	327	Affymetrix Genome-Wide Human SNP Array 6.0
WHI-HIPFX	USA	EUR	401	Illumina 550K and 610K
EPOZ	Netherlands	EUR	416	GSA
NAFLD	Greece	EUR	443	Illumina Human Core Exome
CTMM	Netherlands	EUR	465	Affymetrix Tx array
CFS	USA	EUR	549	Illumina Omni
BioMe	USA	EUR	566	Illumina HumanOmniExpressExome-8 v1.0
THISEAS- CAD Control	Greece	EUR	575	HumanOmniExpress-12v1-Multi_B
Hoorn	Netherlands	EUR	580	Affymetrix Axiom
LOLIPOP-EWA	UK	EUR	582	Affymetrix 500K
GerMIFS1	Germany	EUR	584	Affymetrix Mapping 500K
LURIC-Controls	Germany	EUR	610	Affymetrix 6.0 + Illumina 200k Metabochip
LOLIPOP-EWP	UK	EUR	644	Perlegen custom
Health2008	Denmark	EUR	740	Illumina HumanOmniExpress-24v1-0_A and HumanOmniExpress-24v1-1_A
Cilento700K	Italy	EUR	745	Illumina OmniExpress
GOLDN	USA	EUR	801	Affymetrix Genome-Wide Human SNP Array 6.0
GerMIFS2	Germany	EUR	820	Affymetrix Genome-Wide Human SNP Array 6.0
SDC	Denmark	EUR	825	Illumina HumanOmniExpress-24v1-0_A and HumanOmniExpress-24v1-1_A

BMES	Australia	EUR	828	Illumina Human Core Exome Array
ASPS	Austria	EUR	829	Illumina Human610-Quad BeadChip
Cilento370K	Italy	EUR	833	Illumina 370K
FINCAVAS	Finland	EUR	844	Illumina HumanCoreExome-12 v1.1
QFS	Canada	EUR	877	Illumina 610-Quad chip
SORBS	Germany	EUR	883	500K Affymetrix GeneChip (250K Sty and 250K Nsp arrays, Affymetrix, Inc) and Affymetrix Genome-Wide Human SNP Array 6.0
Raine Study	Australia	EUR	903	Illumina Human 660W Quad Array
Genie UK-RoI	UK & Republic of Ireland	EUR	904	Infinium CoreExome
POMAK (HELIC-Pomak)	Greece	EUR	926	Illumina OmniExpress & HumanExome
LOLIPOP-EW610	UK	EUR	927	Illumina Human610
POPGEN	Germany	EUR	940	Affymetrix Axiom
MrOS Sweden	Sweden	EUR	943	Illumina HumanOmni1_Quad_v1-0 B array
AUGUR	Germany	EUR	983	Illumina Infinium Global Screening Array 24
SHIPT	Germany	EUR	985	Illumina Infinium® HumanOmni2.5 BeadChip
eMERGE Children	USA	EUR	990	Affymetrix: 6.0, AffyAxiom, Immunina: OmniExpress, Omni1-5
VIS	Croatia	EUR	994	Illumina HumanHap300v1
GRAPHIC	UK	EUR	1,005	Illumina HumanOmniExpress-12v1
AE	Netherlands	EUR	1,015	Affymetrix SNP 5.0; Affymetrix Axiom CEU
STRIP	Finland	EUR	1,043	Illumina InfiniumCoreExome-24 v1.1
HyperGen	USA	EUR	1,058	Affy 5.0
RISC	Europe	EUR	1,064	Affymetrix 6.0
TRAILS-POP	Netherlands	EUR	1,096	Illumina Cyto SNP12 v2
WHI-LLS	USA	EUR	1,096	HumanOmniExpressExome-8v1_B
OGP	Italy	EUR	1,100	Affymetrix 500K Gene Chip
CHIP	USA	EUR	1,112	Illumina Human CoreExome
ROSMAP1	USA	EUR	1,162	Affy 6.0 and Illimina Omni Express
INCHIANTI	Italy	EUR	1,206	Illumina Infinium HumanHap550 genotyping chip
MANOLIS (HELIC-MANOLIS)	Greece	EUR	1,215	Illumina OmniExpress & HumanExome
GENOA	USA	EUR	1,271	Affymetrix 6.0

EPICPotsdam	Germany	EUR	1,306	InfiniumOmniExpressExome-8v1-3
FVG	Italy	EUR	1,341	Illumina OmniExpress
DIACORE	Germany	EUR	1,416	Axiom UK Biobank Array
ORCADES	Scotland	EUR	1,421	Illumina Hap300, Illumina Omni1, Illumina OmniExpress
Health2010	Denmark	EUR	1,439	Illumina HumanOmniExpress-24v1-0_A and HumanOmniExpress-24v1-1_A
JoCoOA	USA	EUR	1,463	Illumina Infinium 1M-Duo
WHI-ONCO	USA	EUR	1,527	Infinium OncoArray-500K BeadChip
GOMAP	Greece	EUR	1,528	Illumina HumanCoreExome
ALSPAC: Mothers	UK	EUR	1,536	Illumina Human660W Quad
BRIGHT	UK	EUR	1,540	Affymetrix GeneChip 500k
CARDIA	USA	EUR	1,540	Affymetrix 6.0
LBR	UK	EUR	1,604	Affymetrix UK Biobank Axiom Array R3
HABC	USA	EUR	1,608	Illumina Human1M-Duov3_B
NhsNhs2HpfsHuco	USA	EUR	1,621	Illumina HumanCoreEx multiple versions
PROCARDIS- Controls	UK, Sweden, Italy, Germany	EUR	1,635	Illumina 600K & Illumina 1M
FTC	Finland	EUR	1,693	Human670-QuadCustom; Human610-Quad; HumanCoreExome-12v1-0; HumanCoreExome-12v1-1; HumanCoreExome-24v1-0
FINRISK3	Finland	EUR	1,766	HumanHap 610K
PARC	USA	EUR	1,798	Illumina 300K and 610K
RS2	Netherlands	EUR	1,934	Illumina 550K
CHOP	USA	EUR	1,949	Illumina 550,610
EUGENDA	Netherlands and Germany	EUR	1,959	Illumina HumanCoreExome with custom content
NhsHpfsPhsOmni	USA	EUR	1,973	Illumina OmniExpress multiple versions
EpiHealth	Sweden	EUR	2,000	Illumina HumanCoreExome
VIKING	Scotland	EUR	2,034	Illumina HumanOmniExpressExome8v1-2_A
NESDA	The Netherlands	EUR	2,039	Affymetrix-Perlegen, Affymetrix 6.0
Vejle- T2D Cases	Denmark	EUR	2,042	Illumina HumanOmniExpress-24v1-0_A and HumanOmniExpress-24v1-1_A
HCS	Australia	EUR	2,112	Affymetrix Kaiser Axiom
FUSION	Finland	EUR	2,188	Illumina Infinium II HumanHap300 BeadChip
Vejle- T2D Controls	Denmark	EUR	2,201	Illumina HumanOmniExpress-24v1-0_A and HumanOmniExpress-24v1-1_A

DGI	Finland, Sweden	EUR	2,246	Affymetrix 500K
LURIC-Cases	Germany	EUR	2,329	Affymetrix 6.0 + Illumina 200k Metabochip
FINRISK4	Finland	EUR	2,415	Illumina Human OmniExpress
YFS	Finland	EUR	2,443	Illumina 670k Custom
NSHD	UK	EUR	2,449	DrugDev consortium array
PMBB	USA	EUR	2,470	Illumina Quad Omni
ASCOT-SC	Sweden, Norway, Denmark	EUR	2,486	Illumina Human Omni Exome Express v8.1
NICOLA	Northern Ireland, UK	EUR	2,488	Infinium CoreExome-24 v1.1
HNR	Germany	EUR	2,556	HumanOmniExpress-12v1_H_fwdfwd; HumanOmniExpress-12v1-1_B; HumanOmni1M-Quadv10-H; HumanOmniExpressExome8v1-2_B
GoDARTS	Scotland	EUR	2644	Affymetrix 6.0/Illumina omni-express
MESA	USA	EUR	2,674	Affymetrix 6.0
KORCULA	Croatia	EUR	2,686	Illumina HumanHap 370CNV DUO/QUAD Phase 1
ERF	Netherlands	EUR	2,762	Illumina 318K, Illumina 370K and Affymetrix 250K
NhsNhs2HpfsPhsIllu	USA	EUR	2,823	Illumina HumanHap multiple versions
KORA S4/F4	Germany	EUR	2,876	Affymetrix Axiom
RS3	Netherlands	EUR	2,963	Illumina 550K
Health2006	Denmark	EUR	2,981	Illumina HumanOmniExpress-24v1-0_A and HumanOmniExpress-24v1-1_A
NhsHpfsAffy	USA	EUR	2,992	Affymetrix 6.0
NhsNhs2HpfsPhsOnco	USA	EUR	3,054	Illumina OncoArray
CHS	USA	EUR	3,158	Illumina 370CNV
AGES	Iceland	EUR	3,214	Illumina Hu370CNV
FINRISK1	Finland	EUR	3,274	Illumina Human CoreExome
RS1	Netherlands	EUR	3,290	Illumina 550K
MHIB	Canada	EUR	3,313	MEGA
DCS	Netherlands	EUR	3,351	Illumina HumanCoreExome
WHII	UK	EUR	3,399	DrugDev consortium array
PREVEND	The Netherlands	EUR	3,428	Illumina Cyto SNP12 v2 array
FINRISK7	Finland	EUR	3,438	Illumina Human CoreExome
FINRISK5	Finland	EUR	3,442	Illumina Human CoreExome

TUK	UK	EUR	3,569	HumanHap300, HumanHap610Q, 1M-Duo and 1.2MDuo 1M
GerMIFS5	Germany	EUR	3,632	Affymetrix Genome-Wide Human SNP Array 6.0
FamHS	USA	EUR	3,777	Illumina 550K, Illumina 610K, Illumina 1mil
ASCOT-UK	UK	EUR	3,804	Illumina HumanCNV 370
KORA S3/F3	Germany	EUR	4,034	Illumina Omni 2.5
SHIP	Germany	EUR	4,035	Affymetrix Genome-Wide SNP Array 6.0
WHI-GARNET	USA	EUR	4,199	Illumina HumanOmni1-Quad v1-0 B
PROCARDIS-Cases	UK, Sweden, Italy, Germany	EUR	4,218	Illumina 600K & Illumina 1M
EURAC (CHRIS)	Italy	EUR	4,347	Illumina HumanOmniExpressExome
LLFS	USA & Denmark	EUR	4,459	Illumina Omni 2.5
GENERISK	Finland	EUR	4,697	Illumina Human CoreExome
NBS	The Netherlands	EUR	4,836	Illumina Omniexpress -12 & -24
LIFE-Adult	Germany	EUR	4,956	Affymetrix Axiom CEU
GCKD	Germany	EUR	4,988	Omni2.5Exome BeadChip array
GLACIERV2	Sweden	EUR	5,090	InfiniumCoreExome-24v1-1
PROSPER	Netherlands, Ireland, Scotland	EUR	5,187	Illumina 660K
MIGEN	US	EUR	5,210	Affymetrix 6.0
NFBC66	Finland	EUR	5,303	Illumina HumanCNV370DUO Analysis BeadChip
CoLaus	Switzerland	EUR	5,434	Affymetrix 500K
FINRISK2	Finland	EUR	5,457	Illumina Human CoreExome
WHI-WHIMS	USA	EUR	5,606	HumanOmniExpressExome-8v1_B
SardiNIA	Italy	EUR	5,651	Illumina Metabochip + Immunochip + OmniExpress + ExomeChip
NEO	Netherlands	EUR	5,688	Illumina HumanCoreExome-24v1_A Beadchip
B58C	UK	EUR	6,007	British 1958 birth cohort
ALSPAC: Children	UK	EUR	6,055	Illumina HumanHap550 Quad
Inter99	Denmark	EUR	6,170	Illumina HumanOmniExpress-24v1-0_A and HumanOmniExpress-24v1-1_A
Danfund	Denmark	EUR	6,799	Illumina HumanOmniExpress-24v1-0_A and HumanOmniExpress-24v1-1_A
OBB	UK	EUR	7169	Affymetrix Axiom UK Biobank array
FINRISK6	Finland	EUR	7,397	Illumina Human CoreExome

EXTEND	UK	EUR	7,627	Not reported
TWINGENE	Sweden	EUR	8,057	lllumina HumanOmniExpress
NTR	The Netherlands	EUR	8,070	Perlegen-Affymetrix, Affymetrix 6.0, Axiom, Illumina 660, 1M, GSA + NTR-GONL
FHS	USA	EUR	8,086	Affymetrix 250K Mapping NspI (AN) and Affymetrix 250K Mapping StyI (AS)
Fenland	UK	EUR	8,933	Affymetrix Axiom UKBiobank
ARIC	USA	EUR	9,123	Affymetrix 6.0
USOC (UKHLS)	UK	EUR	9,807	Illumina HumanCoreExome
METSIM	Finland	EUR	10,037	Illumina OmniExpress
MGI	USA	EUR	10,994	SemiCustom Illumina HumanCoreExome v12.1
HRS	USA	EUR	11,301	Illumina Omni2.5 Beadchip
QIMR	Australia	EUR	13,372	Illumina317,370,610,660,Core+Exome,PsychArray,Omni2.5,OmniExpress
GS20K	UK	EUR	20,395	HumanOmniExpressExome8v1-2_A, HumanOmniExpressExome-8v1_A
DiscovEHR	USA	EUR	21,420	Illumina Human OmniExpress Exome Chip
WGHS	USA and Canada	EUR	23,185	HumanHap300 Duo and iSelect
eMERGE Adults	USA	EUR	25,086	Affymetrix: 6.0, AffyAxiom, Immunina: OmniExpress, Omni1-5
HUNT	Norway	EUR	69,345	Illumina HumanCoreExome
DECODE	Iceland	EUR	137,772	Illumina HumanHap and Omni
MVP	USA	EUR	225,770	MVP Genotyping Array v1.0

*Graham SE, Clarke SL, Wu K-HH, Kanoni S, Zajac GJM, Ramdas S, et al. The power of genetic diversity in genome-wide association studies of lipids. Nature. 2021; 600: 675

Table S2. Statistical power of signature lipidomic biomarkers in relation to different DSC outcomes in Mendelian randomization analyses (*N* = 319,568).

Outcomes	No./Cases	Power (%)		
		HDL-C	LDL-C	TG
**Oesophagus (C15)**	319,568/901	9.7	4.9	6.1
**Stomach (C16)**	319,568/669	26.4	86.3	4.4
**Colorectal (C18-C20)**	319,568/4410	27.3	28.0	62.7
**Liver (C22)**	319,568/336	46.0	22.7	51.0
**Gallbladder (C23-C24)**	319,568/228	67.5	27.4	87.2
**Pancreas (C25)**	319,568/893	18.6	13.8	37.9

Statistical power was calculated with the online tool (https://sb452.shinyapps.io/power/)

Table S3. SNPs used for calculating HDL-C-PRS

Exposure	SNP	Chr.	Position_hg19	Beta	SE	EAF	Effect allele	Other allele	*P* value
HDL-C	rs1007766	7	1034903	0.010	0.002	0.490	G	A	1.34E-09
HDL-C	rs1037117	15	102068658	0.011	0.002	0.252	A	G	1.41E-09
HDL-C	rs11038700	11	45899791	0.012	0.002	0.277	C	A	1.31E-11
HDL-C	rs1109560	12	123757905	0.046	0.003	0.094	A	C	1.21E-63
HDL-C	rs11161570	1	85721911	0.009	0.002	0.497	T	C	2.40E-08
HDL-C	rs111731678	7	130418744	-0.013	0.002	0.196	A	T	9.40E-10
HDL-C	rs11226111	11	103878499	-0.014	0.002	0.152	C	T	7.31E-10
HDL-C	rs112792569	16	56658745	0.064	0.006	0.019	C	G	2.27E-27
HDL-C	rs113271699	11	116650571	0.081	0.007	0.014	A	C	6.29E-33
HDL-C	rs1149453	8	64532168	-0.011	0.002	0.187	T	G	2.73E-08
HDL-C	rs11642733	16	30969061	-0.015	0.003	0.107	A	G	4.18E-09
HDL-C	rs11644601	16	15172118	0.018	0.002	0.299	C	T	2.43E-23
HDL-C	rs11651957	17	17497409	-0.024	0.003	0.060	A	G	3.99E-12
HDL-C	rs116862304	16	56388893	0.048	0.006	0.018	T	C	5.33E-14
HDL-C	rs116998829	8	19520669	0.027	0.004	0.036	G	A	1.95E-09
HDL-C	rs117687565	18	47147524	0.069	0.008	0.012	T	C	5.98E-20
HDL-C	rs118175831	18	47066204	0.046	0.005	0.024	T	C	4.28E-17
HDL-C	rs11870735	17	481604	-0.015	0.002	0.175	T	C	2.61E-12
HDL-C	rs11879735	19	48147444	-0.019	0.003	0.113	T	A	1.35E-14
HDL-C	rs12023488	1	212498198	0.009	0.002	0.374	T	C	1.08E-08
HDL-C	rs12028554	1	154223101	-0.009	0.002	0.437	A	G	4.27E-08
HDL-C	rs12216486	6	160329616	-0.021	0.003	0.091	A	G	1.28E-13
HDL-C	rs12347784	9	107689289	-0.018	0.003	0.098	G	T	7.46E-11
HDL-C	rs12358424	10	33641149	-0.027	0.003	0.089	G	T	3.46E-21
HDL-C	rs12506484	4	38402147	-0.017	0.003	0.092	T	C	3.61E-10
HDL-C	rs12585801	13	81393264	0.014	0.002	0.150	A	G	5.08E-10
HDL-C	rs12721090	11	116700775	0.081	0.004	0.033	T	C	2.62E-72
HDL-C	rs12934512	16	71965230	0.011	0.002	0.268	G	C	1.02E-09
HDL-C	rs13252421	8	19613947	-0.041	0.007	0.013	G	C	9.35E-09
HDL-C	rs13404250	2	188116196	0.010	0.002	0.331	T	C	1.14E-09
HDL-C	rs1421656	5	72928034	-0.014	0.002	0.282	A	G	2.17E-15
HDL-C	rs142638686	8	19892948	0.084	0.007	0.012	T	C	8.14E-30
HDL-C	rs1438588	5	153444950	0.012	0.002	0.366	A	G	1.21E-12
HDL-C	rs144164168	20	44476037	-0.045	0.006	0.020	T	C	1.68E-14
HDL-C	rs147464145	16	56988503	0.160	0.008	0.010	T	C	7.54E-92
HDL-C	rs1661052	11	2942593	-0.025	0.003	0.092	G	A	5.17E-18
HDL-C	rs1664781	5	53276301	0.016	0.002	0.314	G	A	8.02E-20
HDL-C	rs1689796	1	182151236	-0.027	0.002	0.325	G	T	1.01E-57
HDL-C	rs16940712	15	59027139	-0.037	0.005	0.024	G	A	5.17E-12
HDL-C	rs16942887	16	67928042	0.073	0.002	0.124	A	G	1.00E-200
HDL-C	rs1714008	4	57945621	-0.009	0.002	0.414	G	A	3.81E-08
HDL-C	rs17166	8	19969011	0.031	0.005	0.025	T	C	4.11E-09
HDL-C	rs17432483	2	63023836	-0.021	0.003	0.061	C	T	1.61E-09
HDL-C	rs1767141	1	23734350	-0.024	0.003	0.094	A	C	2.20E-18
HDL-C	rs17710668	17	3880546	-0.012	0.002	0.192	G	C	4.59E-09
HDL-C	rs17849502	1	183532580	-0.021	0.004	0.046	T	G	3.51E-08

HDL-C	rs17875416	10	115796337	0.030	0.002	0.115	G	A	2.52E-34
HDL-C	rs187544997	8	19890641	-0.073	0.008	0.013	G	C	3.63E-21
HDL-C	rs192154135	1	151279323	0.047	0.007	0.013	T	C	1.47E-10
HDL-C	rs1949102	8	60654261	0.011	0.002	0.262	G	A	4.87E-09
HDL-C	rs2090032	2	20372547	-0.012	0.002	0.491	C	G	2.70E-14
HDL-C	rs2172071	10	122968030	-0.012	0.002	0.316	C	T	7.89E-12
HDL-C	rs2185454	14	65870594	-0.012	0.002	0.339	G	T	1.50E-12
HDL-C	rs2229741	21	16340289	-0.009	0.002	0.422	T	C	2.11E-08
HDL-C	rs2254038	21	30745722	-0.011	0.002	0.411	T	C	6.60E-11
HDL-C	rs2294927	22	44382684	-0.011	0.002	0.392	C	T	9.21E-11
HDL-C	rs2325976	20	2837772	0.010	0.002	0.364	T	C	1.02E-09
HDL-C	rs235374	21	46294986	-0.019	0.002	0.431	C	G	4.75E-31
HDL-C	rs2364482	12	6502131	0.012	0.002	0.187	G	T	4.62E-09
HDL-C	rs2376180	4	37154148	-0.009	0.002	0.489	T	A	3.74E-08
HDL-C	rs257377	7	106801088	0.013	0.002	0.198	T	G	4.27E-11
HDL-C	rs2811466	3	129299265	0.019	0.003	0.104	A	G	7.13E-13
HDL-C	rs34409228	7	1102674	0.029	0.002	0.126	T	C	2.01E-33
HDL-C	rs35137994	19	8429066	0.039	0.003	0.059	T	C	6.23E-30
HDL-C	rs35511894	15	58723709	0.131	0.009	0.009	T	C	4.15E-50
HDL-C	rs3767298	1	205037354	-0.014	0.002	0.179	A	G	5.67E-11
HDL-C	rs3775217	4	88019467	0.015	0.002	0.378	G	A	4.59E-20
HDL-C	rs3803357	15	40751555	-0.011	0.002	0.499	C	A	9.56E-12
HDL-C	rs3806415	1	156698265	0.014	0.002	0.323	T	C	2.90E-17
HDL-C	rs3824477	9	107588328	0.105	0.005	0.033	A	G	1.08E-117
HDL-C	rs3859027	16	88551465	-0.010	0.002	0.392	C	T	1.12E-08
HDL-C	rs39324	7	116969876	-0.013	0.002	0.367	G	A	3.78E-15
HDL-C	rs4077194	1	178533832	0.019	0.002	0.487	G	T	7.09E-32
HDL-C	rs4375701	17	45712218	0.017	0.002	0.488	T	C	8.05E-25
HDL-C	rs4675812	2	242395674	-0.014	0.002	0.409	G	A	4.04E-17
HDL-C	rs4904550	14	89804856	-0.015	0.002	0.197	G	A	2.93E-13
HDL-C	rs4969182	17	76393030	-0.031	0.002	0.492	T	C	6.17E-84
HDL-C	rs4984511	15	96704443	0.012	0.002	0.269	T	C	9.22E-11
HDL-C	rs549058	13	51201045	0.020	0.002	0.126	T	G	3.65E-16
HDL-C	rs55888929	6	139301177	0.016	0.002	0.153	T	C	7.02E-13
HDL-C	rs56925758	8	144299352	0.024	0.002	0.197	A	G	3.78E-33
HDL-C	rs5745110	5	172572954	0.015	0.002	0.125	T	C	1.66E-10
HDL-C	rs59312889	19	5101405	-0.018	0.003	0.064	G	C	2.21E-08
HDL-C	rs61731455	5	665295	0.013	0.002	0.185	G	A	5.40E-10
HDL-C	rs62568179	9	107643786	-0.075	0.002	0.122	A	G	1.00E-200
HDL-C	rs6460894	7	12247330	-0.011	0.002	0.324	C	T	7.43E-11
HDL-C	rs647016	1	42659669	-0.012	0.002	0.145	T	A	3.28E-08
HDL-C	rs664732	5	131281458	-0.023	0.004	0.043	C	G	3.11E-09
HDL-C	rs6663226	1	66164748	0.010	0.002	0.365	T	A	2.28E-09
HDL-C	rs6710091	2	239597	0.016	0.002	0.351	G	C	4.76E-22
HDL-C	rs686030	9	15304782	-0.048	0.002	0.142	C	A	1.55E-99
HDL-C	rs6968554	7	17287106	0.013	0.002	0.365	A	G	3.15E-15
HDL-C	rs6992709	8	82414063	0.013	0.002	0.148	A	G	7.20E-09
HDL-C	rs7134375	12	20473758	0.018	0.002	0.433	A	C	2.47E-30
HDL-C	rs7200805	16	56989015	-0.121	0.005	0.027	T	C	3.46E-125

HDL-C	rs72629496	1	2953143	-0.013	0.002	0.166	A	G	1.11E-09
HDL-C	rs7497973	15	23948571	-0.009	0.002	0.487	G	A	5.56E-09
HDL-C	rs75130471	13	94085242	-0.011	0.002	0.246	A	G	2.09E-08
HDL-C	rs75246752	1	145630111	0.069	0.010	0.008	C	G	4.69E-12
HDL-C	rs7541031	1	28374815	-0.012	0.002	0.274	T	C	8.44E-12
HDL-C	rs76166162	1	172380926	-0.020	0.003	0.061	A	G	9.27E-10
HDL-C	rs76533352	16	57150943	0.024	0.003	0.070	G	T	3.45E-14
HDL-C	rs76868109	17	41776943	-0.157	0.005	0.031	C	A	1.00E-200
HDL-C	rs7686914	4	69537915	0.012	0.002	0.471	C	T	2.79E-10
HDL-C	rs77050717	16	56767381	-0.207	0.007	0.015	A	G	1.00E-200
HDL-C	rs77617917	20	44563217	-0.054	0.003	0.076	A	G	1.91E-73
HDL-C	rs78424108	13	41673457	-0.024	0.003	0.069	G	C	6.82E-14
HDL-C	rs7848507	9	128593132	0.011	0.002	0.365	G	T	5.67E-10
HDL-C	rs79612456	15	58766286	0.035	0.005	0.035	G	T	4.42E-14
HDL-C	rs79634051	11	14561945	0.047	0.006	0.024	C	G	7.48E-17
HDL-C	rs79949326	7	6461310	0.030	0.002	0.249	T	C	1.73E-56
HDL-C	rs79968526	16	56911069	-0.065	0.006	0.023	C	T	5.45E-31
HDL-C	rs80204526	18	47066144	0.277	0.008	0.010	A	C	1.00E-200
HDL-C	rs805317	2	54135004	0.012	0.002	0.453	A	T	9.82E-13
HDL-C	rs8130925	21	43725765	0.009	0.002	0.496	T	G	6.01E-09
HDL-C	rs854562	7	94947969	-0.011	0.002	0.313	T	C	9.45E-10
HDL-C	rs876039	7	50308811	0.015	0.002	0.312	C	G	8.41E-19
HDL-C	rs9367175	6	43787755	0.013	0.002	0.306	A	G	1.04E-14
HDL-C	rs9447497	6	76219717	0.009	0.002	0.364	C	T	2.71E-08
HDL-C	rs9608668	22	28545329	-0.010	0.002	0.260	C	T	4.91E-08
HDL-C	rs9608955	22	30900829	-0.017	0.002	0.226	T	C	2.26E-19
HDL-C	rs9976784	21	46907479	-0.018	0.002	0.202	A	G	3.77E-20

Table S4. SNPs used for calculating LDL-C-PRS

Exposure	SNP	Chr.	Position_hg19	Beta	SE	EAF	Effect allele	Other allele	*P* value
LDL-C	rs10184673	2	169827796	0.020	0.002	0.409	G	A	4.55E-31
LDL-C	rs10260606	7	44584551	0.043	0.002	0.201	C	G	1.50E-91
LDL-C	rs10263252	7	1049949	-0.021	0.002	0.208	A	G	4.98E-26
LDL-C	rs1031101	10	54533360	0.013	0.002	0.142	G	A	2.16E-08
LDL-C	rs10814052	9	33853571	0.012	0.002	0.162	A	C	4.93E-08
LDL-C	rs11075253	16	15148646	0.011	0.002	0.302	A	C	6.27E-09
LDL-C	rs11105294	12	89858839	0.012	0.002	0.339	A	G	2.33E-12
LDL-C	rs112575086	4	54429890	-0.015	0.003	0.121	T	C	6.91E-10
LDL-C	rs113548930	7	44847350	0.027	0.005	0.037	A	T	3.05E-09
LDL-C	rs113776580	5	74950420	0.046	0.007	0.014	A	G	2.14E-11
LDL-C	rs115458560	1	110061974	-0.047	0.007	0.014	C	T	3.49E-10
LDL-C	rs11620731	14	70817141	-0.025	0.002	0.147	T	C	1.07E-26
LDL-C	rs116276872	1	110068291	-0.055	0.006	0.019	T	C	2.26E-17
LDL-C	rs11650379	17	29460252	-0.047	0.008	0.013	G	A	6.70E-10
LDL-C	rs117264457	19	45404432	-0.049	0.006	0.020	A	G	7.70E-15
LDL-C	rs117590445	20	61321110	-0.044	0.008	0.014	T	C	8.48E-09
LDL-C	rs11846741	14	64238580	0.017	0.003	0.074	G	A	4.83E-08
LDL-C	rs11870935	17	45732605	0.025	0.002	0.491	G	A	1.38E-51
LDL-C	rs12086676	1	55738663	-0.027	0.002	0.174	T	C	3.29E-34
LDL-C	rs12208357	6	160543148	0.059	0.003	0.069	T	C	2.62E-78
LDL-C	rs12271225	11	5690544	0.016	0.002	0.133	T	A	2.76E-11
LDL-C	rs12320328	12	25408464	-0.026	0.003	0.082	G	A	7.23E-18
LDL-C	rs12451056	17	76396188	-0.020	0.002	0.143	T	C	1.38E-18
LDL-C	rs12533280	7	36171953	0.016	0.002	0.200	T	C	8.96E-15
LDL-C	rs12551960	9	19267440	0.033	0.003	0.079	T	C	3.04E-26
LDL-C	rs12657266	5	156396003	-0.033	0.002	0.365	C	T	3.05E-82
LDL-C	rs12732125	1	55470153	-0.206	0.006	0.018	T	C	1.00E-200
LDL-C	rs13057311	22	50878196	-0.012	0.002	0.267	A	G	2.22E-10
LDL-C	rs13161656	5	141916867	0.022	0.003	0.108	C	T	2.71E-17
LDL-C	rs13403394	2	21942492	-0.028	0.005	0.032	T	C	1.72E-09
LDL-C	rs138204164	4	120123417	-0.014	0.002	0.135	G	C	1.20E-08
LDL-C	rs138483078	1	55489240	0.033	0.002	0.142	A	G	1.70E-44
LDL-C	rs147654565	12	21029965	-0.028	0.005	0.032	A	G	1.35E-09
LDL-C	rs148601586	19	45346666	0.138	0.008	0.011	G	C	1.75E-63
LDL-C	rs150688657	17	7505801	0.021	0.003	0.103	A	G	1.14E-14
LDL-C	rs1584688	3	160267858	0.013	0.002	0.449	C	T	2.98E-14
LDL-C	rs16861497	3	186731327	-0.010	0.002	0.395	T	G	4.37E-09
LDL-C	rs1689801	1	182165484	0.015	0.002	0.322	A	G	2.62E-17
LDL-C	rs16988435	21	33058571	0.032	0.004	0.051	T	C	2.53E-17
LDL-C	rs17036094	1	109834940	-0.121	0.008	0.011	C	A	6.83E-57
LDL-C	rs17532301	13	41609047	-0.018	0.003	0.068	A	G	2.83E-08
LDL-C	rs17580	14	94847262	0.054	0.005	0.034	A	T	8.39E-33
LDL-C	rs1801689	17	64210580	0.095	0.005	0.024	C	A	1.06E-74
LDL-C	rs204473	19	45487519	-0.055	0.006	0.022	A	G	4.51E-22
LDL-C	rs207637	13	33085469	0.011	0.002	0.385	G	T	3.08E-10
LDL-C	rs2302434	7	75630183	0.012	0.002	0.177	T	C	1.99E-08

LDL-C	rs235343	21	46249752	-0.009	0.002	0.436	A	C	3.19E-08
LDL-C	rs2618566	20	17844684	0.040	0.002	0.339	G	T	8.46E-111
LDL-C	rs267733	1	150958836	-0.019	0.002	0.153	G	A	1.07E-17
LDL-C	rs28497720	4	100487370	-0.018	0.002	0.250	T	C	6.75E-22
LDL-C	rs28677840	19	44200667	-0.022	0.003	0.069	A	C	3.08E-12
LDL-C	rs28811342	17	18125845	0.013	0.002	0.200	C	T	8.32E-10
LDL-C	rs3010277	5	72014400	-0.017	0.002	0.210	G	A	6.88E-18
LDL-C	rs34503352	19	58651296	-0.029	0.002	0.161	A	G	4.27E-37
LDL-C	rs35313547	19	58352806	-0.015	0.002	0.158	C	T	5.83E-11
LDL-C	rs3738622	1	235110859	-0.016	0.002	0.203	T	G	3.81E-15
LDL-C	rs3780181	9	2640759	-0.035	0.003	0.071	G	A	8.09E-27
LDL-C	rs414850	21	40543337	-0.012	0.002	0.411	C	A	7.89E-12
LDL-C	rs4234798	4	7219933	-0.010	0.002	0.393	T	G	2.72E-09
LDL-C	rs438568	20	12958687	-0.013	0.002	0.392	A	G	2.84E-15
LDL-C	rs4465	22	35708790	0.014	0.002	0.358	C	T	6.62E-17
LDL-C	rs4751995	10	118397884	-0.016	0.002	0.470	A	G	2.20E-22
LDL-C	rs4804815	19	7842669	-0.012	0.002	0.264	G	C	1.05E-09
LDL-C	rs553427	1	234852760	-0.038	0.002	0.469	C	T	1.83E-116
LDL-C	rs55696093	7	21605973	0.038	0.002	0.206	G	A	4.70E-80
LDL-C	rs55714927	17	7080316	-0.035	0.002	0.187	T	C	6.56E-58
LDL-C	rs55726838	2	44071788	0.070	0.009	0.009	A	G	3.19E-14
LDL-C	rs56223611	7	87083157	-0.014	0.002	0.142	A	C	7.07E-10
LDL-C	rs6093446	20	39780932	0.024	0.002	0.269	A	G	2.78E-38
LDL-C	rs61882680	11	46370636	-0.029	0.005	0.030	T	C	2.51E-09
LDL-C	rs61886346	10	96101364	-0.022	0.003	0.059	T	C	2.96E-10
LDL-C	rs62075819	17	46982544	0.010	0.002	0.358	T	C	8.50E-09
LDL-C	rs62119267	19	45134682	-0.233	0.006	0.020	C	A	1.00E-200
LDL-C	rs6458949	6	53508265	0.013	0.002	0.259	T	G	1.30E-12
LDL-C	rs6967728	7	97915637	-0.016	0.002	0.182	A	G	2.97E-13
LDL-C	rs704	17	26694861	0.020	0.002	0.478	A	G	2.10E-35
LDL-C	rs7046887	9	78736048	0.013	0.002	0.464	T	C	1.14E-15
LDL-C	rs71311871	3	58420613	-0.036	0.003	0.081	G	A	2.57E-34
LDL-C	rs7250652	19	11302606	0.025	0.002	0.439	G	A	1.65E-50
LDL-C	rs72647039	4	74321365	0.037	0.006	0.020	T	C	1.40E-09
LDL-C	rs72729610	4	154190965	-0.013	0.002	0.167	G	A	5.99E-09
LDL-C	rs72768400	5	75046359	-0.034	0.005	0.037	T	G	1.51E-12
LDL-C	rs7300593	12	53790450	0.013	0.002	0.172	C	T	1.34E-09
LDL-C	rs7327867	13	32968591	0.019	0.002	0.474	G	A	2.01E-31
LDL-C	rs76738473	19	33870906	-0.027	0.005	0.028	C	T	3.98E-08
LDL-C	rs76970536	11	126250680	0.059	0.003	0.071	A	G	1.42E-78
LDL-C	rs77502095	12	133119022	0.016	0.002	0.141	A	G	1.08E-10
LDL-C	rs7758845	6	135428537	-0.025	0.002	0.279	C	A	1.06E-41
LDL-C	rs77704739	5	52080909	-0.049	0.004	0.036	C	T	1.27E-28
LDL-C	rs77960347	18	47109955	0.064	0.007	0.012	G	A	5.71E-18
LDL-C	rs78531123	10	104082953	0.020	0.004	0.058	A	G	2.52E-08
LDL-C	rs7864568	9	78212428	-0.016	0.002	0.321	A	G	7.53E-17
LDL-C	rs78946096	3	132188163	-0.041	0.004	0.047	G	A	1.65E-26
LDL-C	rs79391862	15	53739426	-0.065	0.006	0.023	C	A	1.00E-26
LDL-C	rs79429216	19	45445517	0.087	0.009	0.009	A	G	2.02E-21

LDL-C	rs80098465	1	56323794	-0.043	0.005	0.033	A	G	2.93E-20
LDL-C	rs8681	1	45468606	-0.013	0.002	0.255	A	G	2.09E-11
LDL-C	rs880973	2	21078879	-0.012	0.002	0.223	G	A	2.22E-09
LDL-C	rs887829	2	234668570	-0.016	0.002	0.328	T	C	1.09E-19
LDL-C	rs896311	7	25934357	-0.018	0.002	0.303	G	A	5.37E-24
LDL-C	rs9297994	8	59392324	0.032	0.002	0.339	G	A	9.24E-75
LDL-C	rs9410207	9	91404799	-0.021	0.003	0.066	C	T	3.25E-10
LDL-C	rs9423289	10	124704695	-0.022	0.002	0.423	C	T	4.71E-40
LDL-C	rs9653945	3	142660706	-0.011	0.002	0.344	A	G	3.61E-11
LDL-C	rs9824581	3	125066925	-0.011	0.002	0.267	G	A	2.58E-09
LDL-C	rs9909417	17	18679442	0.010	0.002	0.288	A	G	2.01E-08

Table S5. SNPs used for calculating TG-PRS

Exposure	SNP	Chr.	Position_hg19	Beta	SE	EAF	Effect allele	Other allele	*P* value
TG	rs10118003	9	96918293	-0.010	0.002	0.443	C	T	1.94E-09
TG	rs10215153	7	116399131	0.012	0.002	0.308	A	G	7.92E-12
TG	rs10495702	2	20350751	0.012	0.002	0.443	G	A	2.69E-13
TG	rs1056610	22	39080078	0.016	0.002	0.315	C	T	1.14E-20
TG	rs1061599	18	212756	-0.011	0.002	0.272	C	A	6.94E-10
TG	rs10784771	12	69621264	-0.010	0.002	0.447	G	A	3.00E-09
TG	rs10791660	11	103871039	0.012	0.002	0.186	A	C	2.48E-08
TG	rs10818471	9	123394045	-0.012	0.002	0.298	A	G	2.69E-11
TG	rs10950655	7	17308367	-0.011	0.002	0.486	T	C	1.27E-11
TG	rs11113118	12	107199142	0.017	0.002	0.228	A	G	1.08E-18
TG	rs111936426	9	107724707	0.020	0.003	0.085	A	G	5.91E-12
TG	rs11242227	5	134135076	0.018	0.003	0.112	G	A	7.79E-12
TG	rs1126673	4	100045616	-0.014	0.002	0.308	C	T	4.35E-16
TG	rs1133400	10	134459388	0.014	0.002	0.209	G	A	5.36E-13
TG	rs114745683	1	226508335	0.026	0.004	0.042	A	G	1.10E-10
TG	rs117280021	17	64294581	-0.072	0.010	0.008	C	T	6.90E-14
TG	rs117315666	11	116937074	-0.032	0.004	0.044	T	G	1.50E-16
TG	rs117788606	7	72921771	-0.119	0.009	0.008	C	T	2.75E-39
TG	rs118010373	11	14757444	-0.032	0.005	0.030	C	A	7.92E-10
TG	rs12154436	7	150343784	0.014	0.002	0.194	A	G	2.15E-11
TG	rs12654983	5	131098220	0.027	0.004	0.060	A	G	1.99E-14
TG	rs12750321	1	154251626	-0.013	0.002	0.289	G	A	4.49E-13
TG	rs12878001	14	64239629	0.021	0.002	0.162	G	T	9.28E-21
TG	rs12891399	14	104293533	0.012	0.002	0.339	C	T	3.44E-12
TG	rs1292042	17	57934995	0.018	0.002	0.194	G	A	8.91E-19
TG	rs12928099	16	15150505	-0.026	0.002	0.297	A	C	4.59E-48
TG	rs13054650	22	50625079	-0.010	0.002	0.368	C	G	1.38E-09
TG	rs13214458	6	140702211	-0.012	0.002	0.253	T	G	6.19E-10
TG	rs1377587	4	961191	0.011	0.002	0.453	A	G	9.43E-11
TG	rs139260949	17	63978436	-0.022	0.004	0.055	C	T	5.92E-10
TG	rs143076454	16	921179	0.041	0.007	0.016	A	G	1.50E-09
TG	rs144468328	20	57780137	-0.042	0.007	0.014	A	G	4.22E-09
TG	rs148636348	11	396294	0.032	0.006	0.020	A	G	3.43E-08
TG	rs1491508	2	43754992	0.014	0.002	0.195	T	C	1.91E-11
TG	rs1571953	9	35121795	-0.012	0.002	0.219	C	T	1.46E-10
TG	rs165316	1	91533297	-0.015	0.002	0.201	G	A	4.15E-13
TG	rs17036126	3	12287863	0.019	0.002	0.126	T	C	8.59E-15
TG	rs17184382	15	63792486	-0.019	0.002	0.410	C	A	8.90E-31
TG	rs1759415	9	6674253	0.010	0.002	0.411	G	A	2.55E-10
TG	rs17713879	2	254215	-0.012	0.002	0.369	A	G	2.74E-12
TG	rs17779355	10	103928374	-0.027	0.003	0.058	A	G	1.48E-15
TG	rs2159359	17	49239036	-0.011	0.002	0.182	A	C	4.56E-08
TG	rs2237650	7	106694366	-0.009	0.002	0.461	A	C	1.22E-08
TG	rs2302883	11	65824994	0.013	0.002	0.230	C	T	5.40E-11
TG	rs28510484	15	31637569	0.012	0.002	0.164	C	G	2.72E-08
TG	rs3120159	1	26238289	-0.010	0.002	0.406	A	G	1.42E-09

TG	rs34144542	11	116774447	-0.044	0.003	0.069	G	A	1.19E-40
TG	rs3768583	1	183538725	0.009	0.002	0.473	A	T	2.20E-08
TG	rs4253766	22	46623905	0.023	0.003	0.105	T	C	1.41E-18
TG	rs4321388	2	239871065	-0.009	0.002	0.477	A	T	2.63E-08
TG	rs4800116	18	19852666	0.011	0.002	0.470	C	G	3.22E-12
TG	rs4804414	19	7223785	0.022	0.002	0.426	T	C	5.47E-40
TG	rs4822458	22	24265659	0.012	0.002	0.454	C	T	9.41E-13
TG	rs55875049	10	114012078	0.016	0.002	0.185	G	A	1.64E-15
TG	rs56184290	3	129328539	-0.015	0.003	0.100	G	A	1.38E-08
TG	rs5750142	22	36036213	0.009	0.002	0.497	A	G	1.11E-08
TG	rs58301047	8	144296480	-0.014	0.002	0.199	C	G	1.16E-11
TG	rs603104	15	40757035	0.010	0.002	0.448	A	C	2.27E-09
TG	rs6066138	20	45594711	-0.017	0.002	0.269	A	G	1.14E-20
TG	rs6070138	20	56118558	-0.015	0.002	0.409	C	A	6.29E-19
TG	rs6073958	20	44551855	0.045	0.002	0.204	C	T	1.07E-113
TG	rs61900203	11	77759168	0.014	0.002	0.149	G	A	1.23E-08
TG	rs62017272	14	40014604	0.011	0.002	0.199	C	T	4.04E-08
TG	rs62459120	7	44337834	-0.031	0.005	0.024	T	A	8.35E-09
TG	rs625476	9	13705973	0.012	0.002	0.318	C	T	3.19E-12
TG	rs6925800	6	161498311	-0.027	0.004	0.045	T	C	2.88E-11
TG	rs7007256	8	26203081	-0.010	0.002	0.288	G	A	5.79E-09
TG	rs7251733	19	56099951	0.020	0.002	0.156	A	G	6.57E-19
TG	rs727428	17	7537792	0.015	0.002	0.438	T	C	1.99E-18
TG	rs7312441	12	56941146	-0.011	0.002	0.346	A	C	8.95E-10
TG	rs74510325	22	50315382	-0.029	0.004	0.033	G	C	1.53E-10
TG	rs74676173	1	149904474	-0.027	0.003	0.073	C	A	3.70E-18
TG	rs74714416	15	57491156	0.029	0.004	0.040	T	C	2.09E-11
TG	rs7578604	2	121308660	0.016	0.002	0.245	T	G	5.44E-17
TG	rs7608096	2	146373927	0.012	0.002	0.438	T	A	1.27E-12
TG	rs76306670	2	26970769	-0.035	0.005	0.031	T	C	7.20E-14
TG	rs77644716	2	27991368	-0.052	0.003	0.091	C	G	2.16E-73
TG	rs79067298	7	73094267	-0.036	0.004	0.038	A	G	3.12E-17
TG	rs79639690	2	227605895	0.024	0.004	0.046	C	T	5.11E-10
TG	rs79760705	5	53298716	0.026	0.003	0.108	T	G	1.00E-23
TG	rs9912287	17	1630992	0.015	0.002	0.218	A	G	1.97E-13

Table S6. Genetic associations between HDL-C-PRS and the risk of digestive system cancer (*N* = 319,568)

Outcomes[a]	Lowest HDL-C- PRS (<25%, *N*=79,892)	Intermediate HDL-C-PRS (25%~75%, *N*=159,784)	Highest HDL-C- PRS (>75%, *N*=79,892)	P_{trend}[b]
**Oesophagus, n (%)**				0.814
None	79,663 (99.713%)	159,343 (99.724%)	79,661 (99.711%)	
Yes	229 (0.287%)	441 (0.276%)	231 (0.289%)	
**Stomach, n (%)**				0.303
None	79,718 (99.782%)	159,439 (99.784%)	79,742 (99.812%)	
Yes	174 (0.218%)	345 (0.226%)	150 (0.188%)	
**Colorectal, n (%)**				0.117
None	78,769 (98.594%)	157,645 (98.661%)	78,744 (98.563%)	
Yes	1123 (1.406%)	2139 (1.339%)	1148 (1.437%)	
**Liver, n (%)**				0.601
None	79,801 (99.886%)	159,617 (99.895%)	79,814 (99.902%)	
Yes	91 (0.114%)	167 (0.105%)	78 (0.098%)	
**Gallbladder, n (%)**				0.824
None	79,837 (99.931%)	159,672 (99.930%)	79,831 (99.924%)	
Yes	55 (0.069%)	112 (0.070%)	61 (0.076%)	
**Pancreas, n (%)**				0.380
None	79,664 (99.715%)	159,357 (99.733%)	79,654 (99.702%)	
Yes	228 (0.285%)	427 (0.267%)	238 (0.298%)	

[a] Outcomes as the binary variables were displayed as n (%).

[b] Analyses were adjusted with age, sex, assessment centers, genotyping array and the first 10 PCs. P_{trend} value of 0.008 (0.05/6 outcomes, Bonferroni-adjusted *P*) was defined as the threshold for remarkable statistical significance. P_{trend} value between 0.008 and 0.05 was defined as suggestive statistical significance.

HDL-C, high-density lipoprotein cholesterol; HDL-C-PRS, HDL-C polygenic risk score

Table S7. Genetic associations between LDL-C-PRS and the risk of digestive system cancer (*N* = 319,568)

Outcomes[a]	Lowest LDL-C- PRS (<25%, *N*=79,892)	Intermediate LDL-C-PRS (25%~75%, *N*=159,784)	Highest LDL-C- PRS (>75%, *N*=79,892)	P_{trend}[b]
**Oesophagus, n (%)**				0.806
None	79,675 (99.728%)	159,326 (99.713%)	79,666 (99.717%)	
Yes	217 (0.272%)	458 (0.287%)	226 (0.283%)	
**Stomach, n (%)**				**0.014**
None	79,705 (99.766%)	159,438 (99.783%)	79,756 (99.830%)	
Yes	187 (0.234%)	346 (0.217%)	136 (0.170%)	
**Colorectal, n (%)**				0.213
None	78,790 (98.621%)	157,626 (98.649%)	78,742 (98.561%)	
Yes	1102 (1.379%)	2158 (1.351%)	1150 (1.439%)	
**Liver, n (%)**				0.091
None	79,791 (99.874%)	159,624 (99.900%)	79,817 (99.906%)	
Yes	101 (0.126%)	160 (0.100%)	75 (0.094%)	
**Gallbladder, n (%)**				0.332
None	79,842 (99.937%)	159,659 (99.922%)	79,839 (99.934%)	
Yes	50 (0.063%)	125 (0.078%)	53 (0.066%)	
**Pancreas, n (%)**				0.900
None	79,664 (99.715%)	159,344 (99.725%)	79,667 (99.718%)	
Yes	228 (0.285%)	440 (0.275%)	225 (0.282%)	

[a] Outcomes as the binary variables were displayed as n (%).

[b] Analyses were adjusted with age, sex, assessment centers, genotyping array and the first 10 PCs. P_{trend} value of 0.008 (0.05/6 outcomes, Bonferroni-adjusted *P*) was defined as the threshold for remarkable statistical significance. P_{trend} value between 0.008 and 0.05 was defined as suggestive statistical significance.

LDL-C, low-density lipoprotein cholesterol; LDL-C-PRS, LDL-C polygenic risk score

Table S8. Genetic associations between TG-PRS and the risk of digestive system cancer (N = 319,568)

**Outcomes**[a]	**Lowest TG-PRS (<25%, *N*=79,892)**	**Intermediate TG-PRS (25%~75%, *N*=159,784)**	**Highest TG-PRS (>75%, *N*=79,892)**	P_{trend}[b]
**Oesophagus, n (%)**				0.334
None	79648 (99.695%)	159350 (99.728%)	79669 (99.721%)	
Yes	244 (0.305%)	434 (0.272%)	223 (0.279%)	
**Stomach, n (%)**				0.980
None	79724 (99.790%)	159448 (99.790%)	79727 (99.793%)	
Yes	168 (0.210%)	336 (0.210%)	165 (0.207%)	
**Colorectal, n (%)**				0.999
None	78789 (98.619%)	157579 (98.620%)	78790 (98.621%)	
Yes	1103 (1.381%)	2205 (1.380%)	1102 (1.379%)	
**Liver, n (%)**				0.255
None	79817 (99.906%)	159619 (99.897%)	79796 (99.880%)	
Yes	75 (0.094%)	165 (0.103%)	96 (0.120%)	
**Gallbladder, n (%)**				0.344
None	79841 (99.936%)	159673 (99.931%)	79826 (99.917%)	
Yes	51 (0.064%)	111 (0.069%)	66 (0.083%)	
**Pancreas, n (%)**				0.054
None	79696 (99.755%)	159305 (99.700%)	79674 (99.727%)	
Yes	196 (0.245%)	479 (0.300%)	218 (0.273%)	

[a] Outcomes as the binary variables were displayed as n (%).

[b] Analyses were adjusted with age, sex, assessment centers, genotyping array and the first 10 PCs. P_{trend} value 0.008 (0.05/6 outcomes, Bonferroni-adjusted P) was defined as the threshold for remarkable statistical significance. P_{trend} value between 0.008 and 0.05 was defined as suggestive statistical significance.

TG, triglycerides; TG-PRS, TG polygenic risk score

Table S9. Associations between PRSs of signature lipidomic biomarkers and covariates (N = 319,568)

Covariates	Genetic instruments	Beta[a]	SE	*P* value[b]
**HDL-C**	HDL-C-PRS	0.346	0.005	**<2E-16**
	LDL-C-PRS	2.111E-04	0.005	0.966
	TG-PRS	-2.442E-04	0.008	0.976
**LDL-C**	HDL-C-PRS	0.003	0.011	0.785
	LDL-C-PRS	0.664	0.001	**<2E-16**
	TG-PRS	-0.002	0.003	0.505
**TG**	HDL-C-PRS	-0.019	0.014	0.175
	LDL-C-PRS	0.009	0.014	0.520
	TG-PRS	0.962	0.022	**<2E-16**
**BMI**	HDL-C-PRS	-0.020	0.063	0.751
	LDL-C-PRS	-0.016	0.065	0.805
	TG-PRS	0.171	0.106	0.107
**Smoking status**	HDL-C-PRS	0.020	0.026	0.450
	LDL-C-PRS	0.005	0.027	0.846
	TG-PRS	0.030	0.044	0.494
**Alcohol drinking status**	HDL-C-PRS	-0.076	0.054	0.161
	LDL-C-PRS	-0.102	0.057	0.070
	TG-PRS	-0.143	0.092	0.118
**Education qualification**	HDL-C-PRS	-0.010	0.025	0.690
	LDL-C-PRS	0.048	0.026	0.066
	TG-PRS	-0.003	0.016	0.819
**Employment status**	HDL-C-PRS	0.034	0.019	0.074
	LDL-C-PRS	0.053	0.034	0.123
	TG-PRS	0.024	0.056	0.664
**TDI**	HDL-C-PRS	0.025	0.039	0.521
	LDL-C-PRS	-0.050	0.041	0.223
	TG-PRS	0.036	0.066	0.584
**Physical activity level**	HDL-C-PRS	0.052	0.032	0.109
	LDL-C-PRS	0.002	0.034	0.954
	TG-PRS	0.019	0.055	0.728
**Family cancer history**	HDL-C-PRS	0.026	0.029	0.371
	LDL-C-PRS	-0.015	0.030	0.617
	TG-PRS	-0.047	0.049	0.331
**ALT**	HDL-C-PRS	-0.267	0.188	0.156
	LDL-C-PRS	0.290	0.190	0.128
	TG-PRS	0.446	0.308	0.148
**AST**	HDL-C-PRS	-0.195	0.135	0.149
	LDL-C-PRS	0.207	0.139	0.137
	TG-PRS	0.293	0.225	0.193
**SBP**	HDL-C-PRS	-0.564	0.262	**0.031**
	LDL-C-PRS	-0.092	0.256	0.718
	TG-PRS	1.116	0.224	**<0.001**
**DBP**	HDL-C-PRS	0.153	0.142	0.282
	LDL-C-PRS	-0.245	0.147	0.096
	TG-PRS	0.652	0.122	**<0.001**

**Cerebral infarction**	HDL-C-PRS	-0.068	0.089	0.442
	LDL-C-PRS	0.093	0.094	0.323
	TG-PRS	0.013	0.076	0.865
**Ischaemic heart disease**	HDL-C-PRS	-0.226	0.045	**<0.001**
	LDL-C-PRS	0.394	0.049	**<0.001**
	TG-PRS	0.336	0.039	**<0.001**
**Primary hypertension**	HDL-C-PRS	-0.100	0.031	**0.001**
	LDL-C-PRS	0.027	0.032	0.403
	TG-PRS	0.343	0.052	**<0.001**
**HbA1c**	HDL-C-PRS	-0.164	0.084	0.051
	LDL-C-PRS	0.465	0.088	**<0.001**
	TG-PRS	0.914	0.073	**<0.001**
**Diabetes**	HDL-C-PRS	-0.113	0.050	**0.025**
	LDL-C-PRS	0.071	0.053	0.178
	TG-PRS	0.756	0.085	**<0.001**

[a] Estimates of the coefficient (beta) from multiple linear regression analyses of the continuous covariates (BMI and Townsend deprivation index) on the GIVs, from binary logistic regression analysis of binary covariates (employment status, diabetes, cerebral infarction, ischaemic heart disease, primary hypertension, family cancer history and, liver diseases) on the GIVs, and from ordinal logistic regression analysis of the ordered categorical covariates (smoking status, alcohol drinking status, education level, and physical activity level) on the GIVs

[b] *P* value of 2.50E-03 (0.05/20 covariates, Bonferroni-adjusted *P*) was defined as the threshold for remarkable statistical significance. *P* value between 2.50E-03 and 0.05 was defined as suggestive statistical significance.

BMI, body mass index; TDI, Townsend deprivation index; ALT, Alanine aminotransferase; AST, aspartate aminotransferase; SBP, systolic blood pressure; DBP, diastolic blood pressure; HbA1c, glycated haemoglobin; HDL-C, high-density lipoprotein cholesterol; HDL-C-PRS, HDL-C polygenic risk score; LDL-C, low-density lipoprotein cholesterol; LDL-C-PRS, LDL-C polygenic risk score; TG, triglycerides; TG-PRS, TG polygenic risk score; SE, standard error

Table S10. Sensitivity analyses for linear MR analyses of genetically predicted signature lipidomic biomarkers and the risk of DSCs

Outcomes	Methods	HDL-C (mmol/L)		LDL-C (mmol/L)		TG (mmol/L)	
		HR(95% CI)	*P* value[d]	HR(95% CI)	*P* value	HR(95% CI)	*P* value
Oesophagus (C15)	**Sensitivity model 1[a]**	0.975(0.449-2.115)	0.948	1.088(0.504-2.347)	0.830	0.603(0.236-1.540)	0.290
	**Sensitivity model 2[b]**	0.967(0.557-1.676)	0.904	1.087(0.547-2.160)	0.813	0.662(0.272-1.612)	0.363
	**Sensitivity model 3[c]**						
	Weighted median	0.755(0.372-1.532)	0.361	0.525(0.246-1.119)	0.095	1.019(0.320-3.243)	0.975
	MR-Egger	0.821(0.430-1.568)	0.330	0.498(0.239-1.039)	0.066	0.873(0.158-4.814)	0.876
	MR-PRESSO	0.888(0.565-1.396)	0.609	0.943(0.597-1.489)	0.800	1.095(0.507-2.364)	0.819
	MR-Egger intercept	1.003(0.986-1.019)	0.751	1.002(0.983-1.022)	0.830	1.004(0.974-1.035)	0.783
Stomach (C16)	**Sensitivity model 1**	0.682(0.278-1.672)	0.403	**0.343(0.142-0.830)**	**0.018**	0.619(0.209-1.838)	0.388
	**Sensitivity model 2**	0.752(0.398-1.422)	0.381	**0.387(0.176-0.852)**	**0.018**	0.674(0.240-1.893)	0.454
	**Sensitivity model 3**						
	Weighted median	0.775(0.347-1.733)	0.535	0.474(0.213-1.055)	0.067	0.893(0.257-3.100)	0.858
	MR-Egger	0.838(0.400-1.757)	0.640	**0.373(0.162-0.854)**	**0.022**	0.680(0.115-4.039)	0.673
	MR-PRESSO	0.806(0.484-1.343)	0.410	**0.558(0.343-0.910)**	**0.021**	0.903(0.406-2.005)	0.802
	MR-Egger intercept	0.998(0.980-1.017)	0.859	1.014(0.992-1.036)	0.230	1.006(0.975-1.039)	0.694
Colorectal (C18-C20)	**Sensitivity model 1**	1.081(0.761-1.536)	0.663	1.030(0.728-1.457)	0.869	1.069(0.700-1.633)	0.757
	**Sensitivity model 2**	1.042(0.811-1.339)	0.748	1.021(0.747-1.395)	0.896	1.124(0.750-1.685)	0.571
	**Sensitivity model 3**						
	Weighted median	1.134(0.831-1.548)	0.437	1.126(0.816-1.554)	0.470	0.949(0.582-1.549)	0.834
	MR-Egger	1.191(0.887-1.599)	0.469	1.315(0.949-1.824)	0.103	0.807(0.386-1.689)	0.571
	MR-PRESSO	1.132(0.917-1.397)	0.250	1.134(0.928-1.385)	0.221	1.246(0.880-1.763)	0.218
	MR-Egger intercept	0.998(0.991-1.005)	0.604	0.995(0.986-1.004)	0.263	1.009(0.996-1.022)	0.181
Liver (C22)	**Sensitivity model 1**	0.470(0.133-1.662)	0.241	0.298(0.086-1.035)	0.057	3.896(0.841-18.044)	0.082
	**Sensitivity model 2**	0.573(0.234-1.404)	0.224	0.342(0.113-1.037)	0.058	4.025(0.941-17.205)	0.060
	**Sensitivity model 3**						
	Weighted median	0.470(0.187-1.182)	0.108	0.590(0.225-1.544)	0.282	1.384(0.294-6.503)	0.681
	MR-Egger	0.575(0.252-1.310)	0.190	0.442(0.165-1.181)	0.107	0.585(0.071-4.827)	0.620
	MR-PRESSO	0.559(0.273-1.145)	0.112	1.134(0.928-1.385)	0.221	2.440(0.954-6.238)	0.066
	MR-Egger intercept	0.988(0.967-1.008)	0.246	1.014(0.988-1.041)	0.302	1.026(0.988-1.065)	0.180
Gallbladder (C23-C24)	**Sensitivity model 1**	1.437(0.305-6.765)	0.647	0.962(0.209-4.425)	0.961	3.864(0.602-24.823)	0.154
	**Sensitivity model 2**	1.273(0.425-3.814)	0.666	0.967(0.248-3.769)	0.962	4.027(0.692-23.448)	0.121
	**Sensitivity model 3**						
	Weighted median	2.058(0.529-7.999)	0.297	0.565(0.150-2.126)	0.398	1.233(0.146-10.395)	0.847
	MR-Egger	2.404(0.774-7.461)	0.129	1.183(0.306-4.568)	0.808	1.201(0.066-21.880)	0.902
	MR-PRESSO	2.383(0.742-7.655)	0.145	0.584(0.261-1.306)	0.193	1.828(0.504-6.628)	0.361
	MR-Egger intercept	1.005(0.977-1.035)	0.709	0.977(0.942-1.012)	0.198	1.008(0.957-1.061)	0.765
Pancreas (C25)	**Sensitivity model 1**	1.053(0.482-2.297)	0.898	1.075(0.497-2.324)	0.854	1.737(0.678-4.451)	0.250
	**Sensitivity model 2**	1.012(0.582-1.761)	0.966	1.069(0.537-2.129)	0.849	1.911(0.783-4.667)	0.155
	**Sensitivity model 3**						
	Weighted median	0.779(0.373-1.626)	0.505	0.928(0.440-1.961)	0.846	1.023(0.332-3.153)	0.969
	MR-Egger	0.967(0.488-1.915)	0.923	1.184(0.559-2.512)	0.660	1.843(0.367-9.260)	0.460
	MR-PRESSO	1.270(0.780-2.068)	0.338	1.191(0.758-1.871)	0.451	1.317(0.661-2.625)	0.435
	MR-Egger intercept	1.009(0.991-1.026)	0.329	0.999(0.980-1.020)	0.986	0.995(0.967-1.024)	0.744

[a] HR per 1 mmol/L increase of genetically predicted signature lipidomic biomarkers with multiple covariates (reference model: age, sex, assessment centers, genotyping array and the first 10 PCs; sensitivity models: HDL-C: reference model + SBP, ischaemic heart disease, primary hypertension, and diabetes; LDL-C: reference model + ischaemic heart disease and HbA1c; TG: reference model + SBP, DBP, ischaemic heart disease, primary hypertension, HbA1c, and diabetes).

[b] Outcome stages in linear MR analyses of sensitivity model 2 were re-conducted with logistic regression analyses.

[c] SNP-based pleiotropy-robust sensitivity MR analyses with weighted median, MR-Egger, and MR-PRESSO methods. MR-Egger intercept was used to detect the horizontal pleiotropy, and no horizontal pleiotropy was detected ($P_{Egger\_intercept} > 0.05$).

[d] *P* value of 0.008 (0.05/6, Bonferroni-adjusted *P*) was defined as the threshold for remarkable statistical significance. *P* value between 0.008 and 0.05 was defined as suggestive statistical significance.

HDL-C, high-density lipoprotein cholesterol; LDL-C, low-density lipoprotein cholesterol; TG, triglycerides; HR, hazard ratio; IVW, inverse-variance weighted; MR-PRESSO, MR-Pleiotropy RESidual Sum and Outlier

Table S11. Age- and sex-specific subgroup analyses for phenotypic associations between HDL-C concentration and the risk of DSCs

Outcomes	Group	HDL-C: <1.0mmol/L			HDL-C: 1.0-1.6mmol/L		HDL-C: ≥1.6mmol/L			P_{trend}[b]	$P_{interaction}$[c]
		No.(Cases)	HR(95% CI)	*P* value[a]	No.(Cases)	HR(95% CI)	No.(Cases)	HR(95% CI)	*P* value[a]		
**Oesophagus (C15)**	**Age**										
	<60 years	16069(55)	1.210(0.881-1.663)	0.239	106592(185)	ref	55784(48)	0.840(0.597-1.182)	0.317	0.648	**0.006**
	≥60 years	12814(98)	1.181(0.935-1.490)	0.162	82770(369)	ref	45539(146)	1.104(0.897-1.358)	0.351	0.199	
	**Sex**										
	Female	4912(11)	1.302(0.693-2.447)	0.412	87909(123)	ref	80252(114)	1.128(0.860-1.481)	0.384	0.350	0.398
	Male	23971(142)	1.184(0.971-1.444)	0.094	101453(431)	ref	21071(80)	0.916(0.718-1.170)	0.482	0.929	
**Stomach (C16)**	**Age**										
	<60 years	16069(32)	0.947(0.633-1.417)	0.792	106592(141)	ref	55784(37)	0.795(0.537-1.175)	0.249	0.255	0.684
	≥60 years	12814(87)	**1.424(1.107-1.833)**	**0.006**	82770(294)	ref	45539(78)	**0.683(0.523-0.892)**	**0.005**	0.099	
	**Sex**										
	Female	4912(15)	**2.062(1.181-3.063)**	**0.011**	87909(111)	ref	80252(77)	0.857(0.630-1.165)	0.324	0.492	0.408
	Male	23971(104)	1.190(0.945-1.499)	0.140	101453(324)	ref	21071(38)	**0.576(0.409-0.810)**	**0.002**	**0.036**	
**Colorectal (C18-C20)**	**Age**										
	<60 years	16069(158)	0.913(0.766-1.089)	0.312	106592(907)	ref	55784(448)	**1.138(1.004-1.290)**	**0.043**	0.106	0.753
	≥60 years	12814(327)	1.031(0.912-1.166)	0.624	82770(1732)	ref	45539(838)	1.055(0.964-1.154)	0.248	0.226	
	**Sex**										
	Female	4912(52)	0.866(0.653-1.149)	0.318	87909(1016)	ref	80252(921)	1.036(0.942-1.139)	0.469	0.522	0.478
	Male	23971(433)	1.021(0.915-1.140)	0.707	101453(1623)	ref	21071(365)	1.098(0.977-1.233)	0.118	0.131	
**Liver (C22)**	**Age**										
	<60 years	16069(9)	0.906(0.432-1.900)	0.794	106592(54)	ref	55784(24)	0.885(0.524-1.496)	0.648	0.618	0.443
	≥60 years	12814(36)	1.083(0.739-1.587)	0.683	82770(152)	ref	45539(61)	0.951(0.688-1.314)	0.760	0.873	
	**Sex**										
	Female	4912(9)	1.398(0.690-2.832)	0.352	87909(90)	ref	80252(59)	0.813(0.573-1.151)	0.243	0.298	0.363
	Male	23971(36)	0.988(0.669-1.459)	0.953	101453(116)	ref	21071(26)	1.110(0.715-1.723)	0.641	0.709	
**Gallbladder (C23-C24)**	**Age**										
	<60 years	16069(4)	0.561(0.195-1.614)	0.284	106592(41)	ref	55784(21)	1.078(0.605-1.923)	0.798	0.977	0.420
	≥60 years	12814(16)	0.795(0.458-1.377)	0.413	82770(152)	ref	45539(45)	0.954(0.649-1.401)	0.809	0.648	
	**Sex**										
	Female	4912(3)	0.552(0.172-1.775)	0.319	87909(73)	ref	80252(50)	0.884(0.602-1.297)	0.528	0.464	0.566
	Male	23971(17)	0.792(0.456-1.378)	0.410	101453(69)	ref	21071(16)	1.225(0.699-2.148)	0.478	0.789	
**Pancreas (C25)**	**Age**										
	<60 years	16069(35)	0.975(0.662-1.437)	0.899	106592(154)	ref	55784(72)	1.163(0.855-1.584)	0.337	0.396	0.906
	≥60 years	12814(73)	1.005(0.773-1.306)	0.971	82770(152)	ref	45539(190)	1.119(0.923-1.357)	0.253	0.280	
	**Sex**										
	Female	4912(16)	1.050(0.626-1.761)	0.854	87909(206)	ref	80252(114)	1.197(0.970-1.477)	0.093	0.095	0.083
	Male	23971(92)	1.034(0.811-1.317)	0.789	101453(317)	ref	21071(68)	1.041(0.795-1.363)	0.768	0.717	

[a, b and c] P, P_{trend} and $P_{interaction}$ values of 0.008 (0.05/6 outcomes, Bonferroni-adjusted P) were defined as the thresholds for remarkable statistical significances. P_{trend} and $P_{interaction}$ values between 0.008 and 0.05 were defined as suggestive statistical significances.

With the exception of subgroup covariates, all subgroup analyses were adjusted with age or sex, BMI, smoking status, alcohol drinking status, education qualification, employment status, TDI, physical activity level, family cancer history, ALT, AST, SBP, DBP, cerebral infarction, ischaemic heart disease, primary hypertension, HbA1c, and diabetes.

BMI, body mass index; TDI, Townsend deprivation index; ALT, alanine aminotransferase; AST, aspartate aminotransferase; SBP, systolic blood pressure; DBP, diastolic blood pressure HbA1c, glycated haemoglobin; HDL-C, high-density lipoprotein cholesterol; DSC, digestive system cancer

Table S12. Age- and sex-specific subgroup analyses for phenotypic associations between LDL-C concentration and the risk of DSCs

Outcomes	Group	LDL-C: <3.4mmol/L			LDL -C: 3.4-4.1mmol/L		LDL -C: ≥4.1mmol/L			P_{trend}[b]	$P_{interaction}$[c]
		No.(Cases)	HR(95% CI)	*P* value[a]	No.(Cases)	HR(95% CI)	No.(Cases)	HR(95% CI)	*P* value[a]		
**Oesophagus (C15)**	**Age**										
	<60 years	78500(122)	**0.762(0.585-0.992)**	**0.044**	55200(108)	ref	44745(58)	**0.623(0.452-0.858)**	**0.004**	**0.003**	0.066
	≥60 years	61207(321)	1.048(0.862-1.273)	0.639	40818(163)	ref	39098(129)	0.923(0.732-1.164)	0.503	0.557	
	**Sex**										
	Female	71753(101)	1.053(0.769-1.441)	0.748	52390(68)	ref	48930(79)	1.113(0.804-1.541)	0.520	0.520	0.997
	Male	67954(342)	0.877(0.733-1.050)	0.153	43628(203)	ref	34913(108)	**0.688(0.544-0.869)**	**0.002**	**0.004**	
**Stomach (C16)**	**Age**										
	<60 years	78500(91)	1.071(0.854-1.342)	0.554	55200(77)	ref	44745(42)	0.935(0.715-1.223)	0.622	0.685	0.405
	≥60 years	61207(241)	0.810(0.594-1.103)	0.181	40818(121)	ref	39098(97)	**0.615(0.422-0.898)**	**0.012**	**0.011**	
	**Sex**										
	Female	71753(80)	0.956(0.771-1.186)	0.685	52390(61)	ref	48930(62)	**0.726(0.548-0.960)**	**0.025**	**0.035**	0.414
	Male	67954(252)	0.964(0.686-1.354)	0.834	43628(137)	ref	34913(77)	0.990(0.694-1.411)	0.954	0.953	
**Colorectal (C18-C20)**	**Age**										
	<60 years	78500(629)	0.913(0.809-1.030)	0.140	55200(469)	ref	44745(415)	1.070(0.937-1.223)	0.315	0.394	**0.012**
	≥60 years	61207(1316)	**0.910(0.832-0.996)**	**0.041**	40818(834)	ref	39098(747)	0.999(0.904-1.103)	0.978	0.866	
	**Sex**										
	Female	71753(745)	0.899(0.807-1.001)	0.053	52390(625)	ref	48930(619)	0.978(0.875-1.094)	0.701	0.679	0.078
	Male	67954(1200)	0.926(0.841-1.021)	0.123	43628(678)	ref	34913(543)	1.067(0.953-1.195)	0.262	0.389	
**Liver (C22)**	**Age**										
	<60 years	78500(35)	0.845(0.513-1.391)	0.507	55200(29)	ref	44745(23)	0.892(0.514-1.545)	0.683	0.657	**<0.001**
	≥60 years	61207(137)	1.225(0.894-1.678)	0.207	40818(60)	ref	39098(52)	0.949(0.653-1.379)	0.784	0.869	
	**Sex**										
	Female	71753(59)	0.854(0.582-1.252)	0.418	52390(51)	ref	48930(48)	0.873(0.589-1.297)	0.501	0.498	0.141
	Male	67954(113)	1.384(0.947-2.024)	0.094	43628(38)	ref	34913(27)	0.963(0.587-1.581)	0.882	0.904	
**Gallbladder (C23-C24)**	**Age**										
	<60 years	78500(23)	0.640(0.361-1.136)	0.128	55200(25)	ref	44745(18)	0.840(0.456-1.545)	0.574	0.490	0.914
	≥60 years	61207(68)	0.930(0.618-1.400)	0.728	40818(39)	ref	39098(55)	**1.541(1.020-2.328)**	**0.040**	**0.037**	
	**Sex**										
	Female	71753(43)	0.767(0.494-1.191)	0.238	52390(40)	ref	48930(43)	1.037(0.673-1.598)	0.698	0.863	0.706
	Male	67954(48)	0.904(0.544-1.503)	0.869	43628(24)	ref	34913(30)	**1.761(1.027-3.019)**	**0.040**	**0.046**	
**Pancreas (C25)**	**Age**										
	<60 years	78500(113)	0.846(0.634-1.130)	0.257	55200(83)	ref	44745(65)	0.920(0.664-1.274)	0.615	0.560	0.525
	≥60 years	61207(299)	1.125(0.918-1.378)	0.256	40818(150)	ref	39098(183)	**1.341(1.079-1.665)**	**0.008**	**0.009**	
	**Sex**										
	Female	71753(161)	0.993(0.778-1.267)	0.954	52390(115)	ref	48930(140)	1.177(0.919-1.508)	0.196	0.188	0.500
	Male	67954(251)	1.046(0.834-1.312)	0.696	43628(118)	ref	34913(108)	1.211(0.932-1.573)	0.152	0.164	

[a, b and c] *P*, P_{trend} and $P_{interaction}$ values of 0.008 (0.05/6 outcomes, Bonferroni-adjusted *P*) were defined as the thresholds for remarkable statistical significances. P_{trend} and $P_{interaction}$ values between 0.008 and 0.05 were defined as suggestive statistical significances.

With the exception of subgroup covariates, all subgroup analyses were adjusted with age or sex, BMI, smoking status, alcohol drinking status, education qualification, employment status, TDI, physical activity level, family cancer history, ALT, AST, SBP, DBP, cerebral infarction, ischaemic heart disease, primary hypertension, HbA1c, and diabetes.

BMI, body mass index; TDI, Townsend deprivation index; ALT, alanine aminotransferase; AST, aspartate aminotransferase; SBP, systolic blood pressure; DBP, diastolic blood pressure HbA1c, glycated haemoglobin; LDL-C, low-density lipoprotein cholesterol; DSC, digestive system cancer

Table S13. Age- and sex-specific subgroup analyses for phenotypic associations between TG concentration and the risk of DSCs

Outcomes	Group	TG: <1.7mmol/L		TG: 1.7-2.2mmol/L			TG: ≥2.2mmol/L			P_{trend}[b]	$P_{interaction}$[c]
		No.(Cases)	HR(95% CI)	No.(Cases)	HR(95% CI)	*P* value[a]	No.(Cases)	HR(95% CI)	*P* value[a]		
**Oesophagus (C15)**	**Age**										
	<60 years	112256(145)	ref	26417(40)	0.809(0.567-1.156)	0.245	39772(103)	1.136(0.863-1.495)	0.365	0.420	0.637
	≥60 years	79874(302)	ref	26159(132)	1.186(0.964-1.459)	0.107	35090(179)	1.054(0.869-1.278)	0.594	0.486	
	**Sex**										
	Female	118366(150)	ref	25899(50)	1.184(0.853-1.644)	0.314	28808(48)	0.920(0.653-1.296)	0.634	0.824	0.156
	Male	73764(297)	ref	26677(122)	1.022(0.826-1.265)	0.839	46054(234)	1.140(0.953-1.363)	0.153	0.161	
**Stomach (C16)**	**Age**										
	<60 years	112256(104)	ref	26417(39)	1.134(0.777-1.654)	0.515	39772(67)	1.051(0.752-1.469)	0.770	0.738	0.672
	≥60 years	79874(234)	ref	26159(85)	0.987(0.767-1.269)	0.918	35090(140)	1.080(0.867-1.345)	0.495	0.522	
	**Sex**										
	Female	118366(119)	ref	25899(43)	1.283(0.896-1.836)	0.174	28808(41)	1.002(0.688-1.458)	0.992	0.776	0.492
	Male	73764(219)	ref	26677(81)	0.926(0.715-1.198)	0.557	46054(166)	1.102(0.892-1.361)	0.369	0.397	
**Colorectal (C18-C20)**	**Age**										
	<60 years	112256(874)	ref	26417(248)	1.054(0.912-1.219)	0.475	39772(391)	0.997(0.874-1.137)	0.959	0.962	0.253
	≥60 years	79874(1540)	ref	26159(562)	1.069(0.969-1.179)	0.184	35090(795)	1.086(0.992-1.188)	0.074	0.061	
	**Sex**										
	Female	118366(1242)	ref	25899(335)	1.039(0.918-1.176)	0.546	28808(412)	1.101(0.978-1.239)	0.112	0.113	0.903
	Male	73764(1172)	ref	26677(475)	1.057(0.949-1.178)	0.311	46054(774)	1.029(0.935-1.132)	0.560	0.514	
**Liver (C22)**	**Age**										
	<60 years	112256(52)	ref	26417(11)	0.758(0.390-1.475)	0.415	39772(24)	0.952(0.561-1.617)	0.857	0.783	0.057
	≥60 years	79874(124)	ref	26159(46)	0.987(0.700-1.392)	0.942	35090(79)	1.112(0.827-1.495)	0.482	0.503	
	**Sex**										
	Female	118366(86)	ref	25899(31)	1.201(0.789-1.829)	0.394	28808(41)	1.257(0.847-1.867)	0.257	0.232	**0.011**
	Male	73764(90)	ref	26677(26)	0.714(0.460-1.109)	0.134	46054(62)	0.956(0.682-1.339)	0.793	0.717	
**Gallbladder (C23-C24)**	**Age**										
	<60 years	112256(38)	ref	26417(11)	1.079(0.541-2.152)	0.829	39772(17)	1.058(0.564-1.985)	0.860	0.843	0.589
	≥60 years	79874(83)	ref	26159(25)	0.865(0.550-1.361)	0.531	35090(54)	**1.331(1.009-1.757)**	**0.043**	0.083	
	**Sex**										
	Female	118366(77)	ref	25899(20)	0.876(0.530-1.449)	0.607	28808(29)	1.003(0.637-1.579)	0.990	0.933	0.186
	Male	73764(44)	ref	26677(16)	0.983(0.551-1.752)	0.953	46054(42)	**1.589(1.017-2.482)**	**0.042**	**0.038**	
**Pancreas (C25)**	**Age**										
	<60 years	112256(137)	ref	26417(44)	1.107(0.780-1.570)	0.569	39772(80)	1.131(0.833-1.537)	0.430	0.416	0.367
	≥60 years	79874(343)	ref	26159(104)	0.872(0.698-1.089)	0.227	35090(185)	1.066(0.882-1.288)	0.509	0.640	
	**Sex**										
	Female	118366(259)	ref	25899(62)	0.820(0.618-1.089)	0.170	28808(95)	1.001(0.779-1.285)	0.996	0.797	0.473
	Male	73764(221)	ref	26677(86)	1.015(0.789-1.306)	0.910	46054(170)	1.176(0.953-1.452)	0.131	0.140	

[a, b and c] *P*, P_{trend} and $P_{interaction}$ values of 0.008 (0.05/6 outcomes, Bonferroni-adjusted *P*) were defined as the thresholds for remarkable statistical significances. P_{trend} and $P_{interaction}$ values between 0.008 and 0.05 were defined as suggestive statistical significances.

With the exception of subgroup covariates, all subgroup analyses were adjusted with age or sex, BMI, smoking status, alcohol drinking status, education qualification, employment status, TDI, physical activity level, family cancer history, ALT, AST, SBP, DBP, cerebral infarction, ischaemic heart disease, primary hypertension, HbA1c, and diabetes.

BMI, body mass index; TDI, Townsend deprivation index; ALT, alanine aminotransferase; AST, aspartate aminotransferase; SBP, systolic blood pressure; DBP, diastolic blood pressure HbA1c, glycated haemoglobin; TG, triglycerides; DSC, digestive system cancer

Table S14. Age- and sex-specific linear MR analyses

Outcomes	Group	No.(Cases)	HDL-C (mmol/L)		LDL-C (mmol/L)		TG (mmol/L)	
			HR(95% CI)[a]	*P* value[b]	HR(95% CI)[a]	*P* value[b]	HR(95% CI)[a]	*P* value[b]
**Oesophagus (C15)**	**Age**							
	<60 years	178445(288)	1.456(0.376-5.639)	0.586	2.504(0.741-8.456)	0.139	1.065(0.210-5.394)	0.139
	≥60 years	141123(613)	0.779(0.301-2.014)	0.606	0.753(0.326-1.743)	0.508	0.550(0.194-1.555)	0.260
	**Sex**							
	Female	173073(248)	1.054(0.261-4.253)	0.941	1.562(0.386-6.328)	0.532	2.606(0.368-18.445)	0.337
	Male	146495(653)	0.922(0.364-2.336)	0.865	0.992(0.811-1.214)	0.938	0.472(0.194-1.149)	0.098
**Stomach (C16)**	**Age**							
	<60 years	178445(210)	0.228(0.048-1.087)	0.063	0.968(0.229-4.085)	0.965	0.595(0.089-3.991)	0.593
	≥60 years	141123(459)	1.119(0.372-3.364)	0.063	**0.264(0.103-0.677)**	**0.006**	0.734(0.221-2.441)	0.614
	**Sex**							
	Female	173073(203)	0.998(0.214-4.655)	0.998	**0.601(0.396-0.913)**	**0.017**	0.746(0.086-6.502)	0.791
	Male	146495(466)	0.557(0.186-1.664)	0.295	0.656(0.238-1.809)	0.416	0.683(0.238-1.960)	0.479
**Colorectal (C18-C20)**	**Age**							
	<60 years	178445(1513)	1.409(0.780-2.543)	0.256	0.917(0.542-1.550)	0.745	1.057(0.520-2.145)	0.879
	≥60 years	141123(2897)	0.921(0.594-1.427)	0.712	1.106(0.752-1.628)	0.609	1.177(0.730-1.899)	0.503
	**Sex**							
	Female	173073(1989)	1.053(0.644-1.724)	0.837	0.867(0.530-1.417)	0.569	0.992(0.497-1.980)	0.982
	Male	146495(2421)	1.078(0.665-1.748)	0.761	1.043(0.939-1.158)	0.437	1.213(0.764-1.925)	0.413
**Liver (C22)**	**Age**							
	<60 years	178445(87)	0.104(0.009-1.154)	0.065	3.157(0.572-17.441)	0.586	5.832(0.892-38.125)	0.066
	≥60 years	141123(249)	0.799(0.180-3.559)	0.769	0.175(0.029-1.070)	0.059	2.963(0.581-15.121)	0.191
	**Sex**							
	Female	173073(158)	0.215(0.032-1.419)	0.110	0.235(0.042-1.301)	0.097	6.375(0.702-57.877)	0.100
	Male	146495(178)	0.964(0.163-5.711)	0.968	0.920(0.626-1.353)	0.673	1.128(0.205-6.195)	0.890
**Gallbladder (C23-C24)**	**Age**							
	<60 years	178445(66)	2.557(0.493-13.253)	0.264	0.112(0.010-1.306)	0.081	5.303(0.524-53.629)	0.158
	≥60 years	141123(162)	1.114(0.174-7.148)	0.909	2.422(0.468-12.548)	0.292	2.485(0.599-10.306)	0.210
	**Sex**							
	Female	173073(126)	0.727(0.103-5.109)	0.749	0.656(0.093-4.601)	0.671	5.245(0.339-81.235)	0.236
	Male	146495(102)	2.092(0.676-6.473)	0.201	1.112(0.666-1.857)	0.685	3.160(0.494-20.228)	0.225
**Pancreas (C25)**	**Age**							
	<60 years	178445(261)	1.142(0.276-4.772)	0.855	2.534(0.707-9.084)	0.153	1.135(0.239-7.227)	0.753
	≥60 years	141123(632)	0.979(0.383-2.504)	0.964	0.762(0.334-1.739)	0.519	2.214(0.796-6.157)	0.128
	**Sex**							
	Female	173073(416)	2.006(0.679-5.924)	0.208	1.028(0.350-3.017)	0.960	2.175(0.480-9.853)	0.313
	Male	146495(477)	0.546(0.185-1.611)	0.273	1.030(0.814-1.305)	0.804	1.693(0.598-4.793)	0.321

[a] HR per 1 mmol/L increase of genetically predicted signature lipidomic biomarkers.

[b] *P* value of 0.008 (0.05/6 outcomes, Bonferroni-adjusted *P*) was defined as the threshold for remarkable statistical significance. *P* value between 0.008 and 0.05 was defined as suggestive statistical significance.

HDL-C, high-density lipoprotein cholesterol; LDL-C, low-density lipoprotein cholesterol; TG, triglycerides; HR, hazard ratio

Table S15. Age- and sex-specific stratified MR analyses for the associations between three categories of HDL-C concentration and the risk of DSCs

Outcomes	Group	HDL-C: <1.0 mmol/L			HDL-C: 1.0-1.6 mmol/L			HDL-C: ≥1.6 mmol/L		
		No.(Cases)	HR(95% CI)[a]	*P* value[b]	No.(Cases)	HR(95% CI)[a]	*P* value[b]	No.(Cases)	HR(95% CI)[a]	*P* value[b]
**Oesophagus (C15)**	**Age**									
	<60 years	16069(55)	1.172(0.862-1.593)	0.310	106592(185)	1.031(0.870-1.222)	0.726	55784(48)	1.111(0.791-1.559)	0.544
	≥60 years	12814(98)	1.029(0.814-1.301)	0.812	82770(369)	0.925(0.818-1.045)	0.211	45539(146)	1.134(0.928-1.386)	0.219
	**Sex**									
	Female	4912(11)	1.446(0.745-2.806)	0.275	87909(123)	0.842(0.694-1.022)	0.083	80252(114)	1.200(0.971-1.483)	0.091
	Male	23971(142)	1.049(0.861-1.277)	0.638	101453(431)	1.003(0.893-1.125)	0.966	21071(80)	1.014(0.773-1.331)	0.918
**Stomach (C16)**	**Age**									
	<60 years	16069(32)	0.787(0.532-1.163)	0.229	106592(141)	0.831(0.687-1.006)	0.058	55784(37)	1.238(0.838-1.829)	0.284
	≥60 years	12814(87)	1.126(0.876-1.446)	0.356	82770(294)	1.032(0.899-1.186)	0.651	45539(78)	1.030(0.784-1.354)	0.831
	**Sex**									
	Female	4912(15)	0.999(0.571-1.748)	0.997	87909(111)	0.968(0.787-1.191)	0.759	80252(114)	1.147(0.887-1.482)	0.759
	Male	23971(104)	1.026(0.815-1.291)	0.827	101453(324)	0.958(0.839-1.094)	0.528	21071(38)	0.970(0.655-1.436)	0.880
**Colorectal (C18-C20)**	**Age**									
	<60 years	16069(158)	1.107(0.924-1.324)	0.270	106592(907)	1.021(0.946-1.102)	0.597	55784(448)	1.038(0.929-1.159)	0.511
	≥60 years	12814(327)	1.009(0.888-1.148)	0.886	82770(1732)	1.008(0.952-1.067)	0.778	45539(838)	0.961(0.885-1.045)	0.354
	**Sex**									
	Female	4912(52)	1.025(0.765-1.375)	0.867	87909(1016)	0.999(0.933-1.070)	0.976	80252(114)	1.014(0.942-1.092)	0.710
	Male	23971(433)	1.040(0.928-1.164)	0.500	101453(1623)	1.024(0.965-1.087)	0.432	21071(365)	0.926(0.816-1.051)	0.233
**Liver (C22)**	**Age**									
	<60 years	16069(9)	0.795(0.390-1.621)	0.527	106592(54)	0.862(0.633-1.173)	0.346	55784(24)	0.688(0.432-1.095)	0.115
	≥60 years	12814(36)	1.226(0.829-1.814)	0.308	82770(152)	0.940(0.777-1.139)	0.528	45539(61)	1.034(0.759-1.409)	0.831
	**Sex**									
	Female	4912(9)	0.909(0.453-1.825)	0.788	87909(90)	0.883(0.703-1.109)	0.285	80252(114)	0.881(0.659-1.176)	0.389
	Male	23971(36)	1.177(0.793-1.745)	0.419	101453(116)	0.950(0.761-1.185)	0.648	21071(26)	1.035(0.644-1.665)	0.886
**Gallbladder (C23-C24)**	**Age**									
	<60 years	16069(4)	1.760(0.902-3.437)	0.098	106592(41)	1.233(0.856-1.775)	0.261	55784(21)	0.669(0.408-1.095)	0.110
	≥60 years	12814(16)	1.140(0.632-2.056)	0.662	82770(152)	1.018(0.804-1.290)	0.881	45539(45)	1.003(0.699-1.440)	0.987
	**Sex**									
	Female	4912(3)	2.112(0.565-7.894)	0.266	87909(73)	1.052(0.814-1.361)	0.697	80252(114)	0.852(0.623-1.164)	0.314
	Male	23971(17)	1.423(0.792-2.557)	0.238	101453(69)	1.100(0.822-1.472)	0.521	21071(16)	0.984(0.540-1.791)	0.957
**Pancreas (C25)**	**Age**									
	<60 years	16069(35)	1.396(0.841-2.318)	0.197	106592(154)	1.046(0.868-1.261)	0.635	55784(72)	0.809(0.617-1.060)	0.124
	≥60 years	12814(73)	0.896(0.686-1.171)	0.423	82770(152)	1.021(0.902-1.155)	0.748	45539(190)	1.015(0.852-1.210)	0.865
	**Sex**									
	Female	4912(16)	0.967(0.568-1.646)	0.901	87909(206)	1.164(0.997-1.359)	0.054	80252(114)	0.999(0.851-1.174)	0.995
	Male	23971(92)	1.077(0.843-1.377)	0.552	101453(317)	0.943(0.825-1.079)	0.393	21071(68)	0.828(0.620-1.105)	0.201

[a] HR per 0.1 mmol/L increase of genetically predicted signature lipidomic biomarkers.

[b] *P* value of 0.008 (0.05/6 outcomes, Bonferroni-adjusted *P*) was defined as the threshold for remarkable statistical significance. *P* value between 0.008 and 0.05 was defined as suggestive statistical significance.

HDL-C, high-density lipoprotein cholesterol; HR, hazard ratio

Table S16. Age- and sex-specific stratified MR analyses for the associations between three categories of LDL-C concentration and the risk of DSCs

Outcomes	Group	LDL-C: < 3.4 mmol/L			LDL-C: 3.4-4.1 mmol/L			LDL-C: ≥ 4.1 mmol/L		
		No.(Cases)	HR(95% CI)[a]	*P* value[b]	No.(Cases)	HR(95% CI)[a]	*P* value[b]	No.(Cases)	HR(95% CI)[a]	*P* value[b]
**Oesophagus (C15)**	**Age**									
	<60 years	78500(122)	1.047(0.871-1.260)	0.623	55200(108)	1.145(0.935-1.401)	0.190	44745(58)	1.170(0.886-1.543)	0.269
	≥60 years	61207(321)	1.009(0.899-1.134)	0.873	40818(163)	1.048(0.890-1.234)	0.576	39098(129)	0.832(0.676-1.025)	0.844
	**Sex**									
	Female	71753(101)	1.117(0.912-1.368)	0.285	52390(68)	0.942(0.735-1.207)	0.637	48930(79)	1.040(0.825-1.312)	0.739
	Male	67954(342)	0.981(0.854-1.128)	0.790	43628(203)	1.184(0.985-1.422)	0.072	34913(108)	0.813(0.634-1.042)	0.102
**Stomach (C16)**	**Age**									
	<60 years	78500(91)	0.899(0.790-1.024)	0.109	55200(77)	0.922(0.766-1.109)	0.388	39098(97)	0.890(0.771-1.029)	0.114
	≥60 years	61207(241)	0.830(0.670-1.028)	0.088	40818(121)	1.277(0.999-1.631)	0.050	44745(42)	**0.786(0.638-0.969)**	**0.024**
	**Sex**									
	Female	71753(80)	0.780(0.604-1.008)	0.057	52390(61)	0.921(0.691-1.227)	0.574	48930(62)	**0.789(0.637-0.977)**	**0.030**
	Male	67954(252)	0.950(0.711-1.269)	0.727	43628(137)	1.126(0.805-1.574)	0.488	34913(77)	0.917(0.682-1.232)	0.565
**Colorectal (C18-C20)**	**Age**									
	<60 years	78500(629)	1.001(0.923-1.085)	0.989	55200(469)	1.009(0.917-1.110)	0.861	44745(415)	0.938(0.847-1.038)	0.218
	≥60 years	61207(1316)	1.008(0.952-1.068)	0.774	40818(834)	0.986(0.917-1.060)	0.705	39098(747)	1.047(0.969-1.131)	0.245
	**Sex**									
	Female	71753(745)	0.981(0.911-1.057)	0.616	52390(625)	1.016(0.936-1.103)	0.705	48930(619)	0.961(0.885-1.044)	0.346
	Male	67954(1200)	1.019(0.946-1.097)	0.621	43628(678)	0.977(0.885-1.079)	0.650	34913(543)	1.082(0.966-1.211)	0.172
**Liver (C22)**	**Age**									
	<60 years	78500(35)	1.241(0.874-1.762)	0.228	55200(29)	0.847(0.581-1.235)	0.389	44745(23)	1.429(0.916-2.230)	0.116
	≥60 years	61207(137)	0.825(0.594-1.146)	0.252	40818(60)	0.948(0.725-1.239)	0.695	39098(52)	0.808(0.577-1.132)	0.215
	**Sex**									
	Female	71753(59)	0.765(0.580-1.008)	0.057	52390(51)	0.915(0.672-1.247)	0.574	48930(48)	0.785(0.574-1.074)	0.130
	Male	67954(113)	0.930(0.731-1.183)	0.554	43628(38)	0.903(0.594-1.372)	0.633	34913(27)	1.371(0.824-2.281)	0.224
**Gallbladder (C23-C24)**	**Age**									
	<60 years	78500(23)	0.761(0.504-1.149)	0.194	55200(25)	0.652(0.409-1.041)	0.073	44745(18)	1.186(0.723-1.946)	0.498
	≥60 years	61207(68)	1.088(0.845-1.401)	0.514	40818(39)	1.384(0.983-1.949)	0.063	39098(55)	0.909(0.686-1.204)	0.506
	**Sex**									
	Female	71753(43)	0.994(0.713-1.384)	0.969	52390(40)	0.909(0.641-1.289)	0.593	48930(43)	0.953(0.681-1.334)	0.780
	Male	67954(48)	0.978(0.674-1.418)	0.906	43628(24)	1.214(0.710-2.075)	0.478	34913(30)	0.997(0.618-1.608)	0.990
**Pancreas (C25)**	**Age**									
	<60 years	78500(113)	1.102(0.909-1.337)	0.322	55200(83)	1.051(0.836-1.320)	0.670	44745(65)	1.155(0.890-1.500)	0.277
	≥60 years	61207(299)	0.990(0.878-1.116)	0.872	40818(150)	0.977(0.824-1.158)	0.790	39098(183)	0.935(0.801-1.092)	0.396
	**Sex**									
	Female	71753(161)	1.030(0.867-1.225)	0.733	52390(115)	0.926(0.754-1.138)	0.466	48930(140)	1.030(0.854-1.243)	0.757
	Male	67954(251)	1.010(0.859-1.188)	0.905	43628(118)	1.115(0.877-1.418)	0.374	34913(108)	0.927(0.721-1.191)	0.553

[a]HR per 0.1 mmol/L increase of genetically predicted signature lipidomic biomarkers.

[b] *P* value of 0.008 (0.05/6 outcomes, Bonferroni-adjusted *P*) was defined as the threshold for remarkable statistical significance. *P* value between 0.008 and 0.05 was defined as suggestive statistical significance.

LDL-C, low-density lipoprotein cholesterol; HR, hazard ratio

Table S17. Age- and sex-specific stratified MR analyses for the associations between three categories of TG concentration and the risk of DSCs

Outcomes	Group	TG: < 1.7 mmol/L			TG: 1.7-2.2 mmol/L			TG: ≥ 2.2 mmol/L		
		No.(Cases)	HR(95% CI)[a]	*P* value[b]	No.(Cases)	HR(95% CI)[a]	*P* value[b]	No.(Cases)	HR(95% CI)[a]	*P* value[b]
**Oesophagus (C15)**	**Age**									
	<60 years	112256(145)	0.903(0.719-1.135)	0.382	26417(40)	1.160(0.748-1.799)	0.508	39772(103)	1.067(0.812-1.401)	0.643
	≥60 years	79874(302)	0.933(0.804-1.083)	0.364	26159(132)	0.954(0.762-1.193)	0.678	35090(179)	0.920(0.758-1.117)	0.399
	**Sex**									
	Female	118366(150)	1.069(0.831-1.375)	0.603	25899(50)	0.868(0.557-1.352)	0.530	28808(48)	1.490(0.957-2.319)	0.078
	Male	73764(297)	0.880(0.771-1.005)	0.058	26677(122)	1.031(0.841-1.265)	0.767	46054(234)	0.906(0.780-1.052)	0.196
**Stomach (C16)**	**Age**									
	<60 years	112256(104)	1.040(0.795-1.362)	0.774	26417(39)	1.057(0.676-1.652)	0.808	39772(67)	0.741(0.527-1.041)	0.084
	≥60 years	79874(234)	0.929(0.785-1.100)	0.393	26159(85)	1.040(0.787-1.374)	0.784	35090(140)	0.968(0.778-1.205)	0.773
	**Sex**									
	Female	118366(119)	0.916(0.691-1.216)	0.546	25899(43)	1.125(0.700-1.810)	0.626	28808(41)	0.922(0.570-1.491)	0.739
	Male	73764(219)	0.981(0.841-1.144)	0.804	26677(81)	1.103(0.788-1.304)	0.918	46054(166)	0.893(0.748-1.067)	0.214
**Colorectal (C18-C20)**	**Age**									
	<60 years	112256(874)	1.001(0.912-1.098)	0.988	26417(248)	0.922(0.772-1.100)	0.366	39772(391)	1.049(0.912-1.206)	0.505
	≥60 years	79874(1540)	0.977(0.915-1.044)	0.492	26159(562)	1.030(0.924-1.149)	0.590	35090(795)	1.068(0.974-1.170)	0.160
	**Sex**									
	Female	118366(1242)	0.981(0.899-1.071)	0.667	25899(335)	1.030(0.869-1.222)	0.730	28808(412)	0.995(0.855-1.159)	0.952
	Male	73764(1172)	0.988(0.924-1.056)	0.726	26677(475)	0.980(0.883-1.087)	0.700	46054(774)	1.082(0.997-1.174)	0.060
**Liver (C22)**	**Age**									
	<60 years	112256(52)	1.369(0.936-2.001)	0.105	26417(11)	0.614(0.264-1.429)	0.258	39772(24)	1.373(0.785-2.400)	0.266
	≥60 years	79874(124)	1.129(0.895-1.424)	0.307	26159(46)	0.860(0.588-1.259)	0.439	35090(79)	1.228(0.919-1.641)	0.165
	**Sex**									
	Female	118366(86)	1.269(0.912-1.767)	0.158	25899(31)	0.843(0.480-1.479)	0.551	28808(41)	1.200(0.741-1.941)	0.459
	Male	73764(90)	0.980(0.771-1.247)	0.872	26677(26)	0.777(0.499-1.212)	0.266	46054(62)	1.160(0.869-1.549)	0.313
**Gallbladder (C23-C24)**	**Age**									
	<60 years	112256(38)	1.362(0.873-2.124)	0.173	26417(11)	0.771(0.335-1.773)	0.540	39772(17)	1.553(0.801-3.011)	0.192
	≥60 years	79874(83)	0.955(0.749-1.321)	0.970	26159(25)	1.067(0.635-1.794)	0.807	35090(54)	**1.264(1.003-1.593)**	**0.047**
	**Sex**									
	Female	118366(77)	1.195(0.842-1.697)	0.319	25899(20)	1.329(0.663-2.662)	0.423	28808(29)	1.012(0.569-1.799)	0.968
	Male	73764(44)	0.983(0.696-1.388)	0.921	26677(16)	0.737(0.417-1.304)	0.295	46054(42)	**1.447(1.020-2.052)**	**0.038**
**Pancreas (C25)**	**Age**									
	<60 years	112256(137)	1.012(0.800-1.281)	0.919	26417(44)	1.283(0.844-1.951)	0.244	39772(80)	0.881(0.646-1.201)	0.421
	≥60 years	79874(343)	1.092(0.950-1.255)	0.216	26159(104)	1.041(0.809-1.339)	0.757	35090(185)	1.065(0.881-1.288)	0.515
	**Sex**									
	Female	118366(259)	1.128(0.931-1.365)	0.218	25899(62)	1.014(0.681-1.508)	0.947	28808(95)	0.961(0.699-1.320)	0.805
	Male	73764(221)	1.019(0.874-1.188)	0.808	26677(86)	1.146(0.899-1.463)	0.272	46054(170)	1.024(0.859-1.220)	0.795

[a] HR per 0.1 mmol/L increase of genetically predicted signature lipidomic biomarkers.

[b] *P* value of 0.008 (0.05/6 outcomes, Bonferroni-adjusted *P*) was defined as the threshold for remarkable statistical significance. *P* value between 0.008 and 0.05 was defined as suggestive statistical significance.

TG, triglycerides; HR, hazard ratio

Supplementary Figures

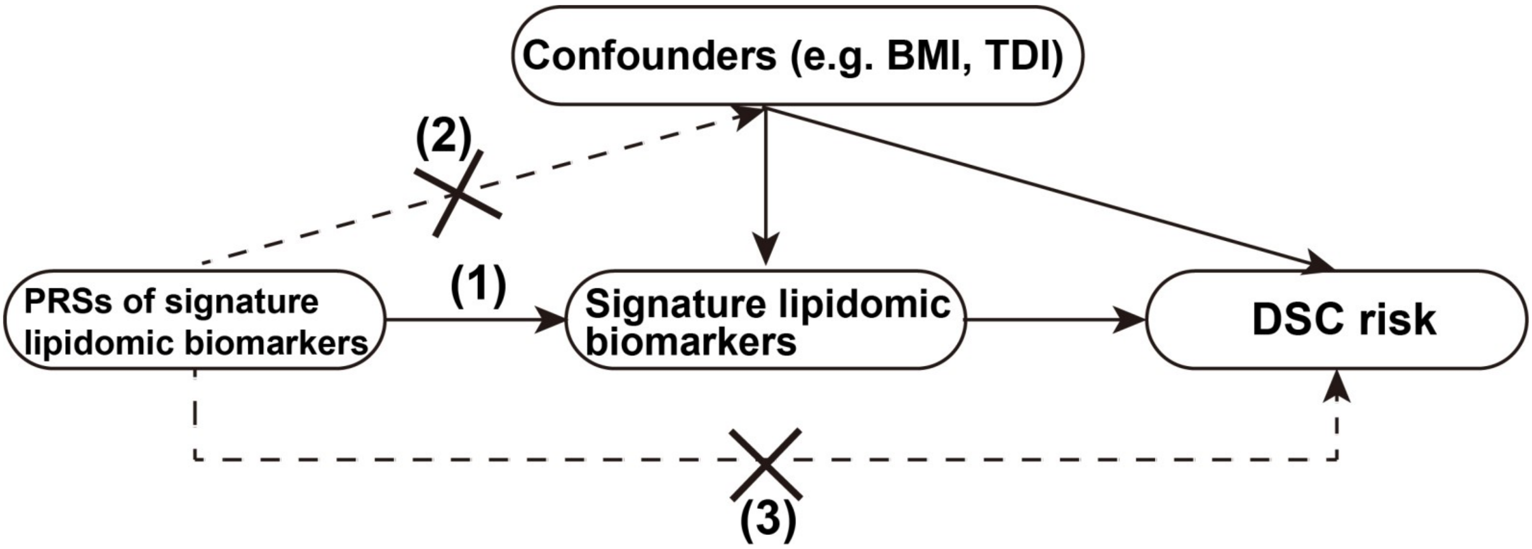

Figure S1. Three key assumptions of MR analyses

Robust causal inferences of signature lipidomic biomarkers on the risk of DSCs from MR analyses were examined with three key assumptions: (1) correlation assumption: HDL-C-PRS, LDL-C-PRS, and TG-PRS were highly correlated with corresponding signature lipidomic biomarker concentrations; (2) independence assumption: HDL-C-PRS, LDL-C-PRS, and TG-PRS were not associated with potential confounders; (3) exclusivity assumption: HDL-C-PRS, LDL-C-PRS, and TG-PRS were only associated with the risk of DSC via corresponding signature lipidomic biomarker concentrations.

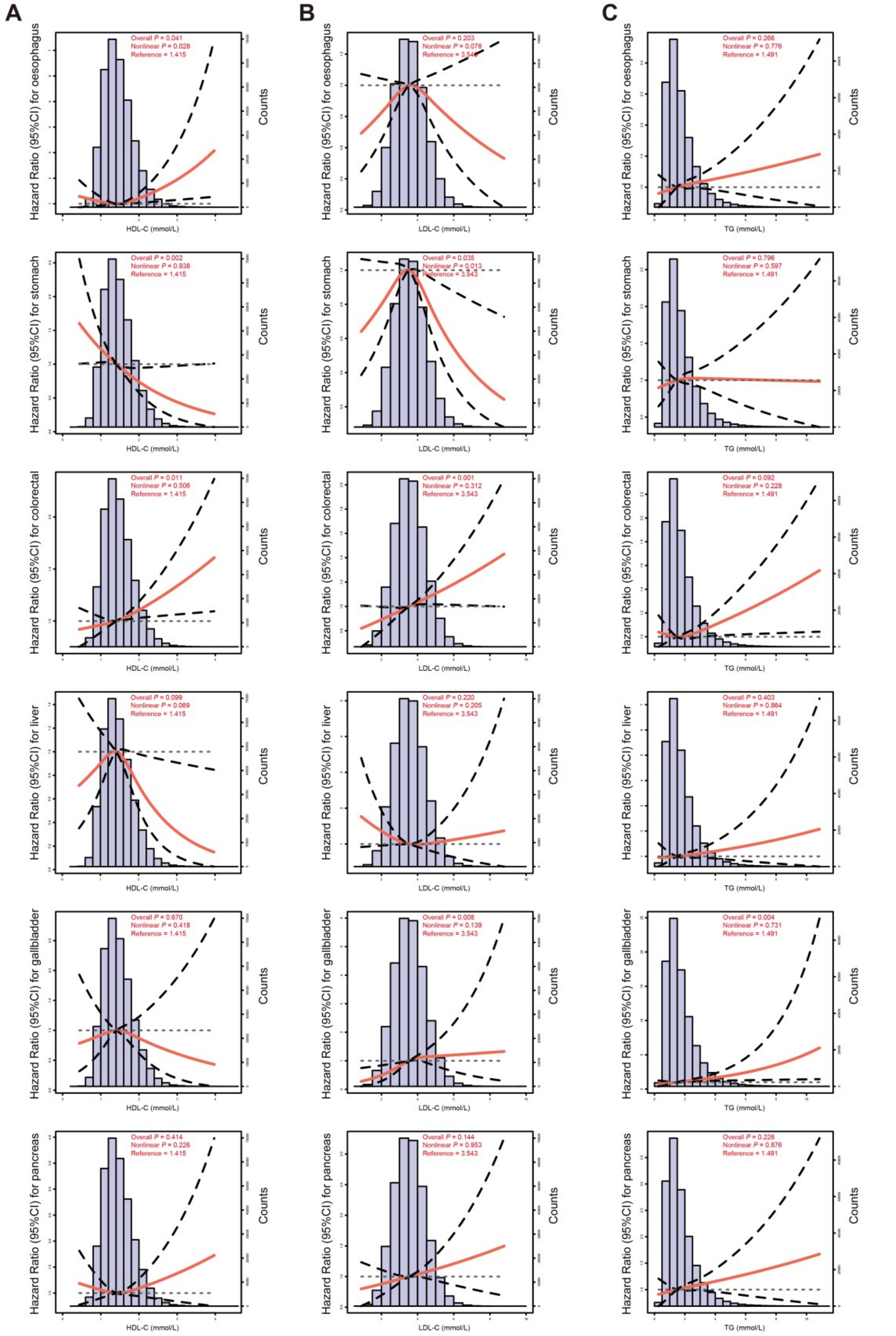

Figure S2. Sensitivity analyses for phenotypic association patterns between signature lipidomic biomarkers (HDL-C (A), LDL-C (B), and TG (C)) and the risk of DSC after excluding DSC participants diagnosed in the first two-year

follow-up time.

Distributions of signature lipidomic biomarker concentrations and RCSs (red lines) representing shapes of phenotypic associations with adjustment of age, sex, BMI, smoking status, alcohol drinking status, education qualification, employment status, TDI, physical activity level, medication use, family cancer history, ALT, AST, SBP, DBP, primary hypertension, cerebral infarction, ischaemic heart disease, and diabetes. $P_{overall}$ and $P_{nonlinear}$ values of 0.008 (0.05/6 outcomes, Bonferroni-adjusted P) were defined as the threshold of remarkable statistical significances. $P_{overall}$ and $P_{nonlinear}$ values between 0.008 and 0.05 were defined as suggestive statistical significances.

RCS, restricted cubic spline; BMI, body mass index; TDI, Townsend deprivation index; ALT, alanine aminotransferase; AST, aspartate aminotransferase; SBP, systolic blood pressure; DBP, diastolic blood pressure; HDL-C, high-density lipoprotein cholesterol; LDL-C, low-density lipoprotein cholesterol; TG, triglycerides

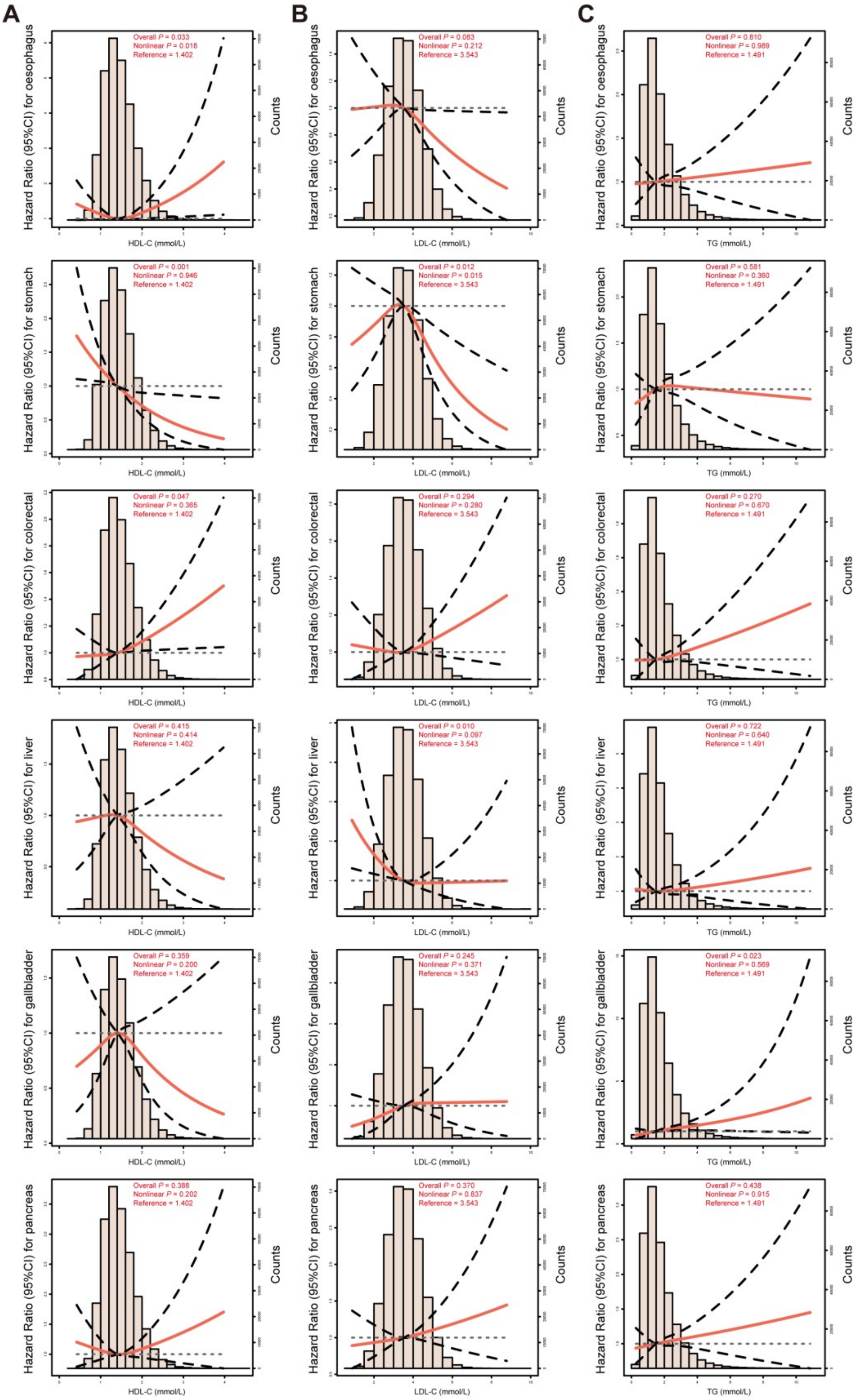

Figure S3. Sensitivity analyses for phenotypic association patterns between signature lipidomic biomarkers (HDL-C (A), LDL-C (B), and TG (C)) and the risk of DSC additionally adjusting for additional medication use.

Distributions of signature lipidomic biomarker concentrations and RCSs (red lines) representing shapes of phenotypic associations with adjustment of age, sex, BMI, smoking status, alcohol drinking status, education qualification, employment status, TDI, physical activity level, medication use, family cancer history, ALT, AST, SBP, DBP, primary hypertension, cerebral infarction, ischaemic heart disease, diabetes, and medication use. $P_{overall}$ and $P_{nonlinear}$ values of 0.008 (0.05/6 outcomes, Bonferroni-adjusted *P*) were defined as the threshold of remarkable statistical significances. $P_{overall}$ and $P_{nonlinear}$ values between 0.008 and 0.05 were defined suggestive statistical significances.

RCS, restricted cubic spline; BMI, body mass index; TDI, Townsend deprivation index; ALT, alanine aminotransferase; AST, aspartate aminotransferase; SBP, systolic blood pressure; DBP, diastolic blood pressure; HDL-C, high-density lipoprotein cholesterol; LDL-C, low-density lipoprotein cholesterol; TG, triglycerides

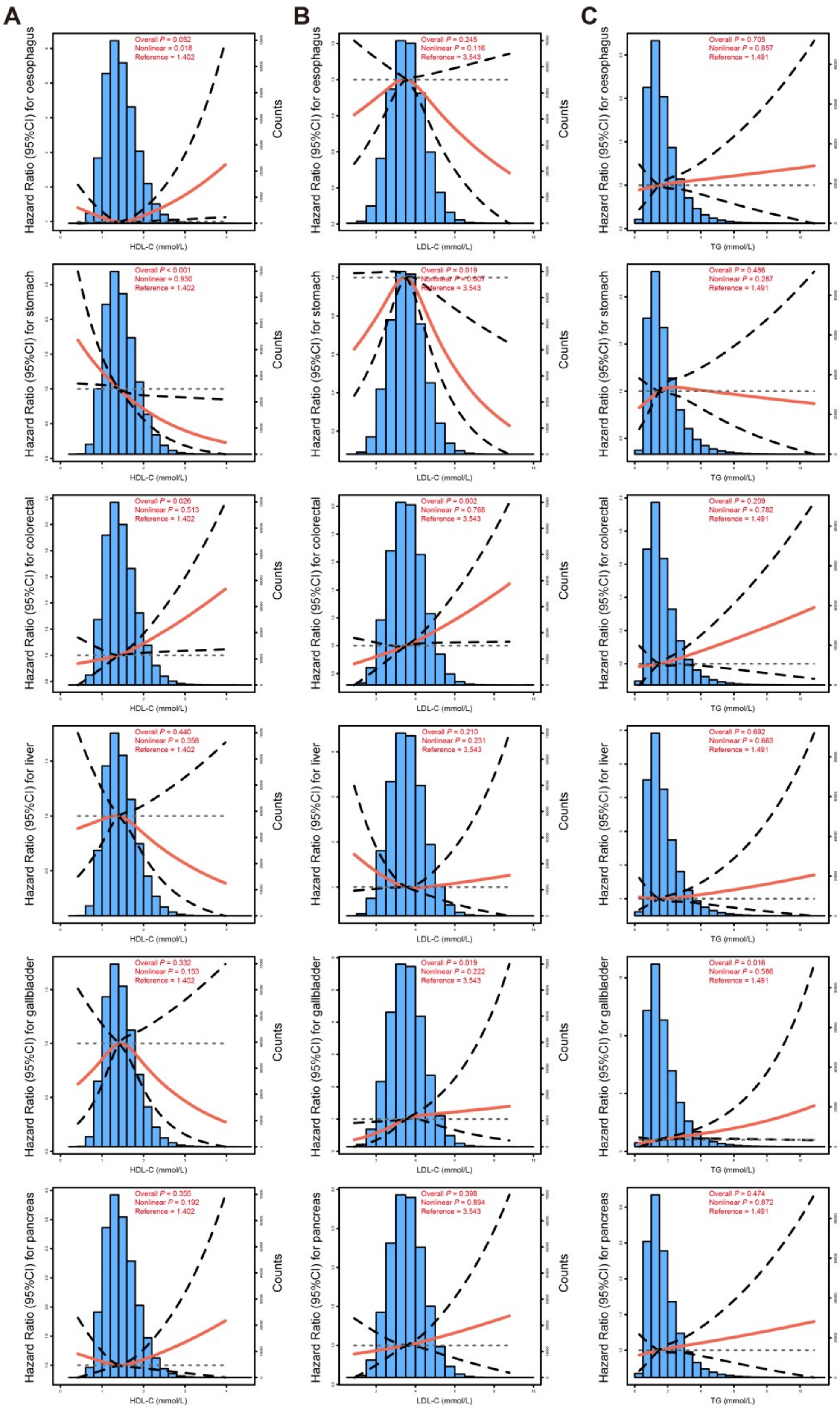

Figure S4. Sensitivity analyses for phenotypic association patterns between signature lipidomic biomarkers (HDL-C (A), LDL-C (B), and TG (C)) and the risk of DSC additionally adjusting for fasting time

Distributions of signature lipidomic biomarker concentrations and RCSs (red lines) representing shapes of phenotypic associations with adjustment of age, sex, BMI, smoking status, alcohol drinking status, education qualification, employment status, TDI, physical activity level, medication use, and family cancer history, ALT, AST, SBP, DBP, primary hypertension, cerebral infarction, ischaemic heart disease, diabetes, and fasting time. $P_{overall}$ and $P_{nonlinear}$ values of 0.008 (0.05/6 outcomes, Bonferroni-adjusted *P*) were defined as the threshold of remarkable statistical significances. $P_{overall}$ and $P_{nonlinear}$ values between 0.008 and 0.05 were defined as suggestive statistical significances.

RCS, restricted cubic spline; BMI, body mass index; TDI, Townsend deprivation index; ALT, alanine aminotransferase; AST, aspartate aminotransferase; SBP, systolic blood pressure; DBP, diastolic blood pressure; HDL-C, high-density lipoprotein cholesterol; LDL-C, low-density lipoprotein cholesterol; TG, triglycerides

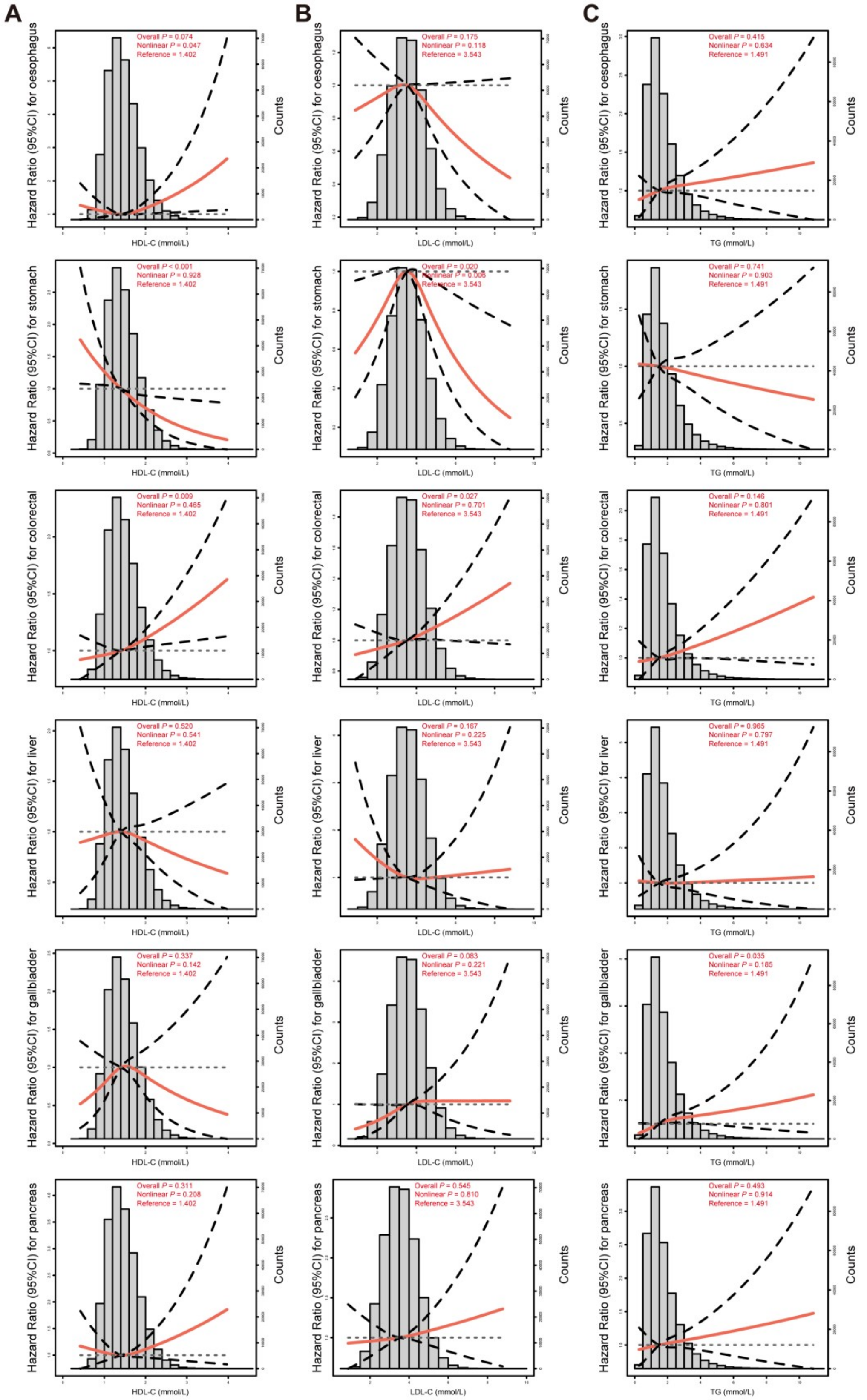

Figure S5. Sensitivity analyses for phenotypic association patterns between signature lipidomic biomarkers (HDL-C (A), LDL-C (B), and TG (C)) and the risk of DSC additionally adjusting for signature lipidomic biomarkers.

Distributions of signature lipidomic biomarker concentrations and RCSs (red lines) representing shapes of phenotypic associations with adjustment of age, sex, BMI, smoking status, alcohol drinking status, education qualification, employment status, TDI, physical activity level, medication use, and family cancer history, ALT, AST, SBP, DBP, primary hypertension, cerebral infarction, ischaemic heart disease, diabetes, and signature lipidomic biomarkers (for HDL-C: with adjustment of LDL-C and TG; for LDL-C: with adjustment of HDL-C and TG; for TG: with adjustment of HDL-C and LDL-C). $P_{overall}$ and $P_{nonlinear}$ values of 0.008 (0.05/6 outcomes, Bonferroni-adjusted *P*) were defined as the threshold of remarkable statistical significances. $P_{overall}$ and $P_{nonlinear}$ values between 0.008 and 0.05 were defined as suggestive statistical significances.

BMI, body mass index; TDI, Townsend deprivation index; ALT, alanine aminotransferase; AST, aspartate aminotransferase; SBP, systolic blood pressure; DBP, diastolic blood pressure HbA1c, glycated haemoglobin; HDL-C, high-density lipoprotein cholesterol; LDL-C, low-density lipoprotein cholesterol; TG, triglycerides

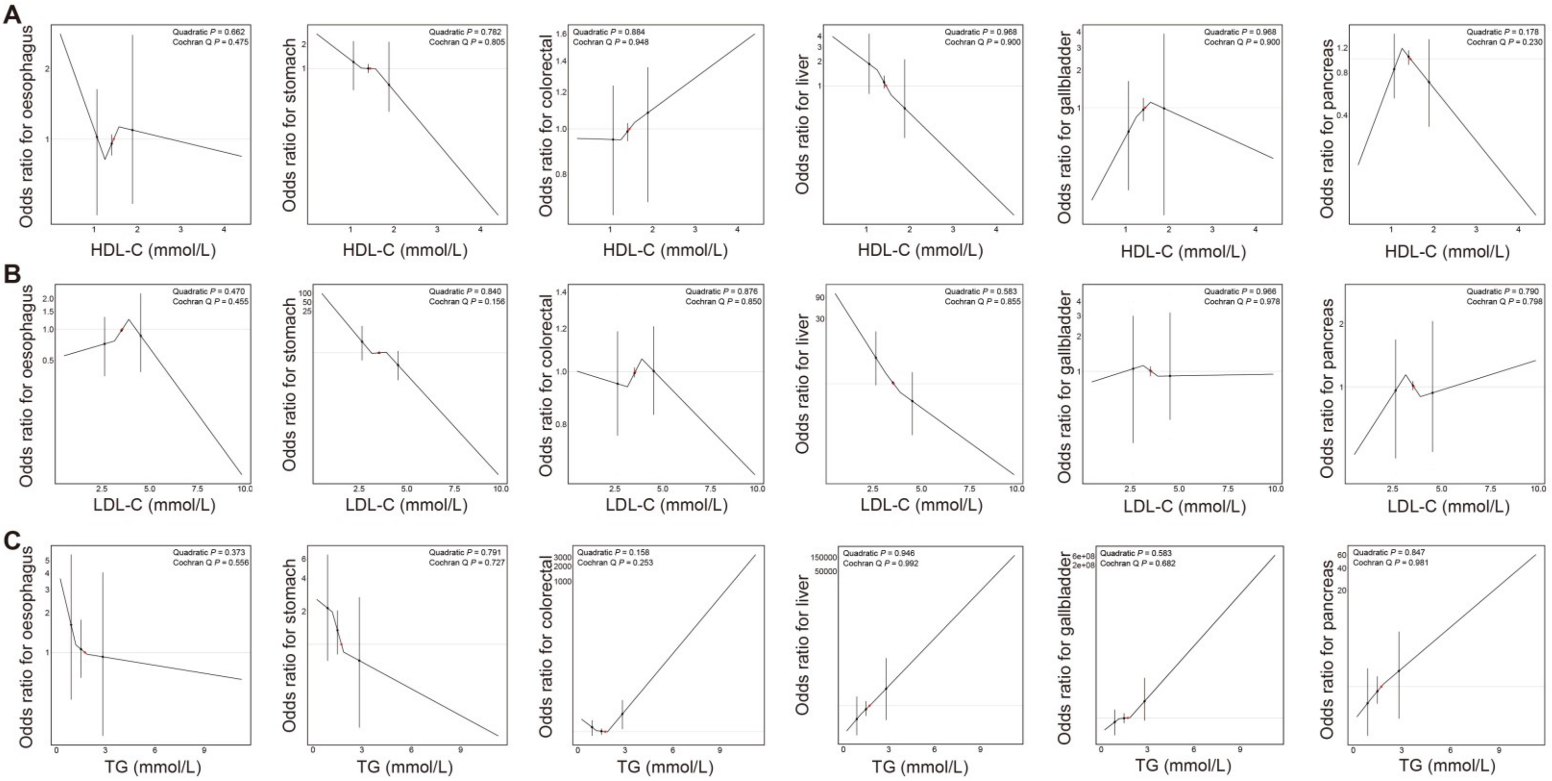

Figure S6. Causal association patterns between signature lipidomic biomarkers and the risk of DSCs with adjustment of potential confounders.

Nonlinear MR analyses with piecewise linear method for genetically predicting the associations between (A) HDL-C, (B) LDL-C, and (C) TG concentrations and the risk of oesophagus, stomach, colorectal, liver, gallbladder, and pancreas cancers. Exposure and outcome regression stages were both adjusted age, sex, assessment centers, genotyping array, the first 10 PCs, and potential confounders (for HDL-C: additionally adjusted for SBP, ischaemic heart disease, primary hypertension, and diabetes; for LDL-C: additionally adjusted for ischaemic heart disease and HbA1c; for TG: additionally adjusted for SBP, DBP, ischaemic heart disease, primary hypertension, HbA1c, and diabetes). Each black dot and black vertical line represented the LACE with its 95% confidence interval in each stratum and red dots represent reference points.

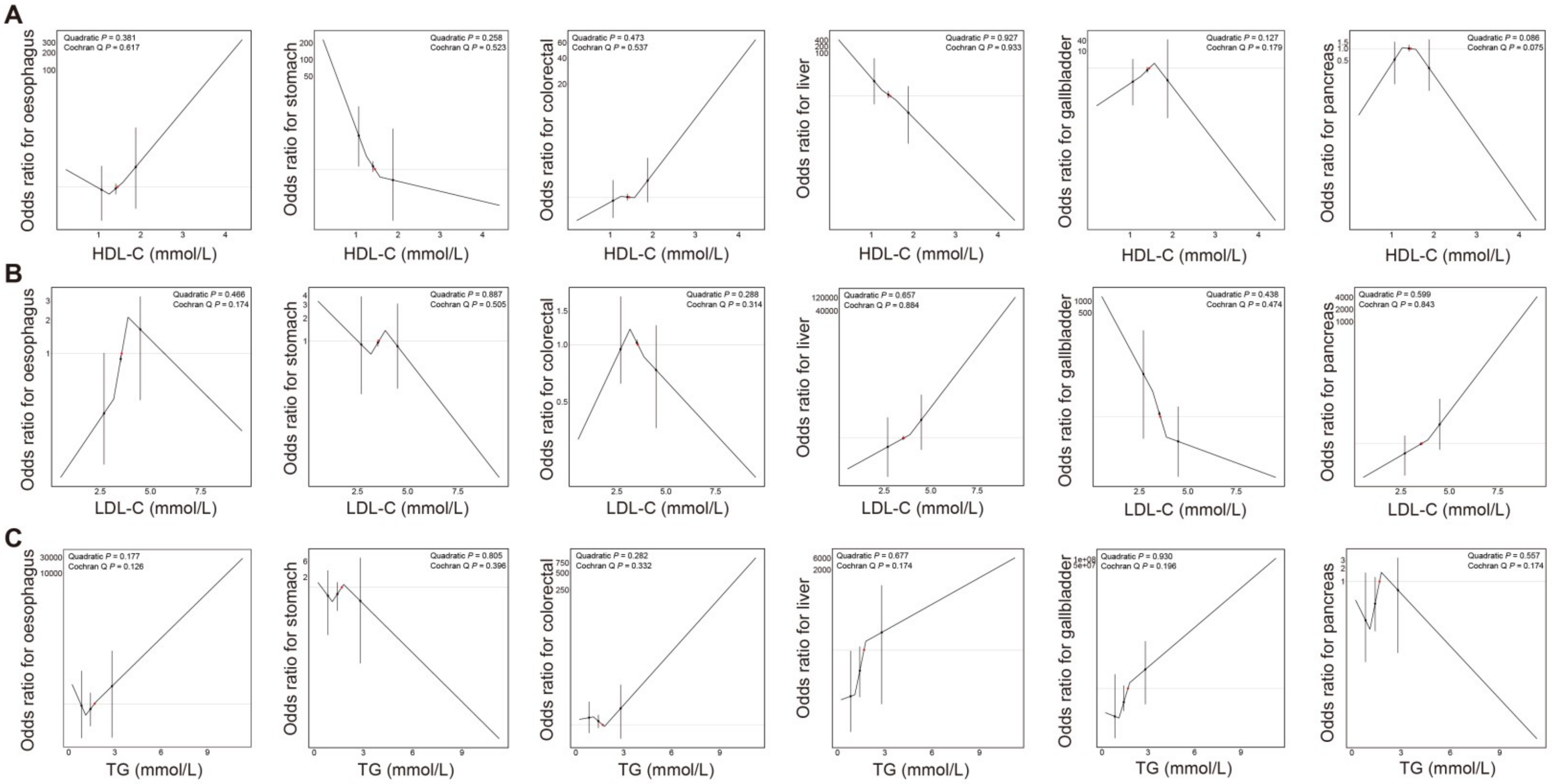

Figure S7. Causal association patterns between signature lipidomic biomarkers and the risk of DSCs in participants aged less than 60 years.

Nonlinear MR analyses with piecewise linear method for genetically predicting the associations between (A) HDL-C, (B) LDL-C, and (C) TG concentrations and the risk of oesophagus, stomach, colorectal, liver, gallbladder, and pancreas cancers in participants aged less than 60 years. Exposure and outcome regression stages were both adjusted with sex, assessment centers, genotyping array and the first 10 PCs. Each black dot and black vertical line represented the LACE with its 95% confidence interval in each stratum and red dots represent reference points.

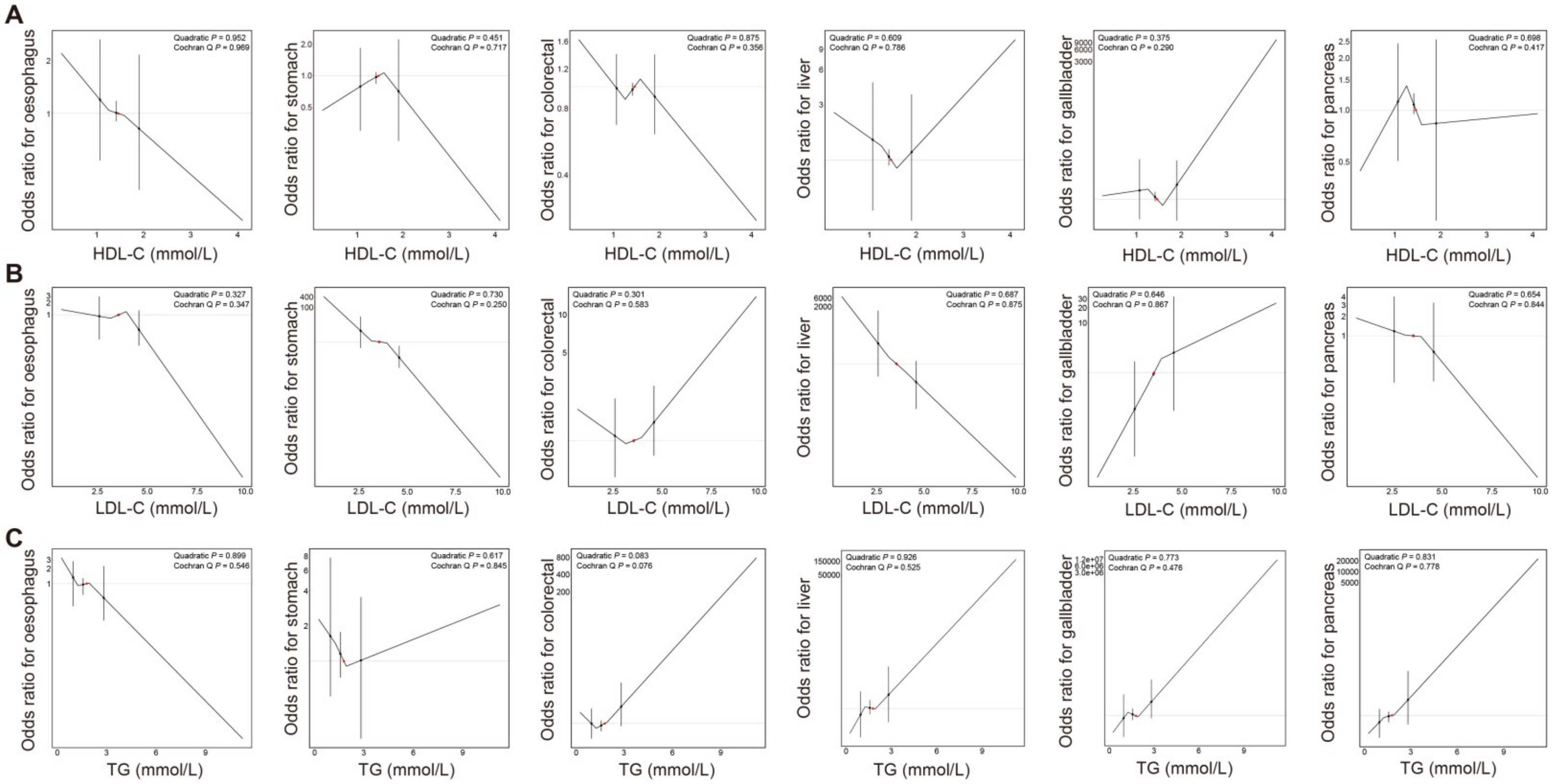

Figure S8. Causal association patterns between signature lipidomic biomarkers and the risk of DSCs in participants aged 60 years or older.

Nonlinear MR analyses with piecewise linear method for genetically predicting the associations between (A) HDL-C, (B) LDL-C, and (C) TG concentrations and the risk of oesophagus, stomach, colorectal, liver, gallbladder, and pancreas cancers in participants aged 60 years or older. Exposure and outcome regression stages were both adjusted with sex, assessment centers, genotyping array and the first 10 PCs. Each black dot and black vertical line represented the LACE with its 95% confidence interval in each stratum and red dots represent reference points.

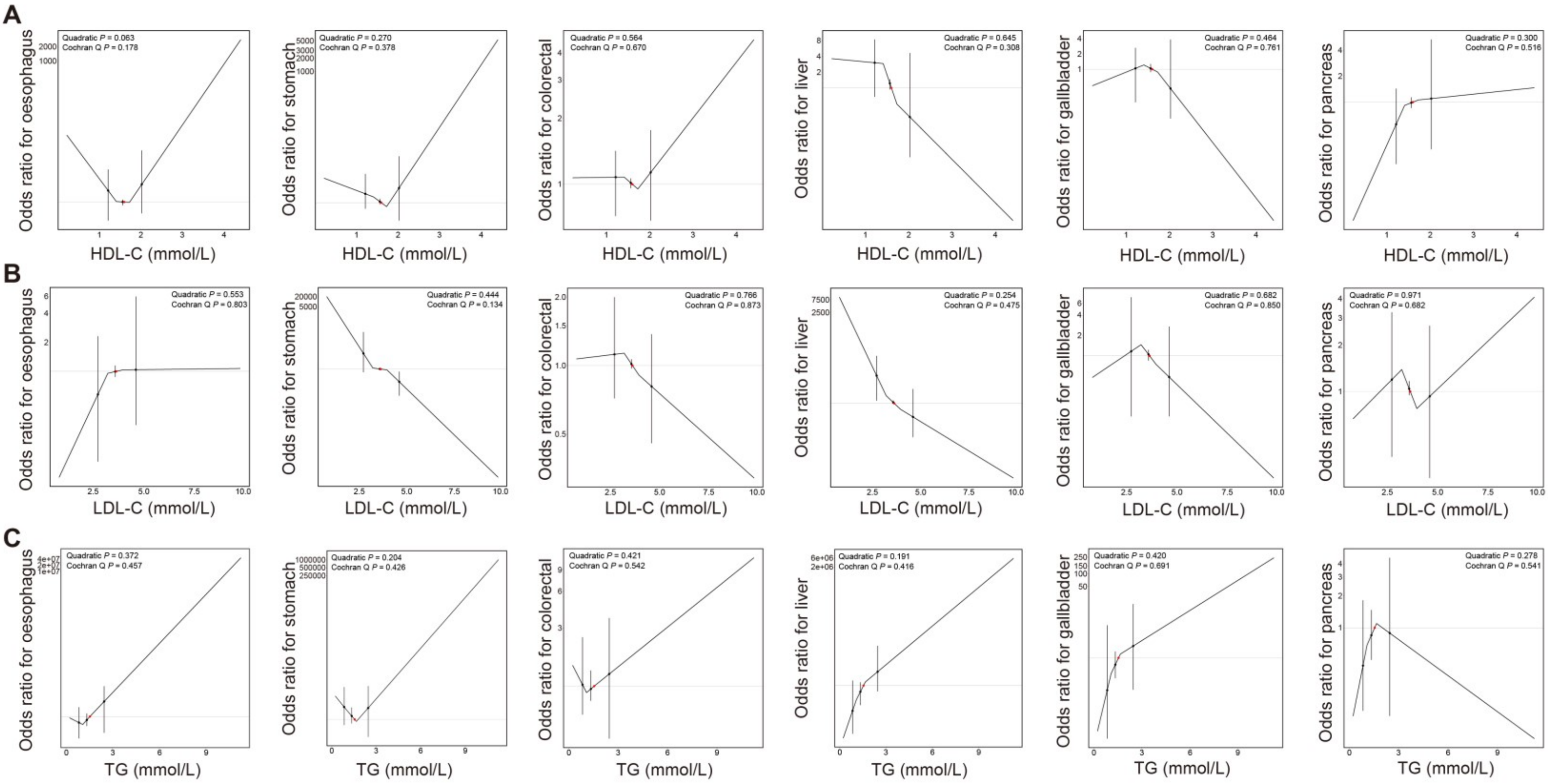

Figure S9. Causal association patterns between signature lipidomic biomarkers and the risk of DSCs in female participants.

Nonlinear MR analyses with piecewise linear method for genetically predicting the associations between (A) HDL-C, (B) LDL-C, and (C) TG concentrations and the risk of oesophagus, stomach, colorectal, liver, gallbladder, and pancreas cancers in female participants. Exposure and outcome regression stages were both adjusted with sex, assessment centers, genotyping array and the first 10 PCs. Each black dot and black vertical line represented the LACE with its 95% confidence interval in each stratum and red dots represent reference points.

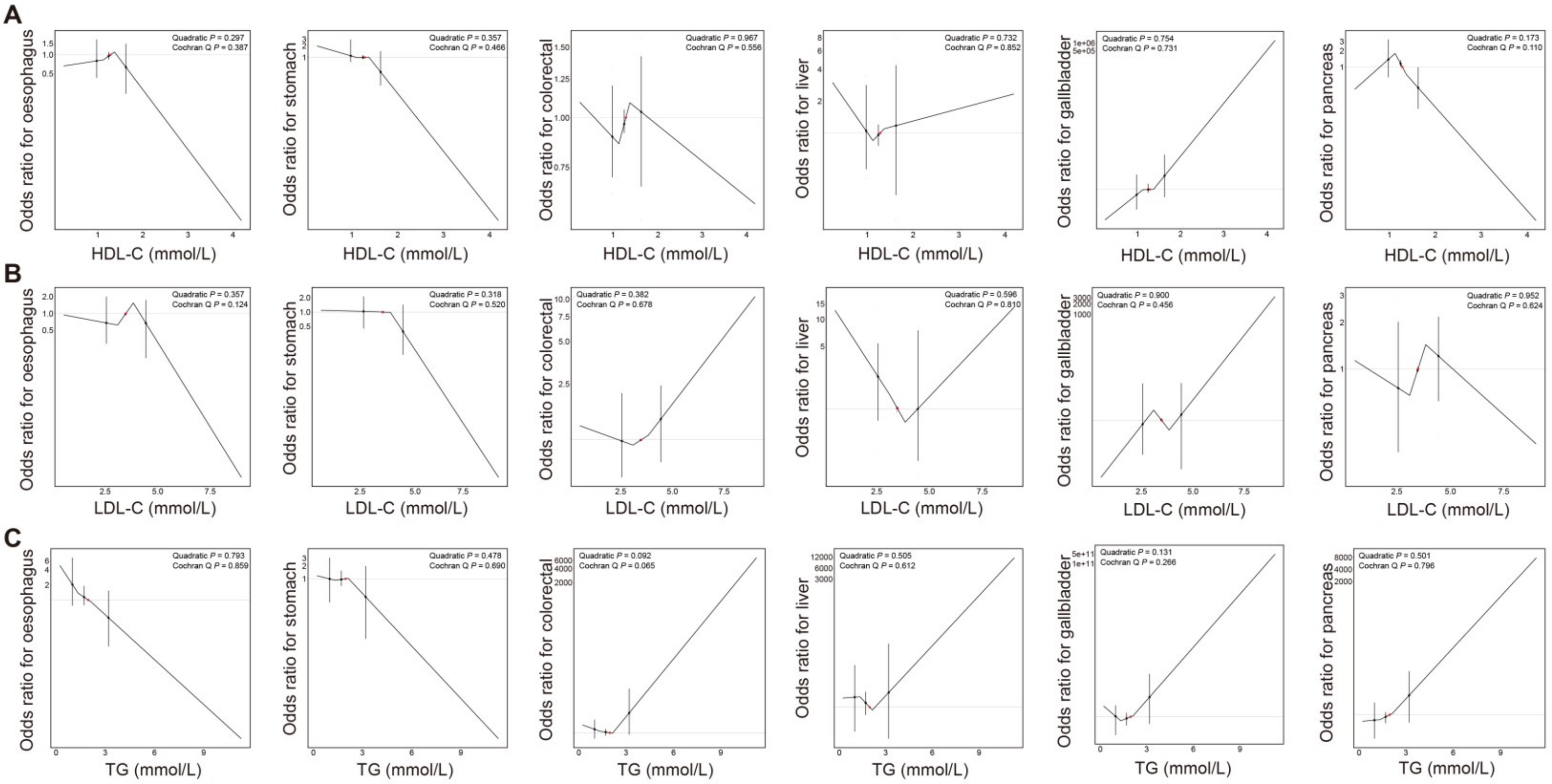

Figure S10. Causal association patterns between signature lipidomic biomarkers and the risk of DSCs in male participants.

Nonlinear MR analyses with piecewise linear method for genetically predicting the associations between (A) HDL-C, (B) LDL-C, and (C) TG concentrations and the risk of oesophagus, stomach, colorectal, liver, gallbladder, and pancreas cancers in male participants. Exposure and outcome regression stages were both adjusted with sex, assessment centers, genotyping array and the first 10 PCs. Each black dot and black vertical line represented the LACE with its 95% confidence interval in each stratum and red dots represent reference points.
